# Dysfunction of the paraventricular thalamus–prelimbic cortex circuit underlies maternal separation–induced deficits in contagious pain

**DOI:** 10.1126/sciadv.ady1944

**Published:** 2025-10-08

**Authors:** Zichen Zhang, Yu Gong, Xin-Yu Su, Yujiao Deng, Rui Du, Chun-Li Li, Yan Wang, Xing-Lei Song, Qin Jiang, Ying Li, Yujin Jian, Xiaoping Tong, Guang-Hai Wang, Jun Chen, Tian-Le Xu, Ming-Gang Liu, Fan Jiang

**Affiliations:** ^1^ Department of Developmental and Behavioral Pediatrics, Pediatric Translational Medicine Institute, Shanghai Key Laboratory of Child Brain and Development, National Children’s Medical Center, Shanghai Children’s Medical Center, Shanghai Jiao Tong University School of Medicine, Shanghai 200127, China.; ^2^ Department of Anatomy and Physiology, Shanghai Jiao Tong University School of Medicine, Shanghai 200025, China.; ^3^ Institute for Biomedical Sciences of Pain, Tangdu Hospital, Fourth Military Medical University, Xi’an, Shaanxi Province 710038, China.; ^4^ MOE-Shanghai Key Laboratory of Children’s Environmental Health, Xinhua Hospital, Shanghai Jiao Tong University School of Medicine, Shanghai 200092, China.; ^5^ Sanhang Institute for Brain Science and Technology (SiBST), Northwestern Polytechnical University (NPU), Xi’an, Shaanxi Province 710129, China.; ^6^ Department of Anesthesiology, Songjiang Hospital and Songjiang Research Institute, Shanghai Key Laboratory of Emotions and Affective Disorders, Shanghai Jiao Tong University School of Medicine, Shanghai 2016099, China.; ^7^ Institute of Mental Health and Drug Discovery, Oujiang Laboratory (Zhejiang Lab for Regenerative Medicine, Vision and Brain Health), Wenzhou, Zhejiang 325000, China.

## Abstract

Contagious pain is considered one of the most common forms of emotional contagion observed in animal models. Nevertheless, little is known about the precise neural mechanisms governing the regulation of contagious pain in response to diverse environmental stressors. Here, we report that early life maternal separation (MS) precipitates impairments in the pain contagion between familiar partners. Specifically, we identify the indispensable role of glutamatergic projections from the paraventricular thalamus (PVT) to the prelimbic cortex (PrL) for the development of vicarious pain hypersensitivity. MS dampens activation of the PVT → PrL pathway during social interactions between observer and painful demonstrator. Augmenting the excitability or activity of the PVT → PrL circuit through chemogenetic interventions or tactile stimulation resembling social touch significantly ameliorates the MS-evoked contagious pain deficits. Collectively, our findings delineate a neural circuitry substrate underlying the loss of contagious pain stemming from MS and propose a potential therapeutic avenue for mitigating empathic impairments associated with early life adversity.

## Teaser

The thalamocortical circuit dysfunction underlies maternal separation–induced contagious pain deficits.

## INTRODUCTION

Empathy is widely recognized as the capacity to perceive and respond to the emotions and experiences of others, enabling individuals to share their feelings and understand their perspectives (*1*, *2*). It ranges from basic emotional contagion to more complex manifestations such as sympathetic concern, consolation behavior, and targeted helping, as well as perspective taking (*3*–*5*). Emotional contagion involves the cognitive process of sensing and sharing the emotional states of others. Among its various forms, empathic contagious pain holds particular significance in eliciting prosocial behaviors (*1*, *6*–*8*). It is now understood that rodents exhibit evolutionarily conserved forms of affective empathy (*9*, *10*), prompting an increasing number of investigations into the neuronal and circuitry mechanisms underlying contagious pain (*11*–*14*). However, our understanding of the neural processes governing the development and regulation of pain contagion remains incomplete.

Exposure to traumatic events during early life, such as child neglect or physical abuse, poses a significant risk for the emergence of maladaptive behaviors in adulthood. Maternal separation (MS) during the initial postnatal weeks stands out as a prominent form of early life stress in mice, leading to lasting impairments in emotional and cognitive behaviors (*15*, *16*). Human cohort studies have consistently demonstrated the profound influence of maternal responsiveness and caregiving patterns on the development of empathy-related competencies in the early years of life (*17*, *18*). Similarly, a growing body of animal research highlights that early life stressors, including MS, heighten susceptibility to emotional and social dysfunctions later in life (*19*–*22*). Nevertheless, the specific neural substrates and the intricate neurocircuitry mechanisms through which early life stressors translate into altered neuronal activity, ultimately culminating in empathic dysfunction during adulthood, remain incompletely understood.

The paraventricular thalamus (PVT) constitutes a midline thalamic nucleus renowned for its extensive connectivity with various forebrain, midbrain, and hindbrain regions, positioning it strategically to integrate information pertinent to behavioral significance (*23*). Recent investigations using sophisticated methodologies for monitoring and manipulating neuronal activity have furnished causal evidence linking the PVT to the orchestration of emotional and motivated behaviors (*24*, *25*). Notably, emerging evidence suggests a potential role of the PVT in mediating empathic consolation exhibited by observers toward familiar demonstrator mice undergoing a common artery exposure surgery (*26*). However, the precise involvement of the PVT in empathic contagious pain and the underlying circuitry elements remain uncertain.

Here, we identify PVT glutamate neurons as a key node mediating the adverse effects of early life stress on empathic behavior. Using an early MS paradigm, we revealed a significant down-regulation in the activity and responsiveness of PVT glutamate neurons to vicariously transmitted negative emotional signals in mice exposed to MS, associated with a diminished social transfer of pain. Chemogenetic activation of PVT glutamate neurons or their projection to the medial prefrontal cortex (mPFC), particularly the prelimbic cortex (PrL), markedly mitigates the MS-evoked impairment in pain contagion. Social touch (ST)–like tactile stimulation, a widely acknowledged paradigm encoding positive hedonic valence and fostering affiliative behaviors (*27*–*31*), effectively restores the impaired contagious pain in MS mice by enhancing the activation of the PVT → PrL projection circuit. Collectively, our findings underscore the critical involvement of PVT glutamate neurons and their projections to the PrL in the contagious pain. Early life MS attenuates the activation of PVT neurons and associated circuits, thereby impeding the expression of contagious pain. This study holds the potential to provide insights into understanding how maternal behavior shapes the emotional contagion.

## RESULTS

### Early life MS impairs contagious pain in adulthood

**Fig. 1.**
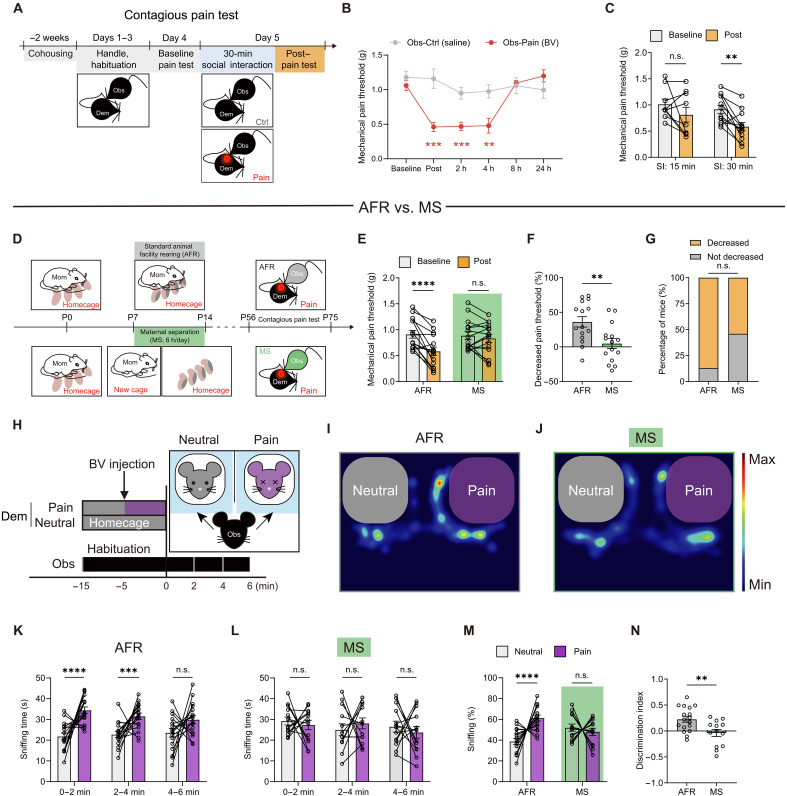
Early life MS impairs the contagious pain in adulthood. (**A**) Schematic timeline for the contagious pain paradigm. Dem, demonstrator. (**B**) Time course analysis of the social transfer of pain. Obs-Ctrl: *n* = 12 mice; Obs-Pain: *n* = 12 mice. **P < 0.01 and ***P < 0.001; two-way repeated-measures analysis of variance (RM ANOVA). h, hours. (**C**) Mechanical pain threshold of the observer at both baseline and after 30 min of social interaction (SI). SI for 15 min: *n* = 9 mice; SI for 30 min: *n* = 12 mice. **P < 0.01; two-way RM ANOVA. (**D**) Schematic of the standard animal facility rearing (AFR) and MS protocol. (**E**) Mechanical pain threshold of AFR and MS mice. *n* = 15 mice for each group. ****P < 0.0001; two-way RM ANOVA. (**F**) Percentage decrease in mechanical pain threshold. **P < 0.01; unpaired *t* test. (**G**) Percentage of AFR and MS mice with or without a decrease in pain threshold. (**H**) Schematic of the emotional discrimination task. (**I** and **J**) Representative heatmaps depicting the amount of time spent by either AFR (I) or MS (J) observers in different locations of the apparatus. (**K** and **L**) Sniffing time of AFR (K) and MS observers (L) toward neutral or painful demonstrators in 2-min bins. AFR: *n* = 17 mice; MS: *n* = 13 mice. ***P < 0.001 and ****P < 0.0001; two-way RM ANOVA. (**M**) Percentage of sniffing time in the first 2 min. AFR: *n* = 17 mice; MS: *n* = 13 mice. ****P < 0.0001; generalized linear mixed model. (**N**) Discrimination index derived from (M). **P < 0.01; unpaired *t* test. n.s., no significant difference. Data are presented as means ± SEM.

To investigate the impact of MS on empathic behavior later in life, we initially established an animal model of contagious pain, as previously described (*32*, *33*). Briefly, the observer mouse was cohoused with a demonstrator mouse for 2 weeks to establish familiarity (Fig. 1A). Following handling and baseline pain tests, the observer mouse was permitted to freely interact with a familiar demonstrator that had undergone intraplantar injection of bee venom (BV), inducing persistent inflammatory pain [observer-pain (Obs-Pain)]. The control demonstrator received an equivalent volume of physiological saline solution injection into the left hind paw [observer-control (Obs-Ctrl)]. Following a 30-min social interaction with the BV-inflamed demonstrator, the observer mouse exhibited a significant mechanical hypersensitivity lasting at least 4 hours (Fig. 1B), evidenced by a notable and sustained decrease in mechanical pain threshold (see Materials and Methods for detailed pain behavioral test procedures). Clear evidence of pain transfer was not observed when the observer interacted with the painful demonstrator for only 15 min (Fig. 1C) or when interacting with the control demonstrator for 30 min (Fig. 1B).

We then implemented a modified version of the MS paradigm, where mouse pups at postnatal day 7 (P7) were subjected to daily 6-hour separations from their dam for seven consecutive days (Fig. 1D). Control mice underwent standard animal facility rearing (AFR) throughout the experimental period. Adult mice (8 to 11 weeks old) from both the AFR- and MS-treated groups were used as observers to evaluate any alterations in contagious pain response. While AFR-treated mice exhibited vicarious pain hypersensitivity following a 30-min interaction with a familiar BV-inflamed demonstrator, MS-treated animals displayed significant deficits in the social transfer of pain (Fig. 1E), despite the absence of apparent effects on baseline pain threshold. This impairment became more evident when comparing the percentage decrease in threshold between baseline and post–mechanical pain tests (see Materials and Methods for calculation; Fig. 1F), as well as the proportion of mice displaying a decreased or nondecreased pain threshold (Fig. 1G). As a control, we found that early life MS did not affect the other baseline pain measurements, such as heat pain sensitivity assessed by the hot plate test and capsaicin-induced nociceptive behaviors (fig. S1).

Next, we sought to determine whether MS treatment at other periods equally elicited an impairment of pain contagion. To this end, we performed a similar experiment as described above but on animals subjected to MS at P2 to P7 or P15 to P21. The results show that MS at P2 to P7 could still block the occurrence of contagious pain in adulthood (fig. S2, A to D), whereas MS at P15 to P21 only induced a mild detrimental effect, with the animal still exhibiting a decreased pain threshold after social interaction with the familiar demonstrator (fig. S2, E to H). This finding indicates the existence of a critical time window for the MS to adversely influence the pain contagion.

One possibility that cannot be excluded is that MS mice may require a longer social interaction time to induce social transfer of pain (*13*). Thus, we repeated the experiment shown in Fig. 1D but now extended the observer-demonstrator interaction period from 30 min to 1 hour (fig. S2I). Although comparison of the post– versus pre–mechanical pain threshold demonstrated a significant decrease in the MS mice, the decrease extent and the percentage of mice showing contagious pain still revealed a statistical difference between control and MS groups of mice (fig. S2, J to L). These results suggest that MS still induced a significant deficit in the contagious pain even when the observer is allowed to interact with the painful demonstrator for 1 hour. In addition, to probe any sex-related difference in the above effect, we replicated the experiment on female mice subjected to MS at P7 to P14. No significant sex difference was detected in the effect of MS on socially transferred pain (fig. S3, A to C). Furthermore, MS-evoked contagious pain deficits are not due to any changes in anxiety-like traits, sociability, social novelty, or locomotor function (fig. S4).

According to the contemporary perspective, affective preference, or emotional discrimination, serves as a prerequisite for various forms of empathic behaviors, including emotional contagion and consolation (*2*, *3*). Observers must initially recognize the distressed state of demonstrators and exhibit clear preference toward emotionally stressed individuals before engaging in subsequent prosocial actions. To investigate how MS influences the process of social pain transfer, we explored whether MS could result in deficits in emotional discrimination. Here, we modified a previously described experimental paradigm (*34*–*36*) to assess whether AFR- or MS-treated observers could differentiate between neutral and painful demonstrators (Fig. 1H). Briefly, the observer mouse was positioned in front of two demonstrators, matched for sex and age, within two separate chambers. One demonstrator received a subcutaneous injection of BV solution to induce alteration in affective state, while the neutral demonstrator was an untreated naïve mouse. Following habituation to the testing arena, we presented the painful and neutral unfamiliar demonstrators to an observer.

As anticipated, AFR control observers exhibited increased sniffing toward the stressed conspecific compared to the neutral demonstrator (Fig. 1I), particularly within the first 2 min of observation (Fig. 1K). These findings suggest that AFR mice could discern unfamiliar conspecifics based on their affective states. However, MS mice failed to detect and respond to conspecifics in a negatively valenced emotional state (Fig. 1, J to L). To further illustrate this deficit in emotional discrimination, we calculated the percentage of sniffing time within the first 2 min of the test and a discrimination index (see Materials and Methods for the formula), yielding consistent results (Fig. 1, M and N). A similar impairment in affective state discrimination was observed in female observers subjected to MS during early postnatal development (fig. S3, D and E). Thus, the compromised ability for affective state discrimination most likely contributes to the MS-induced deficits in contagious pain.

### PVT neuronal activity contributes to the development of contagious pain

To identify the specific brain regions implicated in MS-induced deficits in contagious pain, we assessed the expression of the neuronal activation marker *c-fos* across various brain areas in response to painful demonstrators. For this purpose, we used a reporter line generated by crossing FosCreER^T2^ (TRAP2) mice (*37*) with Ai9-tdTomato reporter mice, enabling visualization of *c-fos*–driven neuronal activation through tdTomato fluorescence following intraperitoneal injection of 4-hydroxytamoxifen (4-OHT) 30 min before a 90-min social interaction between observer and demonstrator (*13*). Quantitative analysis revealed that exposure of observer mice to BV-inflamed demonstrators led to significant activation of multiple brain regions, including the mPFC, anterior cingulate cortex, insular cortex, nucleus accumbens (NAc), medial preoptic area, paraventricular nucleus of the hypothalamus (PVN), and PVT (fig. S5, A and B). However, MS treatment selectively inhibited the activation of two candidate regions: PVN and PVT (fig. S5, C and D).

**Fig. 2.**
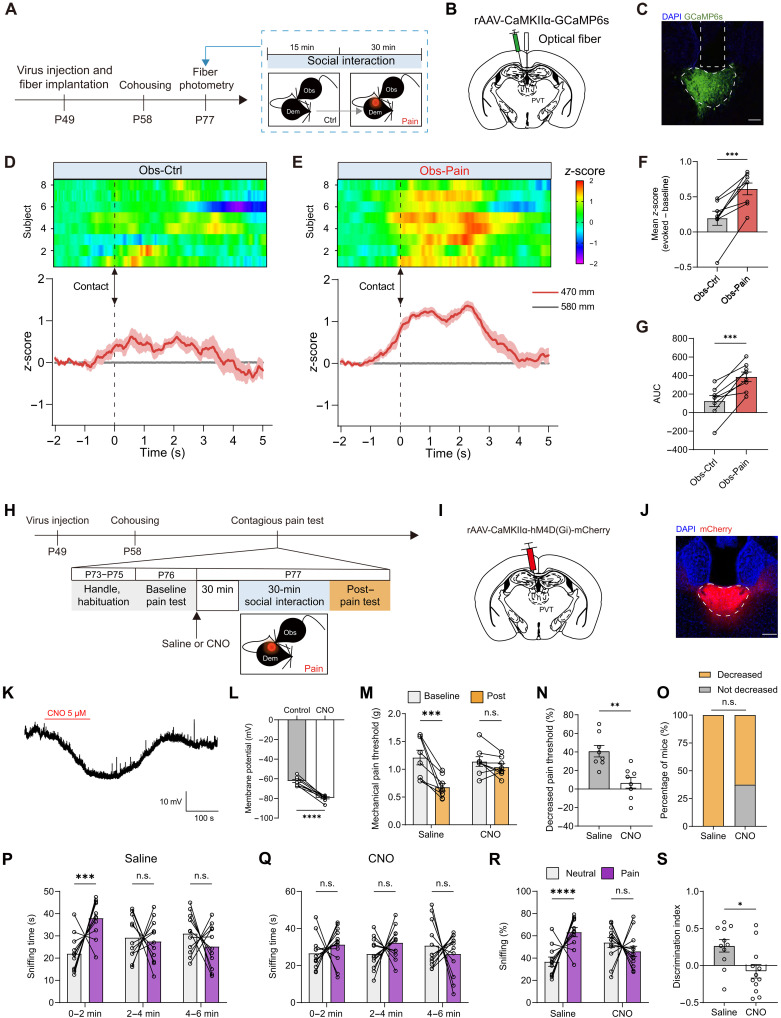
Involvement of PVT glutamate neurons in the contagious pain. (**A**) Schematic for fiber photometry recording of PVT neurons in the AFR mice. (**B** and **C**) Schematic (B) and histological verification (C) of injection of AAV-CaMKIIα-GCaMP6s into the PVT and implantation of optical fiber into the PVT. Scale bar, 100 μm. (**D** and **E**) Heatmap (top) and peristimulus time histogram (bottom) of Ca^2+^ signals aligned with the onset of body contact between AFR observers and control (D) or painful demonstrators (E). (**F** and **G**) Quantification of the change in GCaMP6s signal as either mean *z*-score (F) or averaged area under curve (AUC) (G). *n* = 8 mice. ***P < 0.001; paired *t* test. (**H**) Schematic timeline for (I) to (O). (**I** and **J**) Schematic (I) and histological verification (J) of injection of AAV-CaMKIIα-hM4D(Gi)-mCherry into the PVT. Scale bar, 100 μm. (**K** and **L**) Representative traces (K) and quantification (L) of clozapine-*N-*oxide (CNO) (5 μM)–evoked membrane hyperpolarization. *n* = 8 neurons from three mice. ****P < 0.0001; paired *t* test. (**M**) Changes in the mechanical pain threshold of AFR observers. *n* = 8 mice for each group. ***P < 0.001; two-way RM ANOVA. (**N**) Percentage decrease in mechanical pain threshold. **P < 0.01; unpaired *t* test. (**O**) Percentage of AFR mice with or without contagious pain. (**P** and **Q**) Sniffing time of saline (P) and CNO observers (Q) toward neutral or painful demonstrators in 2-min bins. Saline: *n* = 11 mice; CNO: *n* = 12 mice. ***P < 0.001; two-way RM ANOVA. (**R** and **S**) Percentage of sniffing time (R) and discrimination index (S) in the first 2 min of the emotional discrimination task. (R) ****P < 0.0001; generalized linear mixed model. (S) *P < 0.05; unpaired *t* test. Data are presented as means ± SEM.

PVT occupies a pivotal position, receiving extensive inputs from the hypothalamus and brainstem, which convey signals regarding motivational arousal and homeostatic states (*25*, *38*). These anatomical connections position the PVT as a crucial hub for modulating various behavioral responses. Building upon the findings from TRAP2: Ai9 mice (fig. S5), we sought to investigate the potential involvement of PVT neurons in the pain contagion. Because the PVT is predominantly composed of glutamate neurons (*39*, *40*), we first asked whether the activity of PVT glutamate neurons was altered during social interactions between observers and familiar demonstrators using fiber photometry. AFR control mice received injections of adeno-associated viruses (AAVs) carrying calcium- and calmodulin-dependent protein kinase IIα (CaMKIIα)–driven calmodulin-based genetically encoded GFP calcium indicators (GCaMP6s) and underwent optical fiber implantation into the posterior PVT at P49. Subsequently, observer mice were cohoused with demonstrator mice at P58 for an additional 2 weeks (Fig. 2, A to C).

We recorded the population Ca^2+^ activity of PVT glutamate neurons at P77 while the habituated observer engaged in sequential social interactions with control and BV-inflamed demonstrators. Upon aligning the photometric trace to each instance of body contact (brief but repeated whole or partial body contact), we observed a contact-related increase in population activity of PVT excitatory neurons in observer mice interacting with the painful demonstrator compared to the control demonstrator (Fig. 2, D and E, and movie S1). To quantify the Ca^2+^ changes, we used two parameters: mean *z*-score and the averaged area under curve (AUC; see Materials and Methods for details). Analysis of both mean *z*-score and AUC consistently indicated robust activation of PVT glutamate neurons during the emotional contagion of pain (Fig. 2, F and G).

To directly examine the causal role of PVT activity in contagious pain, we conducted chemogenetic silencing of PVT glutamate neurons. Specifically, we administered an AAV carrying a CaMKIIα-driven inhibitory designer receptor exclusively activated by designer drugs (DREADD) neuromodulator, namely AAV-CaMKIIα-hM4Di-mCherry, into the posterior PVT of AFR mice at P49. These mice were then cohoused with demonstrators at P58 for 2 weeks and subsequently subjected to the standard test for contagious pain, including handling, habituation, baseline pain test, social interaction, and post–pain test (Fig. 2H). Histological confirmation of correct viral infection was obtained, and the efficacy of virus-mediated neuronal inhibition was electrophysiologically validated (Fig. 2, I to L). Our results revealed that clozapine-*N-*oxide (CNO)–induced silencing of posterior PVT glutamate neurons impaired the social transfer of pain (Fig. 2, M to O) and emotion discrimination ability (Fig. 2, P to S), without any associated alterations in locomotion or anxiety levels (fig. S6). Chemogenetic inhibition of PVT glutamate neurons similarly abolished the occurrence of contagious pain in female observer mice (fig. S7).

Previous studies have demonstrated that PVT could be divided into two anatomically and functionally distinct subregions: anterior PVT and posterior PVT (*41*, *42*). The experimental data illustrated above were mainly based on manipulations on the posterior part of the PVT. To examine whether the anterior PVT also contributes to pain contagion, we chemogenetically silenced the neuronal activity of anterior PVT [anteroposterior to bregma (AP): −0.50 mm, medial to lateral (ML): +0.65 mm, and dorsal to ventral (DV): −3.65 mm; with a 10° angle toward the midline]. CNO-evoked neuronal inhibition had no effect on the development of contagious pain (fig. S8). These results indicate that posterior, but not anterior, PVT neurons contribute importantly to the emotional contagion of pain. Therefore, all subsequent experiments of this study were then focused on the posterior section of the PVT.

To compliment the chemogenetic data, we next performed optogenetic inhibition of the PVT glutamate neurons. Specifically, we microinjected AAV-CaMKIIα-eNpHR3.0-mCherry and implanted an optical fiber into the posterior PVT of AFR mice at P49. These mice were then cohoused with demonstrators for 2 weeks and underwent the contagious pain test (fig. S9, A to C). An 8-s on and 2-s off cycle of yellow light was delivered during the 30-min social interaction period (fig. S9A). We found that optogenetic inhibition of posterior PVT glutamate neurons equally inhibited the pain contagion, shown as an unaltered mechanical pain threshold when comparing post– versus pre–social interaction (fig. S9, D and E). A much less percentage of mice exhibited a significant decrease in pain threshold for the light on compared with the light off (fig. S9F). Here, optogenetic inactivation of posterior PVT neurons had no significant effect on baseline mechanical or thermal pain sensitivity (fig. S10).

Because *c-fos* mapping experiments implicated the PVN as another potential candidate mediating MS-induced behavioral deficits (fig. S5), coupled with substantial evidence supporting the role of oxytocin in empathic behaviors (*43*–*45*), we proceeded to investigate the involvement of PVN oxytocin neurons in the contagious pain using our behavioral paradigm. For this purpose, AAVs encoding Cre-dependent inhibitory DREADD (AAV-DIO-hM4Di-mCherry) were bilaterally injected into the PVN of oxytocin-Cre mice (fig. S11, A to C). Chemogenetic inactivation of PVN oxytocin neurons had no discernible effect on the contagious pain (fig. S11, D and E). An equivalent number of PVN-inhibited and control observers exhibited the contagious pain phenotype after interacting with the BV-inflamed demonstrator (fig. S11F). Collectively, these findings suggest that excitatory neurons in the PVT, rather than oxytocin neurons in the PVN, are essential for the social transfer of pain.

### MS-induced PVT inactivation causes deficits in the contagious pain

**Fig. 3.**
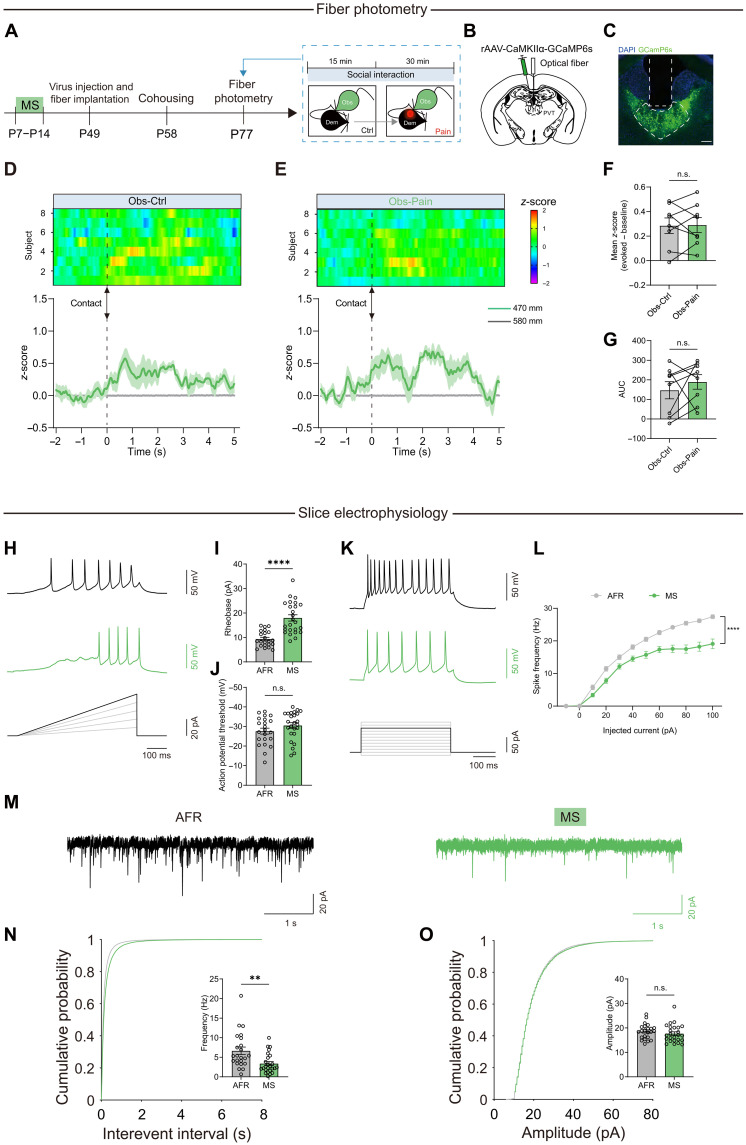
MS impairs activation of PVT neurons during pain contagion. (**A**) Schematic for fiber photometry recording of PVT neurons in the MS mice. (**B** and **C**) Schematic (B) and histological verification (C) of injection of AAV-CaMKIIα-GCaMP6s into the PVT and implantation of optical fiber into the PVT. Scale bar, 100 μm. (**D** and **E**) Heatmap (top) and peristimulus time histogram (bottom) of Ca^2+^ signals aligned with the onset of body contact between AFR observers and control (D) or painful demonstrators (E). (**F** and **G**) Quantification of the change in GCaMP6s signal as either mean *z*-score (F) or averaged AUC (G). *n* = 8 mice. (**H**) Representative traces of action potentials of PVT neurons recorded under a ramping current injection. (**I** and **J**) Action potential rheobase (I) and threshold (J) obtained from the PVT neurons of AFR and MS mice. *n* = 22 cells from six mice for AFR and *n* = 25 cells from six mice for MS. ****P < 0.0001; Welch’s *t* test. (**K**) Representative traces of action potentials in response to 80-pA current injections. (**L**) Action potential frequency in response to a range of current injections. *n* = 22 cells from six mice for AFR and *n* = 25 cells from six mice for MS. ****P < 0.0001; two-way RM ANOVA. (**M**) Representative traces of the spontaneous excitatory postsynaptic current (sEPSC). (**N**) Cumulative probability plots of the interevent interval and average frequency of sEPSCs. *n* = 23 cells from six mice for AFR and *n* = 25 cells from six mice for MS. **P < 0.01; Welch’s *t* test. (**O**) Cumulative probability plots and average amplitude of sEPSCs. Data are presented as means ± SEM.

Given the indispensable role of PVT glutamate neurons in contagious pain, we believe that MS might inhibit the activation of PVT neurons, leading to the inability to express the contagious pain. To test this hypothesis, we conducted fiber photometry recordings of PVT excitatory neurons in MS observer mice (Fig. 3, A to C). As anticipated, the PVT neurons of MS mice displayed no discernible alterations when aligning the Ca^2+^ signal trace with the onset of body contact events (Fig. 3, D and E, and movie S2). There were no notable differences in either the mean *z*-score or the AUC of the MS-insulted observers when interacting with control and painful demonstrators (Fig. 3, F and G). These findings are in line with the *c-fos* mapping data showing diminished tdTomato fluorescence in the PVT of MS-treated TRAP2:Ai9 mice relative to AFR control mice (fig. S5).

In an attempt to elucidate the potential mechanisms underlying the impaired PVT activation during observer-demonstrator interaction, we further probed for alterations in the intrinsic excitability of PVT glutamate neurons induced by MS. Ex vivo whole-cell patch-clamp recordings revealed that PVT neurons from MS adult mice (P77 to P84) exhibited marked hypoexcitability, as evidenced by an increased rheobase required to evoke the first action potential and markedly fewer spikes in response to suprathreshold depolarizing current injections (Fig. 3, H to L). Furthermore, the frequency of spontaneous excitatory postsynaptic currents (sEPSCs) was significantly reduced in MS neurons compared with AFR neurons, although the amplitude of sEPSCs did not differ between the two groups (Fig. 3, M to O). Of particular interest, when we performed the electrophysiological recordings immediately after early life MS, i.e., at P14, we failed to detect any abnormal changes in excitability or synaptic inputs of the PVT neurons (fig. S12), suggesting that MS-evoked deficits in PVT neurons develop gradually during the later stages of development. Together, these findings suggest that early life MS blunts the excitability and diminishes the excitatory inputs of PVT glutamate neurons.

**Fig. 4.**
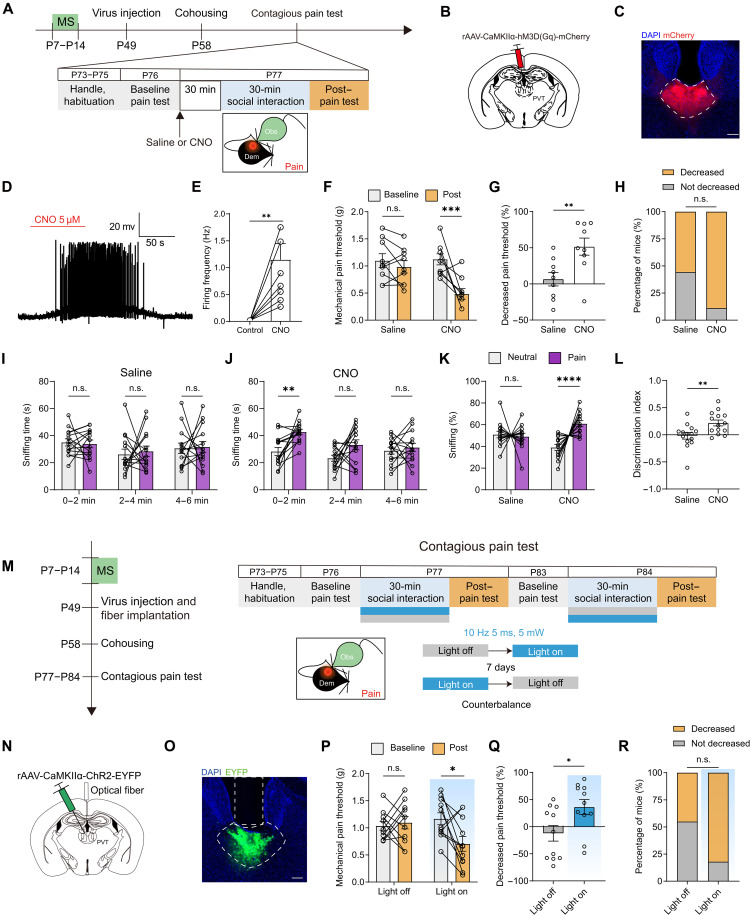
Chemogenetic or optogenetic activation of PVT rescues MS-induced contagious pain deficits. (**A**) Schematic timeline for (B) to (H). (**B** and **C**) Schematic (B) and histological verification (C) of injection of AAV-CaMKIIα-hM3D(Gq)-mCherry into the PVT. Scale bar, 100 μm. (**D** and **E**) Representative traces (D) and quantification (E) showing CNO (5 μM)–evoked increase in firing rate. *n* = 8 neurons from three animals. **P < 0.01; paired *t* test. (**F**) Changes in mechanical pain thresholds of MS observers. Saline: *n* = 9 mice; CNO: *n* = 9 mice. ***P < 0.001; two-way RM ANOVA. (**G**) Percentage decrease in mechanical pain threshold. **P < 0.01; unpaired *t* test. (**H**) Percentage of MS mice with or without contagious pain. (**I** and **J**) Sniffing time of saline (I) and CNO observers (J) toward neutral or painful demonstrators in 2-min bins. Saline: *n* = 14 mice; CNO: *n* = 14 mice. **P < 0.01; two-way RM ANOVA. (**K** and **L**) Percentage of sniffing time (K) and discrimination index (L) in the first 2 min of the emotional discrimination task. Saline: *n* = 14 mice; CNO: *n* = 14 mice. (K) ****P < 0.0001; generalized linear mixed model. (L) **P < 0.01; unpaired *t* test. (**M**) Schematic timeline for (N) to (R). (**N** and **O**) Schematic (N) and histological verification (O) of injection of AAV-CaMKIIα-ChR2-EYFP into the PVT and implantation of optical fiber into the PVT. Scale bar, 100 μm. (**P**) Changes in mechanical pain threshold of MS observers. Light off/light on: *n* = 11 mice. *P < 0.05; two-way RM ANOVA. (**Q**) Percentage decrease in mechanical pain threshold. *P < 0.05; unpaired *t* test. (**R**) Percentage of MS mice showing or not showing contagious pain. Data are presented as means ± SEM.

Given the essential role of PVT neuronal activity in contagious pain and the abolishment of PVT neuron activation during observer-demonstrator interaction induced by MS, we investigated whether selective activation of PVT glutamate neurons could rescue the impaired social transfer of pain in MS mice. To address this question, we administered AAVs carrying an excitatory DREADD, specifically AAV-CaMKIIα-hM3Dq, into the PVT of MS mice at P49 (Fig. 4, A to C). Functional validation through whole-cell patch-clamp recordings confirmed the efficacy of neural activation, as evidenced by CNO-induced facilitation of spontaneous firing of PVT neurons (Fig. 4, D and E). Behavioral assessments revealed that CNO administration, but not saline, rescued the contagious pain in MS observers, as indicated by a significant decrease in mechanical pain threshold and an increased percentage of mice exhibiting vicarious pain hypersensitivity (Fig. 4, F to H). Moreover, chemogenetic activation of the PVT glutamate neurons successfully rescued MS-evoked deficits in affective state discrimination (Fig. 4, I to L). However, the same manipulation could not further augment the socially transferred pain in the AFR control animals (fig. S13), suggesting that PVT glutamate neuron activity is not sufficient to enhance the expression of contagious pain.

To provide alternative evidence confirming the key role of PVT glutamate neurons in MS-induced impairment of contagious pain, we applied an optogenetic approach to elevate the activity of PVT neurons in MS mice. To this end, we first microinjected the AAV-CaMKIIα-ChR2-EYFP into the PVT and then implanted an optical fiber in the same brain area (Fig. 4, M to O). Delivery of blue light (10 Hz, 5 ms, and 5 mW) to the PVT during the observer-demonstrator interaction period could successfully rescue the impaired pain transfer (Fig. 4, P to R). Collectively, these findings indicate that postnatal MS detrimentally affects PVT glutamate neuron function, resulting in deficits in the pain contagion between familiar cagemates. Artificial elevation of PVT neuronal activity could rectify these MS-induced abnormalities and thereby restore the ability of MS mice to experience the social transfer of pain.

### Involvement of PVT → PrL projection in the contagious pain

To elucidate the circuit mechanisms underlying the involvement of PVT neurons in the contagious pain, we conducted anterograde tracing of behavior-recruited PVT neurons. Specifically, we administered AAV-DIO-mCherry into the PVT of TRAP2 mice at P49. One week post–virus infusion, observers were allowed to cohouse with demonstrators to establish a familiar relationship. At P70, 4-OHT was intraperitoneally injected 30 min before social interaction (90 min). The animals were then allowed to recover for at least 3 weeks to achieve selective and robust labeling of PVT neurons activated by social interaction and their projection fibers (fig. S14A). Whole-brain analysis of mCherry fluorescence revealed significantly more projections in the mPFC and NAc of PVT neurons recruited by painful demonstrators compared to those activated by control demonstrators (fig. S14, B and C). No significant group differences in projection density were observed in other regions such as the amygdala (Amg) and the bed nuclei of the stria terminalis (BNST) (fig. S14C).

**Fig. 5.**
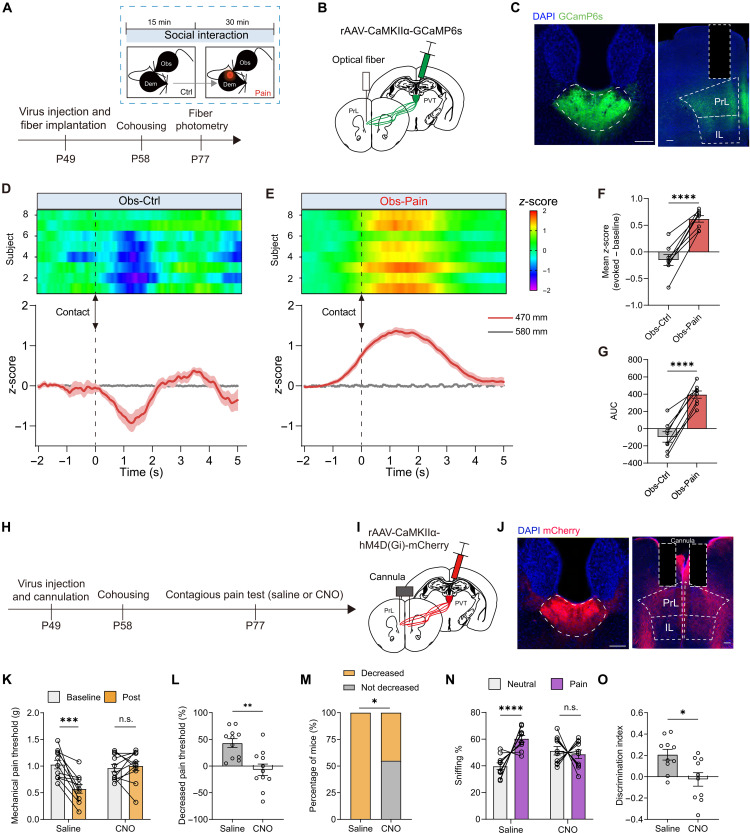
PVT → PrL projection mediates the pain contagion. (**A**) Schematic for fiber photometry recording of the PVT → PrL circuit in the AFR mice. (**B** and **C**) Schematic (B) and histological verification (C) of injection of AAV-CaMKIIα-GCaMP6s into the PVT and implantation of optical fiber into the PrL. Scale bars, 100 μm. (**D** and **E**) Heatmap (top) and peristimulus time histogram (bottom) of Ca^2+^ signals aligned with the onset of body contact between AFR observers and control (D) or painful demonstrators (E). (**F** and **G**) Quantification of the change in GCaMP6s signal as either mean *z*-score (F) or averaged AUC (G). *n* = 8 mice. ****P < 0.0001; paired *t* test. (**H**) Schematic timeline for (I) to (M). (**I** and **J**) Schematic (I) and histological verification (J) of injection of AAV-CaMKIIα-hM4D(Gi)-mCherry into the PVT and implantation of cannula into the PrL. Scale bars, 100 μm. (**K**) Changes in mechanical pain thresholds of AFR observers. *n* = 11 mice for each group. ***P < 0.001; two-way RM ANOVA. (**L**) Percentage decrease in the mechanical pain threshold. **P < 0.01; unpaired *t* test. (**M**) Percentage of AFR mice with or without contagious pain. *P < 0.05; Fisher’s exact test. (**N** and **O**) Percentage of sniffing time (N) and discrimination index (O) in the first 2 min of the emotional discrimination task. Saline: *n* = 10 mice; CNO: *n* = 10 mice. (N) ****P < 0.0001; generalized linear mixed model. (O) *P < 0.05; unpaired *t* test. Data are presented as means ± SEM.

Subsequently, we investigated the specific downstream circuitry responsible for PVT-mediated contagious pain. At first, fiber photometry recordings revealed elevated Ca^2+^ activity of the projection circuit from PVT to PrL of the mPFC, which precedes the body contact between observers and painful demonstrators (Fig. 5, A to G, and movie S3). Next, we directly inhibited the axonal terminals of PVT glutamate neurons by injecting AAV-CaMKIIα-hM4Di-mCherry into the PVT and implanting a drug cannula above the PrL (Fig. 5, H to J). Local infusion of CNO (3 μM and 0.2 μl per side) in the PrL significantly blocked the occurrence of contagious pain (Fig. 5, K to M). Consistently, chemogenetic silencing of PVT → PrL projections abolished the emotion recognition ability, shown as no significant difference in the time spent by the animal in sniffing neutral versus painful demonstrators (Fig. 5, N and O). In contrast, chemogenetic inhibition of the PVT → NAc circuit had no effect on the pain contagion (fig. S15). Therefore, these findings support the notion that the PVT → PrL circuit plays a more critical role in the social transfer of pain.

It has been previously reported that PVT neurons project to both PrL and infralimbic cortex (IL) of the mPFC (*42*, *46*). Thus, we next examined the relative contributions of these two PVT-innervated subregions to the contagious pain. First, we evaluated the innervation bias of PVT glutamate neurons in different subregions of the mPFC. We injected the anterograde monosynaptic virus, AAV1-hSyn-Cre, into the PVT of Ai9 mice (fig. S16A). By counting the number of tdTomato^+^ cells in PrL and IL, we found that PVT neurons mainly project to the PrL (64.5% of tdTomato^+^ cells) with relatively less innervation onto the IL (35.5% of tdTomato^+^ cells) (fig. S16, B and C). Then, we selectively manipulated the PVT projection terminals into IL to see the corresponding changes in the contagious pain (fig. S16, D to F). Comparisons of baseline and post–social interaction pain tests revealed a significant decrease in the mechanical pain threshold for both saline- and CNO-treated animals (fig. S16, G and H). Consistently, there was no significant difference in the percentage of mice exhibiting vicarious pain hypersensitivity (fig. S16I). These findings indicate that the PrL subregion predominantly receives glutamatergic innervation from PVT neurons and thus contributes importantly to the pain contagion.

### Chemogenetic activation of PVT → PrL projection reverses MS-evoked deficits in contagious pain

**Fig. 6.**
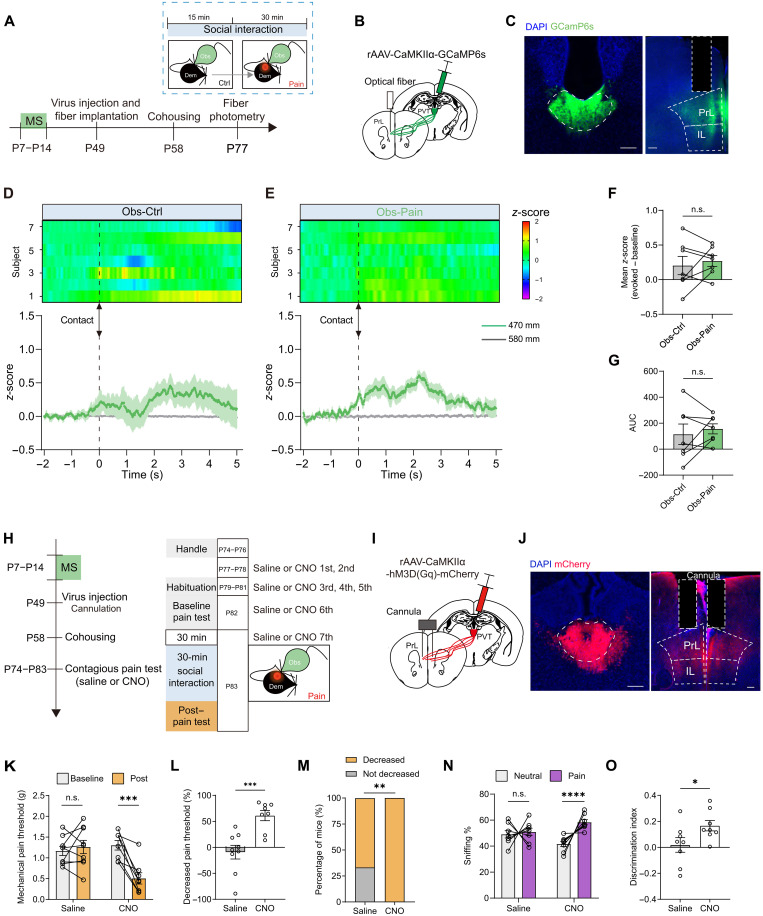
Chemogenetic activation of the PVT → PrL projection rescues MS-induced contagious pain deficits. (**A**) Schematic for fiber photometry recording of the PVT → PrL circuit in the MS mice. (**B** and **C**) Schematic (B) and histological verification (C) of injection of AAV-CaMKIIα-GCaMP6s into the PVT and implantation of optical fiber into the PrL. Scale bars, 100 μm. (**D** and **E**) Heatmap (top) and peristimulus time histogram (bottom) of Ca^2+^ signals aligned with the onset of body contact between AFR observers and control (D) or painful demonstrators (E). (**F** and **G**) Quantification of the change in GCaMP6s signal as either mean *z*-score (F) or averaged AUC (G). *n* = 7 mice. (**H**) Schematic timeline for rescuing MS-impaired contagious pain through repeated activation of the PVT → PrL projection. (**I** and **J**) Schematic (I) and histologic verification (J) of injection of AAV-CaMKIIα-hM3D(Gq)-mCherry into the PVT and implantation of cannula into the PrL. Scale bars, 100 μm. (**K**) Changes in mechanical pain thresholds of MS observers. Saline: *n* = 9 mice; CNO: *n* = 8 mice. ***P < 0.001; two-way RM ANOVA. (**L**) Percentage decrease in mechanical pain thresholds. ***P < 0.001; unpaired *t* test. (**M**) Percentage of MS mice with or without contagious pain. **P < 0.01; Fisher’s exact test. (**N** and **O**) Percentage of sniffing time (N) and discrimination index (O) in the first 2 min of the emotional discrimination task. Saline: *n* = 8 mice; CNO: *n* = 8 mice. (N) ****P < 0.0001; generalized linear mixed model. (O) *P < 0.05; unpaired *t* test. Data are presented as means ± SEM.

Building on the preceding results, we investigated whether activating the PVT → PrL circuit could normalize MS-induced impairments in contagious pain. Initially, fiber photometry recordings revealed a lack of significant activation of this projection in the observer mice subjected to repeated MS during postnatal development (Fig. 6, A to G). Notably, no discernible Ca^2+^ signal rises were detected when aligned with the onset of body contact behaviors for MS mice interacting with painful demonstrators. Subsequently, we selectively activated this projection in MS mice by expressing a stimulatory DREADD (AAV-CaMKIIα-hM3Dq-mCherry) in PVT neurons and implanting a drug cannula in the PrL (Fig. 6, H to J). Here, we locally administered CNO daily for 7 days before the behavioral test of contagious pain (Fig. 6H). Repeated chemogenetic activation of the PVT → PrL projection significantly reversed the deficits in emotion discrimination and contagious pain in MS mice, as evidenced by a decreased mechanical pain threshold, an increased percentage of mice exhibiting vicarious hyperalgesia, and an increased discrimination index in the affective state discrimination task (Fig. 6, K to O). Together, these findings suggest that PVT neurons mediate the contagious pain, at least, in part, through sending output to the PrL, and that attenuated activity within this circuit may constitute one crucial mechanism underlying MS-induced impairments in contagious pain.

### ST rescues MS-induced deficits in vicarious pain hypersensitivity through promoting activation of the PVT → PrL circuit

In addition to artificially restoring the contagious pain of MS mice using chemogenetic manipulations, we aimed to explore physiological means of rescue strategy by investigating the most critical component of maternal care lost during MS. Mammals exhibit a repertoire of robust and stereotypical maternal behaviors, such as licking, crouching, grooming, and retrieving, which have evolved to ensure the survival of the offspring (*47*, *48*). Almost all these mother-infant bonding behaviors involve a process of affective and pleasant ST, which is essential for the growth and well-being of the progeny (*31*). Previous studies have suggested that mimicking early life ST can mitigate the effects of MS on adult social behaviors (*49*). Therefore, we investigated whether ST-like tactile stimulation during the postnatal period could effectively rescue MS-induced defects in the vicarious pain hypersensitivity.

**Fig. 7.**
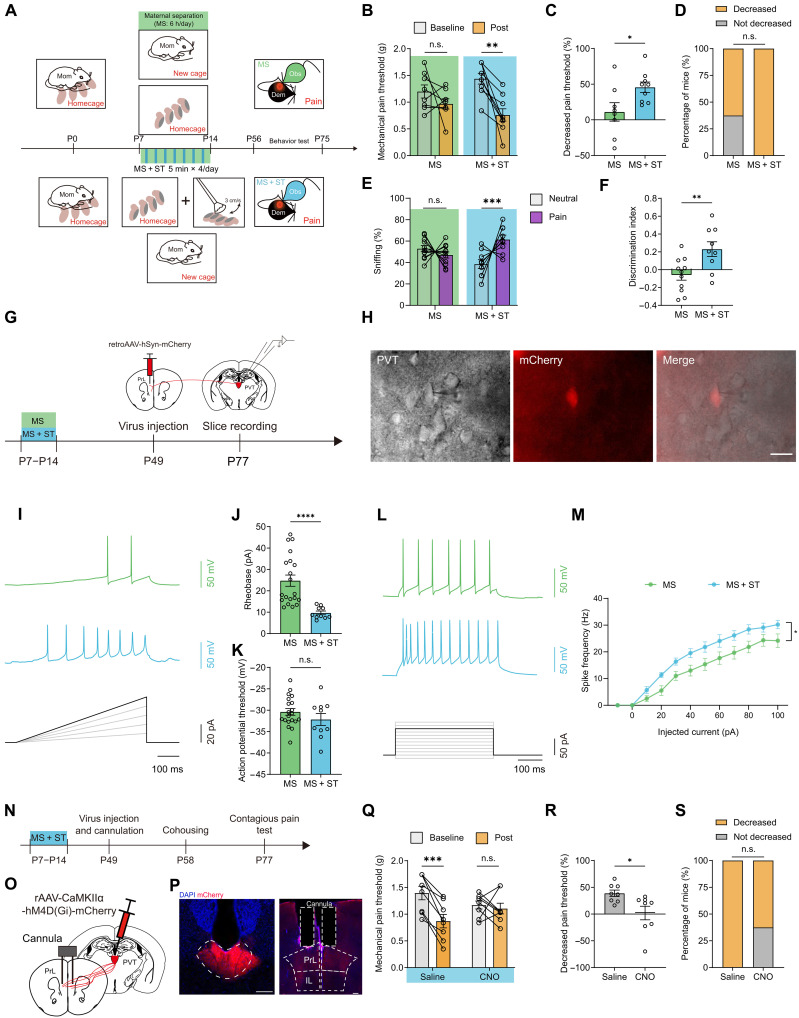
ST-like tactile stimulation rescues MS-induced contagious pain deficits through the PVT → PrL circuit. (**A**) Schematic timeline for (B) to (D). (**B**) Changes in mechanical pain thresholds of MS observers. MS: *n* = 8 mice; MS + ST: *n* = 9 mice. **P < 0.01; two-way RM ANOVA. (**C**) Percentage decrease in mechanical pain thresholds. *P < 0.05; unpaired *t* test. (**D**) Percentage of mice with or without contagious pain. (**E** and **F**) Percentage of sniffing time (E) and discrimination index (F). MS: *n* = 11 mice; MS + ST: *n* = 9 mice. (E) ***P < 0.001; generalized linear mixed model. (F) **P < 0.01; unpaired *t* test. (**G**) Schematic timeline for (H) to (M). (**H**) Representative images of real-time recording of one PrL-projecting PVT neuron (mCherry^+^). Scale bar, 10 μm. (**I** to **K**) Representative traces (I), action potential rheobase (J), and threshold (K). *n* = 20 cells from four mice for MS; *n* = 10 cells from three mice for MS + ST. ****P < 0.0001; unpaired *t* test. (**L**) Representative action potential traces in response to 80-pA current injections. (**M**) Action potential frequency in response to a range of current injections. *n* = 15 cells from four mice for MS; *n* = 11 cells from three mice for MS + ST. *P < 0.05; two-way RM ANOVA. (**N**) Schematic timeline for (O) to (S). (**O** and **P**) Schematic (O) and histological verification (P) of injection of AAV-CaMKIIα-hM4D(Gi)-mCherry into the PVT and implantation of cannula into the PrL. Scale bars, 100 μm. (**Q**) Changes in mechanical pain thresholds of MS + ST observers. *n* = 8 mice for each group. ***P < 0.001; two-way RM ANOVA. (**R**) Percentage decrease in mechanical pain thresholds. *P < 0.05; unpaired *t* test. (**S**) Percentage of MS + ST mice with or without contagious pain. Data are presented as means ± SEM.

To achieve this, we used a previously validated ST protocol known to promote affiliative social communication (*27*). Specifically, we applied gentle tactile stimulation to the backs of MS-treated mice at a stroking speed of 3 cm/s. The ST regime consisted of four rounds of stroke each day for 7 days (Fig. 7A). These four rounds of stroke were divided into two sessions: morning and afternoon sessions. Each half-day session included two rounds of stroke at 30-min intervals, with each round lasting for 5 min. At P56, we prepared the animals for cohousing, handling, and contagious pain tests. Our results revealed that ST-like tactile stimulation significantly restored the impaired social transfer of pain induced by MS, as evidenced by a decreased mechanical pain threshold (Fig. 7, B and C) and an increased percentage of observer mice exhibiting vicarious pain hypersensitivity (Fig. 7D). The MS-evoked deficits in emotional discrimination were also reversed by ST-like stimulation (Fig. 7, E and F). A similar rescue effect was observed in female mice subjected to MS + ST compared to MS alone (fig. S17). However, the application of the ST protocol failed to further enhance the pain contagion displayed by the AFR-treated mice (fig. S18). Overall, these results suggest that repetitive ST-like tactile stimuli could effectively rescue the impairment in vicarious pain and affective preference due to early life stress.

To explore the potential mechanisms underlying the therapeutic effects of ST, we conducted whole-cell patch-clamp recordings of the PrL-projecting PVT neurons in MS-treated animals, with or without receiving the ST paradigm (Fig. 7, G and H). Analysis of the action potential properties revealed that PVT neurons from the MS + ST group of mice exhibited hyperexcitability, as evidenced by increased spiking in response to depolarizing current injections and decreased rheobase compared with neurons from mice subjected to MS alone (Fig. 7, I to M). Consistent with the electrophysiological data, fiber photometry recordings demonstrated elevated body contact–associated Ca^2+^ signals of the PVT → PrL projection in the MS + ST mice compared to the MS-only mice (fig. S19 and movie S4).

Subsequently, we investigated whether inhibition of the PVT → PrL circuit could block the socially transferred pain in MS + ST mice. To test this hypothesis, we injected AAV-CaMKIIα-hM4Di-mCherry into the PVT, followed by the implantation of a drug cannula above the PrL (Fig. 7, N to P). Chemogenetic silencing of the PVT → PrL pathway by local infusion of CNO in the PrL reinstated the pain contagion in the MS-treated mice, whereas saline-injected control animals still exhibited the contagious pain (Fig. 7, Q to S). These findings suggest that sufficient activation of the PVT → PrL circuit in adulthood might, at least partially, contribute to the restored contagious pain in mice receiving ST-like tactile stimulation during the MS period.

## DISCUSSION

Emotional contagion, recognized as an essential aspect of affective empathy, involves perceiving and sharing the emotional states of others (*1*, *3*). The social transfer of pain serves as a validated animal model of emotional contagion, facilitating the study of molecular and circuit mechanisms underlying this behavior (*13*, *50*–*52*). However, fewer studies have explored how external environmental factors influence the emotional contagion of pain and its neurobiological underpinnings. Here, we demonstrate an impaired pain contagion in observers subjected to early life stress, such as MS. This deficit in social transfer of pain partly stems from the hypoactivity of PVT glutamate neurons and their projections to the PrL during the observer-demonstrator social interactions. Activation of the PVT → PrL circuit, either through chemogenetic interventions or through ST-like tactile stimulation, effectively mitigated the MS-evoked deficits in pain contagion (fig. S20).

A deeper understanding of the neural circuits underlying specific empathic responses will significantly advance the development of therapies targeting pathological empathic impairments in various neuropsychiatric disorders. The PVT, belonging to the midline and intralaminar group of thalamic nuclei, was initially proposed to serve as part of the thalamocortical arousal system (*53*). Recent investigations using sophisticated techniques for monitoring and manipulating neuronal activity have provided causal evidence linking the PVT to the regulation of numerous emotional and motivated behaviors (*25*, *38*). In this study, we identified the PVT as a central hub encoding and driving the social transfer of pain. While fiber photometry recordings revealed an elevated activity of PVT glutamate neurons during observer-demonstrator interactions, chemogenetic silencing of PVT activity diminished the contagious pain. PVT glutamate neurons were activated even before the body contact between observer and painful demonstrator. We can address this preceding activation pattern as follows. The observer may perceive demonstrators’ pain via visual (flinching or licking) (*11*, *33*) or olfactory (urine smell) (*34*, *50*) cues. Perception of the negative states of the demonstrator triggers an approach and body contact by the observer, which has been believed to maximize the information transfer and promote emotional contagion (*1*). Therefore, the activity of PVT neurons during observer-demonstrator interactions, as revealed in this study, may correlate with the extent of perceived negative emotions, thus exhibiting an early increase during approaching and reaching a peak after the body contact.

The present results also offered insights into how the PVT interacts with other brain regions to contribute to emotional contagion and other empathic processes. Our findings demonstrate that PVT glutamate neurons promote contagious pain via their projections to the mPFC. Given the essential roles of the PVT and mPFC in pain and emotion (*39*, *46*, *54*–*56*), it is reasonable to postulate that the PVT → mPFC pathway is intimately involved in the emotional contagion of pain. This study shows a crucial role of the PVT → PrL, but not PVT → IL, projection in the social transfer of pain. This discovery unveils a previously unrecognized aspect of functional diversity within PVT neurons and delineates an anatomically defined circuitry governing the occurrence of contagious pain. Consistently, prior studies have provided supportive evidence highlighting the roles of the PVT (*26*, *57*) and mPFC (*12*, *58*) in distinct forms of empathy-like behaviors.

Several MS paradigms involving periodic disruption of social bonding by separating pups from their mother have been developed to investigate the effects of early life stress on adult behaviors (*20*–*22*, *59*–*61*). In this study, we used a slightly modified version of the MS procedure, which resulted in significant impairments in the contagious pain and marked hypoexcitability of the PVT. This hypoactivity of the PVT is crucial for the loss of pain contagion observed in MS animals. Chemogenetic activation of PVT neurons or the PVT → PrL projection effectively reversed MS-induced deficits in vicarious pain hypersensitivity. We hypothesize that the decreased activity of PVT glutamate neurons in MS-treated observers during events of body contact compromises their ability to perceive inputs from the painful demonstrator and transmit this information to downstream targets, thereby impeding the pain contagion. Augmenting the activity of PVT neurons and this specific projection circuit could offer a promising therapeutic approach for apathetic children affected by early life stress. While PVT neurons are known to project to the NAc (*62*, *63*), this projection does not contribute to the social transfer of pain in the current behavioral paradigm. Further extensive investigations into PVT neuronal circuitry will enhance our understanding of how cell-type–specific and/or projection-specific maladaptations in PVT neural circuits lead to empathic impairments following early adverse experiences.

Notably, the present data from TRAP2:Ai9 mice also showed a significant activation of PVN neurons when the observer mice socially interact with the painful demonstrator for 90 min. Moreover, MS treatment suppresses this activation caused by contagious pain. Then it would be necessary to test whether PVN neurons equally contribute to MS-evoked contagious pain deficits. However, oxytocin neurons in the PVN, which have been widely believed to play a crucial role in prosocial behaviors (*43*–*45*), are dispensable for contagious pain in our animal model. Therefore, PVN oxytocin neurons are less likely involved in MS-evoked contagion pain impairment, although we cannot completely exclude the possible role of other cell types of the PVN.

ST serves as a prevalent form of prosocial comforting behavior, often providing a pleasant experience to others and commonly used in the context of consolation to alleviate stress in adult recipients (*4*, *5*, *64*). Moreover, specific mother-infant bonding behaviors, such as maternal licking and grooming, are widely acknowledged as some of the most important forms of early ST, deemed essential for the survival and positive development of offspring (*30*, *31*). In our study, we administered gentle ST-like stimulation to MS mice to simulate and compensate for the absence of maternal licking and grooming. The stimulation protocol was adapted from a previous study demonstrating enhanced social interaction by ST (*27*). We observed a notable restoration of MS-induced impairments in the pain contagion following repetitive ST-like tactile stimuli administered during the postnatal period. Furthermore, chemogenetic silencing of this circuit abrogates the beneficial effects of ST. These findings not only further underscore the pivotal role of PVT neurons and projections in the contagious pain but also strongly suggest that ST constitutes an indispensable aspect of maternal care. Adequate experiences of affiliative or pleasant touch in early life exert enduring positive effects on the behavioral performance of offspring.

In summary, our study has revealed a neural circuit mechanism contributing to the adulthood empathic abnormalities induced by early life stress. Our findings highlight the essential role of PVT glutamate neurons and their excitatory projections to the PrL in the establishment of contagious pain. MS exposure leads to hypoactivity within this PVT circuit, thus culminating in contagious pain deficits. This represents a significant advancement in our understanding of how adverse early life events can disrupt specific neural circuit activity and cause behavioral dysfunctions in adulthood. The elucidated circuit mechanisms underlying impaired emotional contagion may offer improved therapeutic strategies for treating empathic impairments stemming from adverse childhood experiences.

## MATERIALS AND METHODS

### Animals

Animal care and use strictly followed institutional guidelines and governmental regulations. We conducted experiments exactly as approved by the Animal Care and Use Committees (IACUC) at Shanghai Jiao Tong University (policy number: DLAS-MP-ANIM. 01–05). All mice were maintained under a 12-hour/12-hour day/night cycle at 22° to 25°C with free access to rodent food and water under environmentally controlled conditions. The animals used in the experiments were adult male (6 to 12 weeks old) C57BL/6J mice (Shanghai Slac Laboratory Animal Center, Shanghai), Fos-CreER^T2^ (TRAP2; the Jackson Laboratory, Bar Harbor, ME; stock no. 030323) (*37*), Ai9 (the Jackson Laboratory; stock no. 007909), and Oxt-Cre transgenic mice (provided by J. Hu at Shanghai Tech University). The TRAP2 mice were crossed with Ai9 Cre-reporter mice to visualize active neurons (*14*). All mice were group housed until surgery. All behavioral procedures were performed during the day cycle.

### Maternal separation

The MS paradigm was adopted from previously published methods with minor modifications (*19*, *21*, *60*). Briefly, pregnant female mice were individually housed at 16 to 18 days of gestation. From P7 to P14, MS dams were separated from their pups by placing them in other cages in another room for 6 hours. The separation cages remained similar to their home cages and were unchanged throughout the 7-day period. During cage changing, some old bedding materials were transferred into the new cage to limit novelty stress. During this separation period, MS pups were placed together in their home cages with normal bedding and maintained at 32° ± 1°C with a heating pad. The timing of the separation period was randomized but occurred within the light cycle (9:00 a.m. to 6:00 p.m.). At the end of the separation period, MS dams were reunited with the pups. Control pups remained undisturbed in the maternal nest. To investigate the time window during which MS could affect the social transfer of pain, we also subjected pups to MS at P2 to P7 or at P15 to P21. All pups were weaned at P21 and housed in groups until the beginning of the experiments.

### Viral constructs and stereotaxic surgery

The following AAVs were used: AAV2/9-CaMKIIα-GCaMP6s [serotype 2/9, titer: 5.47 × 10^12^ vector genomes (vg)/ml], AAV2/9-CaMKIIα-hM3D(Gq)-mCherry (serotype 2/9; titer: 5.32 × 10^12^ vg/ml), AAV2/9-CaMKIIα-hM4D(Gi)-mCherry (serotype 2/9; titer: 5.33 × 10^12^ vg/ml), rAAV2/9-CaMKIIα-eNpHR3.0-EYFP (serotype 2/9; titer: 5.25 × 10^12^ vg/ml), rAAV2/9-CaMKIIα-EYFP (serotype 2/9; titer: 5.31 × 10^12^ vg/ml), rAAV2/9-CaMKIIα-ChR2-EYFP (serotype 2/9; titer: 5.46 × 10^12^ vg/ml), AAV2/9-EF1α-DIO-mCherry (serotype 2/9; titer: 5.18 × 1012 vg/ml), AAV2/9-hSyn-DIO-hM4D(Gi)-mCherry (serotype 2/9; titer: 5.19 × 1012 vg/ml), AAV2-retro-hSyn-mCherry (serotype 2; titer: 5.32 × 1012 vg/ml), and AAV2/1-hSyn-Cre (serotype 2/9; titer: 5.75 × 10^12^ vg/ml). All viral vectors were purchased from BrainVTA Co. Ltd. (Wuhan, China) and stored in aliquots at −80°C until further use.

We performed stereotaxic surgery as previously described (*56*, *65*). Mice at 7 weeks old were anesthetized with 1 to 1.5% isoflurane and mounted in a stereotaxic frame (RWD Life Science, Shenzhen, China). Body temperature was kept stable using a heating pad. The skull was exposed with a small incision, and holes were drilled. Glass pipettes (tip diameter: 10 to 20 μm) were made with a P-97 Micropipette Puller (Sutter glass pipettes; Sutter Instrument Company, USA) for AAV microinjections. The microinjection pipettes were filled with silicone oil and then connected to a microinjector pump (RWD Life Science, Shenzhen, China) to achieve full air exclusion. AAV-containing solutions were loaded into the tips of pipettes and injected at the following coordinates: posterior PVT: AP (−1.40 mm), ML (+0.55 mm), and DV (−3.00 mm), with a 10° angle toward the midline; anterior PVT: AP (−0.50 mm), ML (+0.65 mm), and DV (−3.65 mm), with a 10° angle toward the midline; PVN: AP (−0.88 mm), ML (±0.20 mm), DV (−4.95 mm); and PrL: AP (+1.94 mm), ML (±0.30 mm), and DV (−2.7 mm). We injected virus-containing solutions bilaterally or unilaterally into the PVT (0.2 μl), PVN (0.3 μl per side), and PrL (0.2 μl per side), at a rate of 0.1 μl/min. After injection, the pipette remained in place for an additional 10 min to allow the injectant to diffuse adequately. For optogenetic manipulations, optical fibers [200-μm outer diameter (OD), numerical aperture (NA) = 0.37; Inper, Hangzhou, China] were implanted above the PVT at the following stereotaxic coordinates (AP: −1.40 mm, ML: 0.00 mm, and DV: −2.90 mm). Dental cement was applied to cover the exposed skull and to secure the implants. Mice were allowed to recover for at least 3 weeks before behavioral and other tests, and the injection or implantation sites were examined at the end of the experiment by classical histological procedures.

### ST-like stimulation

The ST paradigm was implemented following previously published protocols with slight modifications (*27*, *30*). From P7 to P14, half of the separated pups were designated as the MS + ST group, while the remainder constituted the MS group. These pups were relocated from their home cages to a confined space (10 cm by 5 cm by 3 cm) lined with cotton bedding. Using a powder brush (9.9 cm–by–5.5 cm soft fan-shaped brush), experimenters gently stroked the pups’ backs, moving from the neck to the lumbar enlargement region at a consistent speed of 3 cm/s and force (maximum of 25 mN) to elicit a pleasurable sensation. Stroking sessions consisted of four trials/day. Two trials were conducted in the morning (9 to 11 a.m.), and the other two in the afternoon (1 to 3 p.m.). Each trial of stroking lasted 5 min, with a 30-min interval between morning and afternoon trials. For the AFR + ST group, half of the pups underwent the same procedure as the MS + ST group, while the other half pups remained undisturbed in the AFR settings.

We also tried a different pattern of stroking, including increased speed, force, and reversed direction, to induce an “unpleasant touch stimulation” condition. However, pups exhibited agitation and attempted to escape from the brush within the confined space under this condition. Consequently, we established a control group for ST that did not receive any tactile stimulation as described above.

### Contagious pain test

The contagious pain test was conducted following established procedures (*32*, *33*). Before testing, observer mice were housed together with demonstrator mice, matched for sex and body weight (with a difference of less than 10%). After 2 weeks of cohabitation, both observers and demonstrators underwent handling by experimenters and were acclimatized to an acrylic test box (19 cm by 19 cm by 30 cm) containing the same bedding as their home cages. This acclimatization process occurred in pairs over a period of at least 3 days. Observers were additionally familiarized with the mechanical pain testing setup.

On the day of testing, each pair of observer and demonstrator mice was placed in the test boxes for a duration of 15 min. Subsequently, the painful demonstrators were gently removed from the test box, lightly restrained, and administered a subcutaneous injection of 25 μl of BV (0.4% lyophilized whole venom of *Apis mellifera* dissolved in physiological saline) into the plantar surface of the left hind paw. Control demonstrators received an equal volume of normal saline injection. Following the injection, the BV-inflamed or saline-injected demonstrators were introduced to the test box and allowed to freely interact with their paired observers for 30 min. Immediately after the social interaction, the observers were transferred to the behavioral test room and subjected to pain sensitivity measurement.

### Mechanical pain threshold measurement

The mechanical pain test setup comprised a metal mesh with pores measuring 0.5 cm by 0.5 cm and a small nontransparent acrylic box (10.5 cm by 10.5 cm by 5 cm). Following a 3-day habituation period, observers underwent baseline mechanical pain threshold testing. Behavioral responses (withdrawal, shaking, or paw licking) to mechanical stimulation (applied for 5 s) of the central plantar surface of the left hind paw were assessed with the von Frey filaments (ranging from 0.01 to 2 g; touch test) using the up-down method (*66*).

After the 30-min social interaction period, observers were transferred to the test setting and allowed to acclimatize for 10 to 20 min before undergoing the mechanical pain test. The percentage decrease in mechanical pain threshold was determined as [1 − (post–social interaction pain threshold/baseline threshold)] × 100%. For the time course experiment, tests were conducted at 0, 2, 4, 8, and 24 hours following the social interaction.

### Hot plate test

To measure the baseline thermal pain sensitivity, we adopted the well-established hot plate test as described previously (*30*, *67*, *68*). Briefly, the animal was placed on a hot plate (Ugo Basile, 35150), and the latencies to hindpaw flinching, lifting, or licking were measured. The hot plate was set at 50°, 52°, or 56°C, and all animals were tested with a minimum of 5-min intervals between sequential tests. To avoid tissue injury, a cutoff time was set at 60 and 30 s for assays at 50° to 52°C and 56°C, respectively.

### Capsaicin-induced chemical pain

To examine the changes in chemical pain sensitivity after early life MS or PVT inhibition, we adopted the capsaicin-induced acute pain model (*56*, *69*). Briefly, capsaicin (0.2 μg/μl, 25 μl, dissolved in saline with 7% Tween 80) was injected subcutaneously into the plantar surface of the right hindpaw, and the duration of nocifensive behaviors, including licking or flinching, was measured for 5 min.

### Emotional discrimination task

The emotional discrimination task was adapted from previous studies (*35*, *70*). Initially, observers underwent a habituation period lasting 3 days, during which they spent 10 min in a standard mouse cage (32 cm by 21 cm by 17 cm) containing one dark separator (11 cm by 17 cm) positioned between two cylindrical wire cups (10.5 cm in height and 10.2 cm in bottom diameter). A plastic cup was placed atop the wire cups to prevent the observers from climbing. Observers, neutral demonstrators, and painful demonstrators were housed in separate cages during this period. On the test day, observers were introduced into the cage 15 min before the start of the test. The painful demonstrators received an intraplantar injection of BV solution 5 min before the test (Fig. 1H). Subsequently, both neutral demonstrators and painful demonstrators were simultaneously placed into the wire cups. Observers were then given 6 min to explore the cage. Video recordings from digital cameras positioned overhead were analyzed using the behavior tracking system (EthoVision X15, Noldus).

Sniffing behaviors of the observers were scored for each 2-min segment, defined as instances where the distance between the observer’s nose and the wire cups was less than 2 cm. The discrimination index was calculated using the formula: [(sniffing time spent on painful demonstrator – sniffing time spent on neutral demonstrator)/total sniffing time]. The percentage of sniffing was determined by dividing the sniffing time spent on either the painful or neutral demonstrator by the total sniffing time. In line with previous literature (*34*, *35*), observers exhibited more pronounced discrimination during the first 2 min. Hence, unless stated otherwise, the sniffing percentage and discrimination index used data from the initial 2 min.

### Elevated plus maze

The elevated plus maze comprised two open arms, two closed arms, and a central platform elevated 30.5 cm above the ground. Mice were positioned in the center of the maze with their heads facing the open arms and allowed to explore for 5 min. The movement of the test mice was recorded using an overhead-mounted video camera. The percentage of time spent in the open and closed arms, as well as the total number of entries into the open arms, was analyzed using the behavior tracking system (EthoVision X15, Noldus).

### Open field test

Each mouse was individually placed in the open field arena measuring 44 cm by 44 cm by 44 cm and allowed to move freely for 10 min. The open field area was divided into three distinct zones: a central area measuring 20 cm by 20 cm, four corners measuring 10 cm by 10 cm each, and the periphery. The movement of the mice was monitored using a webcam. Subsequently, the percentage of time spent in the center or corner zones and the total distance traveled within the arena were analyzed using the behavior tracking system (EthoVision X15, Noldus).

### Three-chamber test

To evaluate the sociability and social novelty of the subjects, we used the three-chamber behavioral test as described previously (*71*, *72*). A nontransparent acrylic box measuring 68 cm by 22 cm by 24 cm, with two partitions creating two side chambers (left and right; each 28 cm by 22 cm by 24 cm), separated by a central chamber (12 cm by 22 cm by 24 cm), was used. During the initial 10-min session, the test mouse was placed in the middle of the three-chamber apparatus to habituate to its surroundings, where two empty wire cages (10.5 cm in height and 10.2 cm in bottom diameter) were positioned in the left and right chambers. In the subsequent 10-min session, an age- and gender-matched unfamiliar mouse was introduced into one of the wire cages, designated as the “stranger 1” compartment, while the other side contained an empty wire cage. For the third 10-min session, another age- and gender-matched unfamiliar mouse was placed in the previously empty wire cage, which then became the “stranger 2” side. The designation of the different sides was randomly assigned in a counterbalanced manner.

Mouse movement was monitored using a webcam and analyzed by a behavior tracking system (EnthoVision X15, Noldus). The duration of time spent exploring within a 2-cm radius of each wire cage (sniffing time) was recorded. The sociability index was calculated as [(sniffing time with stranger 1 – sniffing time with empty wire cage)/(sniffing time with stranger 1 + sniffing time with empty wire cage)], while the social novelty index was calculated as [(sniffing time with stranger 2 – sniffing time with stranger 1)/(sniffing time with stranger 2 + sniffing time with stranger 1)].

### Activity-dependent neuronal labeling and anterograde tracing

Neuronal labeling via TRAP2 mice was conducted following previously described methods (*37*, *56*). Recombination was triggered using 4-OHT (Sigma-Aldrich, catalog no. H6278). Briefly, 4-OHT was dissolved at a concentration of 20 mg/ml in ethanol by shaking at 37°C for 30 min and then aliquoted and stored at −20°C for several weeks. Before usage, 4-OHT was redissolved in ethanol by agitation at 37°C for 30 min. Corn oil (Sigma-Aldrich, catalog no. C8267) was added to achieve a final concentration of 4-OHT (10 mg/ml), and ethanol was removed by vacuum evaporation under centrifugation. The resultant 4-OHT solutions (10 mg/ml) were stored for less than 24 hours at 4°C before use. All injections were administered intraperitoneally.

Mice were transferred from the vivarium to an adjacent holding room at least 3 hours before 4-OHT injections to minimize transportation-induced immediate early gene activity. Activity-dependent neuronal labeling was induced by a single intraperitoneal injection of 4-OHT (20 mg/kg) administered to the observers 30 min before social interaction with the demonstrators. Subsequently, mice were returned to the vivarium and maintained under a regular 12-hour light-dark cycle for the duration of the experiment. To determine the overall activation of brain regions by pain contagion in both AFR and MS mice, we crossed the TRAP2 mouse line with a Cre-dependent Ai9 reporter mouse line to achieve activity-dependent labeling of all activated neurons with tdTomato fluorescence (fig. S5). For anterograde tracing of contagious pain–activated PVT neurons, the AAV-DIO-mCherry was injected into the PVT of the TRAP2 mice. Then, a similar procedure was performed to label the PVT neurons activated during the observer-demonstrator interactions, and whole-brain analysis of the mCherry fluorescence was done to detect any differences in the projection fiber intensity between control and contagious pain groups (fig. S14). For anterograde tracing of the PVT → mPFC projection, AAV1-hsyn-Cre was microinjected into the PVT of Ai9 mice, and then histological analysis was performed on different slices of the PrL and IL subregions of the mPFC (fig. S16).

### Fiber photometry

Fiber photometry experiments were conducted using a two-color fiber photometry system (ThinkerTech, Nanjing, China), following previously described protocols with minor modifications (*65*, *73*, *74*). Fluorescent signals generated by a 470-nm laser beam (OBIS 488LS, Coherent) were directed by a dichroic mirror (MD498, Thorlabs), focused through a 10× objective lens (NA = 0.3; Olympus), and transmitted via a rotary joint. A 580-nm laser served as the internal control. An optical fiber (200-μm OD, NA = 0.37, Inper, Hangzhou, China) was surgically implanted into the PVT or PrL of the observer mice, positioned 0.1 mm above the virus injection site. An optical fiber guided the light between the rotary joint and the implanted optical fiber. The laser power was adjusted at the tip of the optical fiber to 40 to 60 μW to minimize bleaching of the GCaMP6s probes. The excited fluorescent signals were passed through a bandpass filter (MF525-39, Thorlabs) and collected in a photomultiplier tube (R3896, Hamamatsu). An amplifier (C7319, Hamamatsu) was used to convert the current output of the photomultiplier tube to voltage signals, which were then filtered through a low-pass filter (40-Hz cutoff; Brownlee 440). Analog voltage signals were digitized at 100 Hz using a Power 1401 digitizer and Spike2 software (Cambridge Electronic Design, Cambridge, UK).

Before the test, observer and demonstrator pairs underwent a habituation period in the test box lasting for 3 days. On the test day, the observer interacted first with a control demonstrator (intraplantar saline injection; no pain) within the test box, during which Ca^2+^ signals were recorded for 15 min. Subsequently, the observer engaged with the same demonstrator but now experiencing persistent inflammatory pain induced by intraplantar BV injection, with simultaneous recording of calcium signals and top-view behavioral video capturing for 30 min. The time when the observer’s head or nose contacted the demonstrator’s body was manually marked offline by experimenters who were blind to the animal group. For fiber photometry data analysis, contact was defined as any events of the observer’s head/nose touching any body part of the demonstrators.

Photometry data were exported to MATLAB mat files from Spike2 for further analysis. Initially, photometry datasets underwent baseline decay correction using the airPLS method (*75*). All the fiber photometry data were segmented and aligned to the onset of body contact events between observers and demonstrators. Subsequently, the *z*-score was calculated from the detrended signal *F* as *z*-score = [*F* – mean(*F*_0_)]/SD(*F*_0_). To compare the *z*-score during each episode of body contact, we analyzed the data for each 7-s time window around the behavioral event (2 s before and 5 s after). To align *z*-score signals with the behavior, all fiber photometry data were segmented on the basis of behavior events within individual trials and averaged first across different trials in one animal and then across different animals for each group. The *z*-score values were illustrated as either heatmaps or peristimulus time histograms, with shaded areas indicating the SEM. To statistically quantify the change in the fluorescent values, the mean *z*-score (evoked – baseline) and the AUC values were calculated from the same set of data. For computing the AUC values, we used the trapz function in MATLAB. It computes the approximate integral (AUC) using the trapezoidal rule, by dividing it into small trapezoids and summing their areas. For a function *y* = *f*(*x*) with data points (*x_i_y_i_*), this function divides the curve into segments between consecutive points and then treats each segment as a trapezoid (linear approximation). Last, it sums the areas of all trapezoids. Here is the formula

$$\text{AUC}=\sum_{i=1}^{n-1}\left(x_{i+1}-x_{i}\right)\times \frac{f\left(x_{i}\right)+f\left(x_{i+1}\right)}{2}$$

### Chemogenetic manipulations and cannula implantation

For the chemogenetic manipulations of neuron activity in the observers, we used AAVs encoding excitatory DREADD (hM3Dq), inhibitory DREADD (hM4Di), or mCherry. To manipulate local neuron activity in the PVT, either saline or CNO (2 mg/kg; Enzo, BML-NS105-0025; stored at −20°C and dissolved in 0.9% sterile saline to a concentration of 0.25 mg/ml before use) was administered intraperitoneally 30 min before the behavioral test. To manipulate the projection fibers of the PVT in the mPFC or NAc, cannulation was performed immediately after AAV microinjection into the PVT. Stainless steel guide cannulas (26 gauge; RWD Life Science, Shenzhen, China) were implanted into the PrL subregion of the mPFC (AP: +1.94 mm, ML: ±0.30 mm, and DV: −2.2 mm), IL subregion of the mPFC (AP: +1.70 mm, ML: ±0.30 mm, and DV: −3.0 mm), or NAc (AP: +1.10 mm, ML: ±0.55 mm, and DV: −4.45 mm) and secured to the skull with dental cement and skull screws. After surgery, a stainless steel obturator was inserted, and a cap was screwed on to prevent occlusion of the guide cannula.

During the habituation period, observers were briefly head restrained while the obturator was removed, and an injection tube (30 gauge; RWD Life Science) was inserted into the guide cannula. The injection tube extended 0.5 mm from the tip of the guide cannula. On the test day, saline or CNO (3 μM and 0.2 μl per side) was microinjected via the injection tube at a slow rate (200 nl/min). Following injection, the injection tube remained in place for 5 min to minimize drug backflow, after which the obturator was reinserted into the guide cannula. Thirty minutes post–saline or post–CNO microinjection, observers engaged in social interaction with painful demonstrators as described in the contagious pain test or engaged in the emotional discrimination task as described above. For the repetitive activation of PVT → PrL projection in MS mice, the first saline or CNO microinjection occurred on P77 and was repeated daily for seven consecutive days. On the seventh day (P83), the behavioral test was conducted 30 min after microinjection.

### Optogenetic manipulations

Optogenetic manipulations were performed according to the procedures published previously (*56*, *74*). On the day of the behavioral test (3 to 4 weeks after viral injection), mice were introduced to a fresh cage. The implanted optical fiber was connected to a 473-nm or a 589-nm laser power source (Changchun New Industries Optoelectronics Technology Co. Ltd., China) using a ceramic ferrule. The external optical fiber was attached to a rotary joint (FRJ_1x1_FC-FC, Doric Lenses) to allow the animal to freely behave. The test animal was allowed to habituate in the cage for 15 min with the external fiber attached. The onset, duration, and frequency of light pulses were controlled through a customized MATLAB program. The final output power was adjusted with an optical power meter (Sanwa) to reach 5 to 10 mW at the end of the implanted fiber tip. For optogenetic inhibition of PVT neurons during the contagious pain test, the yellow light pulses (589 nm, 10 mW, and constant) were delivered for 30 min with repeated cycles of 8-s on/2-s off patterns. For optogenetic inhibition of PVT neurons during the baseline mechanical or thermal pain test, the yellow light pulses were delivered continuously for the whole behavioral test. For optogenetic activation of PVT neurons during the contagious pain test, the blue light pulses (473 nm, 5 mW, 5 ms, and 10 Hz) were applied for 30 min. The location of the fibers was always examined at the end of the experiments, and data obtained from mice where the fibers were located outside the desired site were excluded from further analysis.

### Slice electrophysiology

Whole-cell recordings were obtained from PVT neurons in acute brain slices from AFR or MS mice, which had been stereotaxically injected with AAVs, as indicated in different figures. Electrophysiological recordings followed procedures outlined in previous publications concerning the PVT area (*42*, *76*, *77*). Specifically, mice were anesthetized with isoflurane and promptly perfused with ice-cold *N*-methyl-d-glucamine (NMDG) artificial cerebrospinal fluid (ACSF) containing 93 mM NMDG, 93 mM HCl, 2.5 mM KCl, 1.25 mM NaH_2_PO_4_, 10 mM MgSO_4_, 30 mM NaHCO_3_, 0.5 mM CaCl_2_, 25 mM glucose, 20 mM Hepes, 5 mM sodium ascorbate, 3 mM sodium pyruvate, and 2 mM thiourea (pH 7.4 and 295 to 305 mosmol). The brain was rapidly removed from the skull, and coronal sections of the PVT (280 μm) were cut using an automated vibratome (VT1200 S, Leica Microsystems). Brain slices containing the PVT area were then incubated in oxygenated NMDG ACSF at 32°C for 10 to 15 min before being transferred to normal oxygenated ACSF (126 mM NaCl, 2.5 mM KCl, 1.25 mM NaH_2_PO_4_, 2 mM MgSO_4_, 10 mM glucose, 26 mM NaHCO_3_, and 2 mM CaCl_2_) at room temperature for 1 hour.

A slice was subsequently transferred to the recording chamber, which was submerged and superfused with ACSF at a rate of 3 ml/min at 28°C. Cells were visualized under a fluorescence microscope (BX51WI, Olympus, Japan) equipped with differential interference contrast optics. Whole-cell patch-clamp recordings of PVT neurons were conducted using a MultiClamp 700B amplifier and Digidata 1440A interface (Molecular Devices). Patch electrodes (3 to 5 megohms) were backfilled with an internal solution containing 130 mM K-gluconate, 8 mM NaCl, 10 mM Hepes, 1 mM EGTA, 2 mM MgCl_2_, 2 mM adenosine 5′-triphosphate, and 0.2 mM guanosine 5′-triphosphate. Series resistance was monitored throughout the experiments. All data were filtered at 2 kHz, digitized at 10 kHz, and collected using pClamp10 software (Molecular Devices).

To validate the functional efficacy of chemogenetic manipulations, membrane potential was recorded under current clamp configuration, with a 1-min bath application of CNO (5 μM) and a 5-min washout period. The frequency of spontaneous action potential firing was used to validate the efficacy of hM3Dq, while membrane potential was used for hM4Di. To probe any differences in excitability of PVT neurons between AFR and MS or between MS and MS + ST groups, the action potentials were recorded under the current clamp mode by injecting a series of current pulses (400-ms duration and 0- to 100-pA intensity with an increment of 10 pA). The action potential threshold and rheobase were obtained as previously described (*56*). To test any changes in excitatory synaptic inputs, spontaneous sEPSCs were recorded under the voltage clamp mode at −70 mV. Offline electrophysiological data analysis was performed with Clampfit 10.6 (Molecular Devices) and the Mini Analysis Program (Synaptosoft).

### Histology and fluorescent immunostaining

Mice were deeply anesthetized with 1% sodium pentobarbital and were subsequently transcardially perfused with saline, followed by ice-cold 4% paraformaldehyde in 0.1 M phosphate-buffered saline (PBS). Following decapitation, the brain was swiftly extracted and postfixed overnight at 4°C in 4% paraformaldehyde. For checking the viral infection efficiency, coronal brain slices containing the entire PVT, with a thickness of 40 μm, were obtained using a vibratome (VT1000 S, Leica Microsystems, Japan). Subsequently, these slices were immersed in a 4′,6-diamidino-2-phenylindole (DAPI) solution (1:2000; Thermo Fisher Scientific, #D1306) for 15 min, followed by three washes in PBS with 0.1% Tween 20 (PBST), each lasting 15 min. The sections were then mounted onto glass slides with coverslips using the mounting medium.

For the assessment of viral expression within the mPFC or NAc, the entire mPFC and NAc were also sectioned. These slices underwent blocking with permeable buffer (0.3% Triton X-100 in PBS) containing 10% donkey serum for 2 hours at room temperature. Subsequently, they were incubated overnight at 4°C with primary antibodies [chicken anti–green fluorescent protein (1:500), Thermo Fisher Scientific, #A10262; rabbit anti-DsRed (1:500), Clontech, #632496] in permeable buffer containing 2% donkey serum. Afterward, the slices were washed four times with PBST for 15 min each and then incubated with secondary antibodies [goat anti-chicken Alexa Fluor 488 (1:200), Thermo Fisher Scientific, #A-11039; or donkey anti-rabbit Alexa Fluor 568, Thermo Fisher Scientific, #A10042] along with DAPI (1:2000) in PBS for 2 hours at room temperature. Following four washes in PBST, the slices were mounted onto glass slides using the mounting medium.

To verify the specificity of viral expression within PVN oxytocin neurons, the entire PVN was sectioned and incubated with mouse anti-oxytocin primary antibody (1:1000; Millipore, #MAB5296) for 48 hours at 4°C. Subsequently, it was treated with donkey anti-mouse secondary antibody (1:200; Alexa Fluor 488, Thermo Fisher Scientific, #A-21202).

The fluorescent signals were captured using confocal microscopes (Digital Eclipse A1R+, Nikon, Japan). Quantification of immunofluorescent labeling was performed using QuPath software (*78*), where fluorescence-positive cells and projection fibers were manually counted. The Aligning Big Brains & Atlases plugin was used to align brain atlases to slices. To assess the density of PVT projections, a boxed area (200 μm by 200 μm for mPFC, 260 μm by 260 μm for NAc, 200 μm by 200 μm for BNST, and 160 μm by 160 μm for Amg) containing fibers was selected in each region. The average pixel intensity (*F*_raw_) was calculated using QuPath. Similarly, a boxed area of the same size in a region devoid of fiber terminals was chosen to determine background intensity (*F*_background_). The signal intensity (*F*_signal_) was then computed as *F*_raw_ – *F*_background_. For each animal, *F*_signal_ was normalized by the maximum *F*_signal_ across all analyzed regions. The normalized *F*_signal_ was used to calculate the average terminal field intensity across animals. In addition, the locations of optical fiber and cannula were validated for all experiments using standard histological methods and confocal microscopy.

### Statistical analysis

No statistical methods were used to predetermine sample sizes. Any animals or data points were excluded from analyses if the sites of virus injection, drug infusion, or optic fiber implants were not accurately validated. Experiments were randomized where possible. The experimenters were not blinded to group allocation. Statistical analyses were carried out using GraphPad Prism 10.4 or MATLAB 2022b programs. The data were analyzed using unpaired Student’s *t* test with or without Welch’s correction, paired Student’s *t* test, Fisher’s exact test, generalized linear model, two-way analysis of variance (ANOVA), or two-way repeated-measures ANOVA with Dunnett’s or Šidák’s post hoc multiple-comparisons test. In this study, “*n*” denotes the number of mice or cells. All data are presented as mean ± SEM, with a significance threshold set at *P* < 0.05. Detailed information regarding sample sizes, types of statistical tests, *P* values, and test statistics can be found in table S1.
