# Supplementary material for: Dysfunction of the paraventricular thalamus–prelimbic cortex circuit underlies maternal separation–induced deficits in contagious pain
Source: Sci Adv. 2025 Oct 8;11(41):eady1944. doi: 10.1126/sciadv.ady1944 (PMC12506963; doi:10.1126/sciadv.ady1944)
Supplement: Supplementary file 1 — Figs. S1 to S20 Legends for movies S1 to S4 Legend for table S1 [file sciadv.ady1944_sm.pdf]

Science Advances
AAAS

Supplementary Materials for

Dysfunction of the paraventricular thalamus–prelimbic cortex circuit underlies maternal separation–induced deficits in contagious pain

Zichen Zhang *et al.*

Corresponding author: Tian-Le Xu, xu-happiness@shsmu.edu.cn; Ming-Gang Liu, liuminggang@ojlab.ac.cn; Fan Jiang, fanjiang@shsmu.edu.cn

Sci. Adv. **11**, eady1944 (2025)
DOI: 10.1126/sciadv.ady1944

The PDF file includes:

Figs. S1 to S20
Legends for movies S1 to S4
Legend for table S1

Other Supplementary Material for this manuscript includes the following:

Movies S1 to S4
Table S1

Supplementary Figure legends

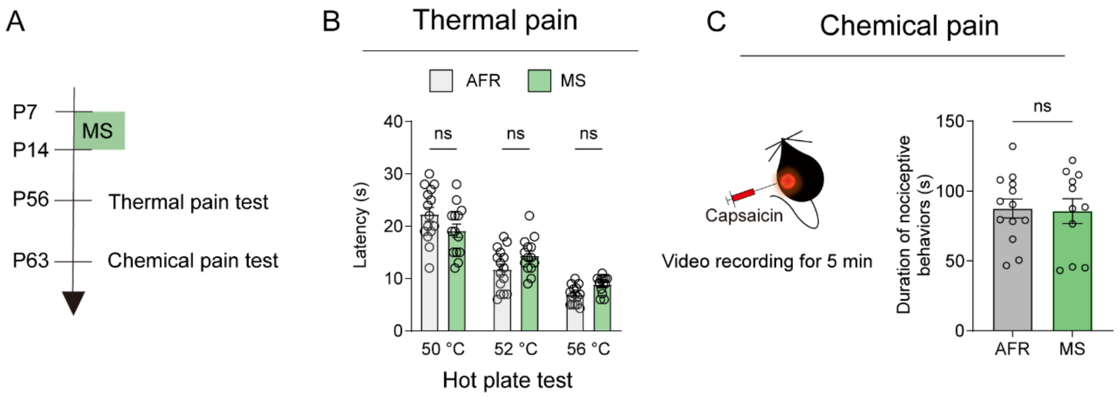

Fig. S1. Early life MS does not affect baseline pain sensitivity in adult male mice.
(A) Schematic of the timeline for assessing the effect of early life (P7-P14) MS on thermal and chemical pain sensitivity in the adulthood.
(B) No significant differences in the latencies to flinch or lick the hindpaw in hot plate tests. AFR: n = 15 mice; MS: n = 14 mice. ns, no significant difference, two-way RM ANOVA.
(C) No significant differences in the duration of nociceptive behaviors in the capsaicin test. AFR: n = 13 mice; MS: n = 11 mice. ns, no significant difference, unpaired t test.
Data are presented as means ± SEM. Further details of the statistical analysis are available in Table S1.

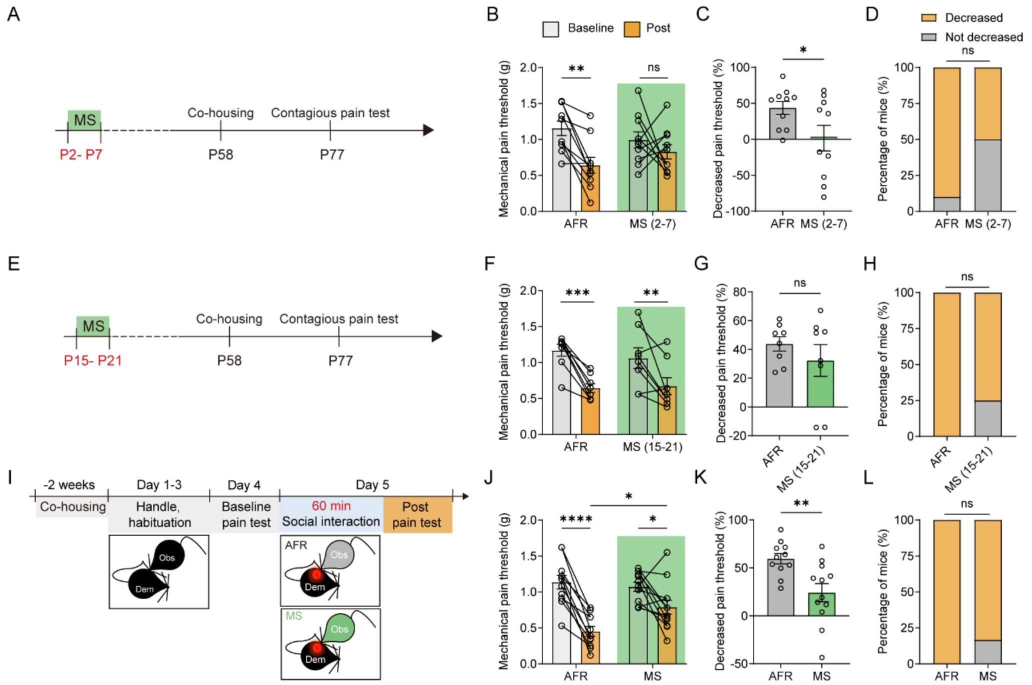

Fig. S2. A critical time window exists for MS to induce contagious pain deficits in adulthood.
(A) Schematic of the timeline for assessing the effect of early life (P2-P7) MS on social transfer of pain in the adulthood. The contagious pain test was started at P58 based on

the protocol shown in Figure 1A.

(B) Mechanical pain threshold of AFR and MS mice tested at both baseline and after 30 mins social interaction with the painful demonstrator. n = 10 mice for each group. $**p < 0.01$, Two-way RM ANOVA.

(C) The percentage of decrease in mechanical pain threshold. $*p < 0.05$, unpaired t test.

(D) Percentage of AFR and MS mice with or without a decrease in pain threshold.

(E to H) Similar to (A to D) but for animals subjecting to MS from P15 till P21. n = 8 mice for each group. $**p < 0.01$, $***p < 0.001$, two-way RM ANOVA.

(I) Schematic of the timeline for evaluating the effect of MS (P7-P14) on contagious pain test with 60 mins observer-demonstrator social interaction.

(J) Mechanical pain threshold of AFR and MS mice tested at both baseline and after 60 mins social interaction with the painful demonstrator. AFR: n = 11 mice; MS: n = 12 mice. $*p < 0.05$, $****p < 0.0001$, two-way RM ANOVA.

(K) The percentage of decrease in mechanical pain threshold. $**p < 0.01$, unpaired t test.

(L) Percentage of AFR and MS mice with or without a decrease in pain threshold.

Data are presented as means ± SEM. Further details of the statistical analysis are available in Table S1.

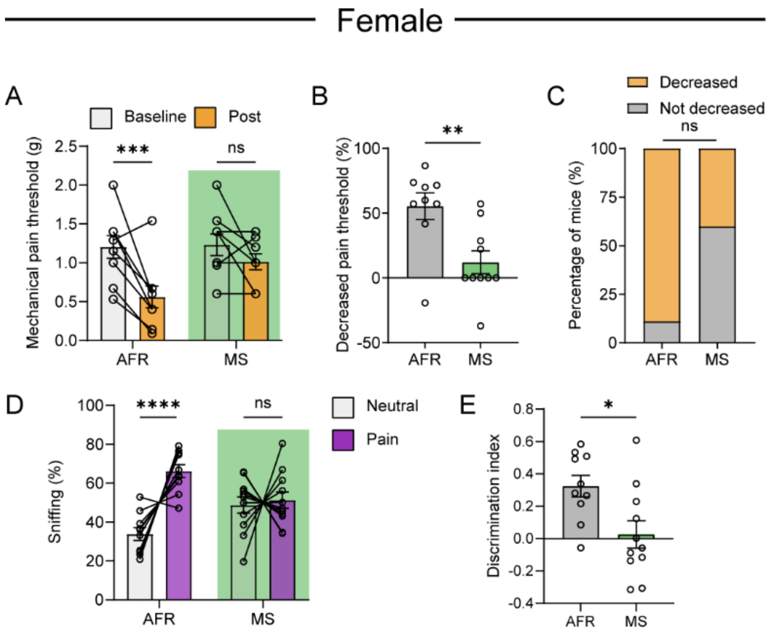

Fig. S3. Early life MS impaired the contagious pain in adult female mice.

(A) Mechanical pain threshold of AFR and MS female mice at both baseline and after 30 mins of social interaction with the painful demonstrator. Experiments were performed according to the protocol schematized in Figures 1A and 1D. AFR: n = 9 mice; MS: n = 10 mice. $***p < 0.001$, two-way RM ANOVA.

(B) Percentage decrease in mechanical pain threshold. $**p < 0.01$, unpaired t test.

(C) Percentage of AFR and MS female mice showing or not showing contagious pain.

(D) Percentage of sniffing time in the first two minutes of AFR and MS female observers. AFR: n = 10 mice; MS: n = 11 mice. $****p < 0.0001$, generalized linear

mixed model.

(E) Discrimination index in the first two minutes of AFR and MS female observers. *p < 0.05, unpaired t test.

Data are presented as means ± SEM. Further details of the statistical analysis are available in Table S1.

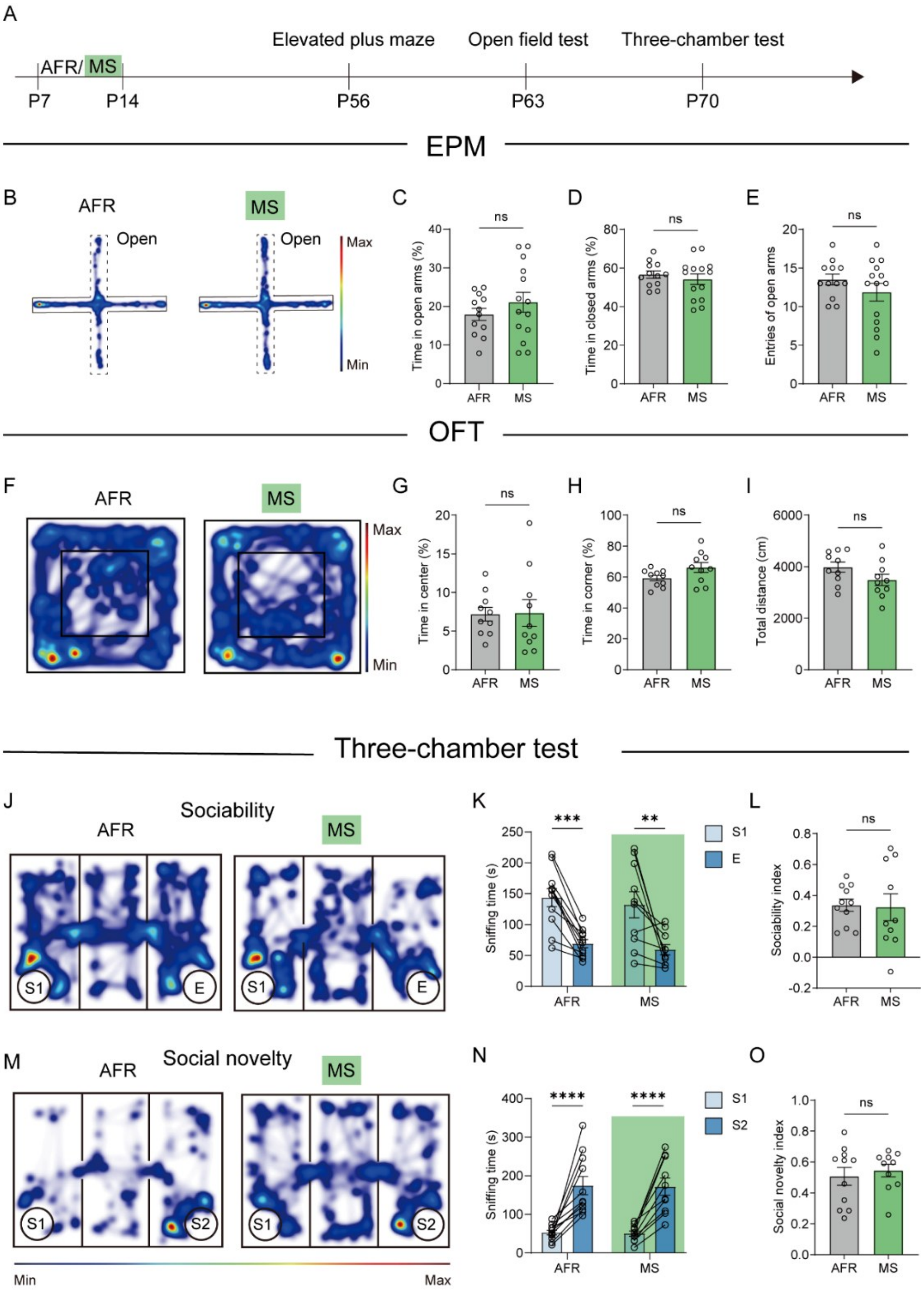

Fig. S4. Early life MS does not induce changes in anxiety, locomotion, sociability or social memory in adulthood.

(A) Schematic diagram showing the timeline of behavioral experiments in either AFR or MS mice.

(B) Representative heatmap depicting cumulative time spent at different locations of

the elevated plus maze (EPM).
(C and D) Percentage of time spent in open (C) and closed (D) arms.
(E) Number of entries into open arms. AFR: n = 12 mice; MS: n = 14 mice. ns, no significant difference, unpaired t test.
(F) Representative heatmap depicting cumulative time spent at different locations of the open field (OF).
(G and H) Percentage of time spent in the center (G) and the corner (H).
(I) Total distances travelled in the OF. AFR: n = 10 mice; MS: n = 10 mice. ns, no significant difference, unpaired t test.
(J) Representative heatmap depicting cumulative time spent at different locations in the sociability test.
(K) Sniffing time spent by either AFR or MS mice in the empty chamber or chamber with stringer 1.
(L) Sociability index derived from (K).
(M) Representative heatmap depicting cumulative time spent at different locations in the social novelty test.
(N) Sniffing time spent by either AFR or MS mice in the chamber with stranger 1 or stranger 2.
(O) Social novelty index derived from (N). AFR: n = 11 mice; MS: n = 10 mice. $^{**}p < 0.01$, $^{***}p < 0.001$, $^{****}p < 0.0001$, two-way RM ANOVA for (K and N). ns, no significant difference, Welch's t test or unpaired t test for (L and O).
Data are presented as means ± SEM. Further details of the statistical analysis are available in Table S1.

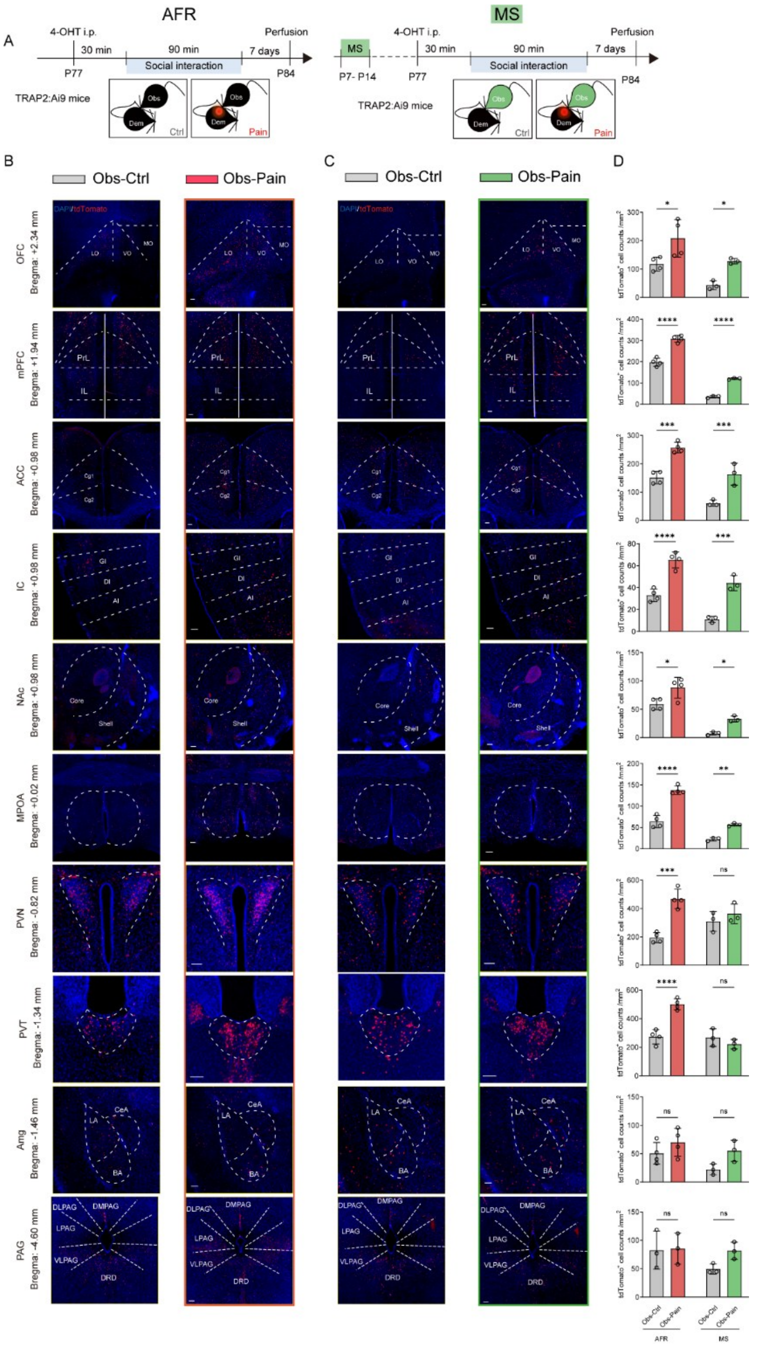

Fig. S5. Identification of brain regions potentially involved in MS-induced contagious pain deficits using the TRAP2:Ai9 labeling approach.

(A) Schematic of the timeline for labeling cells activated by observer-demonstrator social interaction. TRAP2:Ai9 mice were used as either AFR or MS observers.

(B) Representative images of tdTomato^{+} cells in several brain areas of AFR observers interacting with control (left) or painful (right) demonstrators. Scale bar: 100 µm. ACC: anterior cingulate cortex; Amg: amygdala; IC: insular cortex; mPFC: medial prefrontal cortex; MPOA: medial preoptic area; NAc: nucleus accumbens; OFC: orbitofrontal cortex; PAG: periaqueductal gray; PVN: paraventricular nucleus of the hypothalamus; PVT: paraventricular thalamus.

(C) Similar to (B) but for the representative images of MS observers.

(D) Quantification of tdTomato^{+} cell density for each brain area. AFR: For PAG, $n = 3$ mice for each group. For all other regions, $n = 4$ mice for each group. MS: $n = 3$ mice for each group. *$p < 0.05$, **$p < 0.01$, ***$p < 0.001$, ****$p < 0.0001$, two-way ANOVA. ns, no significant difference.

Data are presented as means ± SEM. Further details of the statistical analysis are available in Table S1.

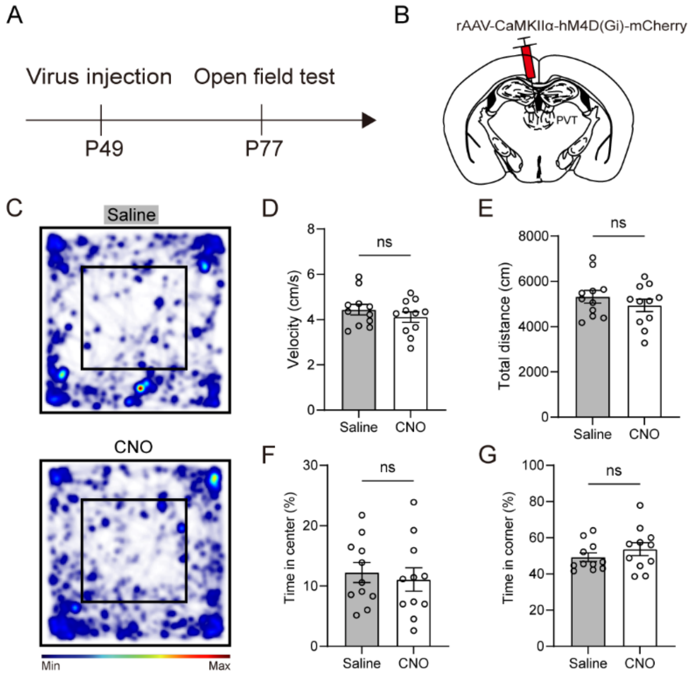

Fig. S6. Chemogenetic inhibition of PVT does not affect anxiety or locomotion.

(A) Schematic showing the timeline for examining the effect of PVT neuronal inhibition on anxiety or locomotion.

(B) Schematic of injection of AAV-CaMKIIα-hM4D(Gi)-mCherry into the PVT.

(C) Representative heatmap depicting cumulative time spent at different locations of the OF by saline- (up) and CNO-treated (bottom) animals.

(D and E) Locomotion parameters. Travelling speed (D) and total distance moved in the open arena (E).

(F and G) Anxiety parameters. Percentage of time spent in the center (F) and the corner (G). $n = 11$ mice for each group. ns, no significant difference, unpaired t test. Data are presented as means ± SEM. Further details of the statistical analysis are available in Table S1.

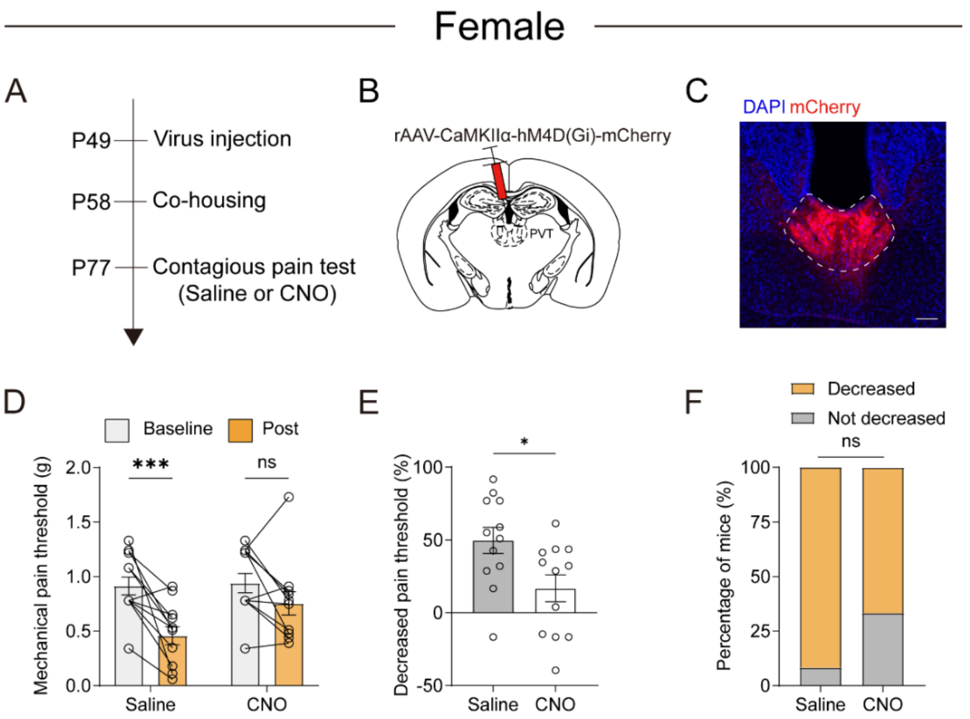

Fig. S7. PVT contributes to social transfer of pain in the female mice.

(A) Schematic of the timeline for examining the role of PVT glutamate neurons in contagious pain in female mice.

(B) Schematic of injection of AAV-CaMKIIα-hM4D(Gi)-mCherry into the PVT.

(C) One example image showing the histologic verification of viral expression in the PVT. Scale bar: 100 μm.

(D) Changes in mechanical pain threshold of AFR female observers injected with saline or CNO 30 min before social interaction with the painful demonstrator. n = 12 mice for each group. ***$p < 0.001$, two-way RM ANOVA.

(E) Percentage decrease in mechanical pain threshold. *$p < 0.05$, unpaired t test.

(F) Percentage of AFR female mice whose mechanical pain threshold decreased or not decreased after social interaction.

Data are presented as means ± SEM. Further details of the statistical analysis are available in Table S1.

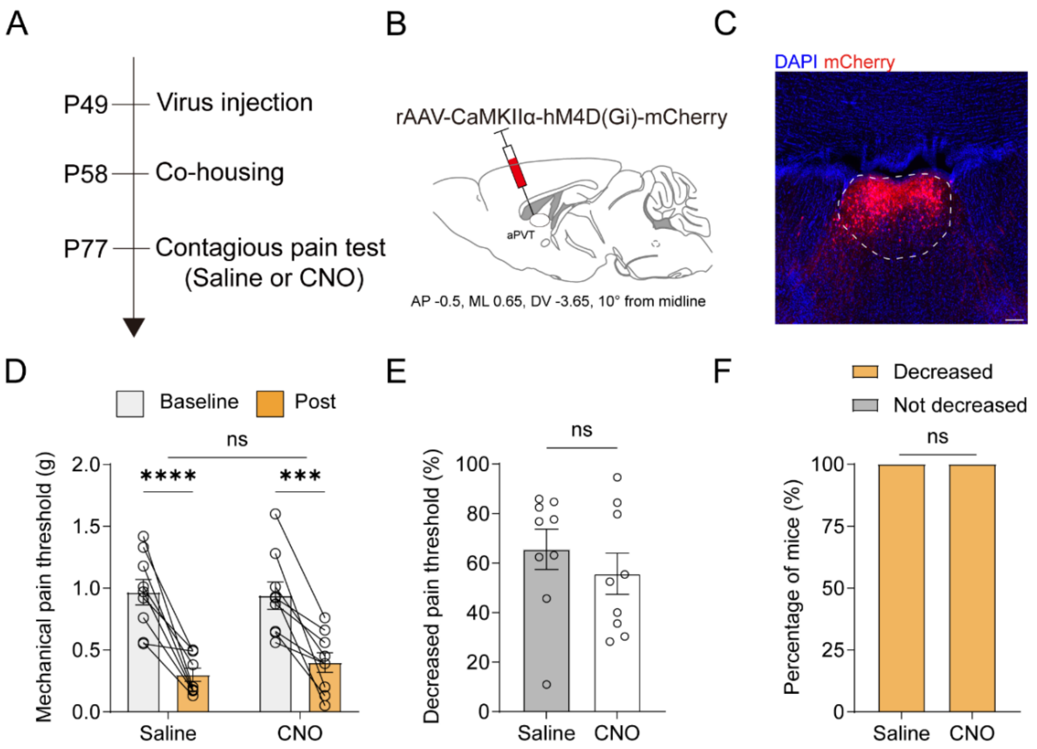

Fig. S8. Chemogenetic inactivation of anterior PVT neurons has no effect on contagious pain.

(A) Schematic of the timeline for investigating the role of anterior PVT glutamate neurons in pain contagion.

(B) Schematic of injection of AAV-CaMKIIα-hM4D(Gi)-mCherry into the anterior PVT. The injection coordinates for targeting this section of PVT are also shown.

(C) One example image showing the histologic verification of viral expression in the anterior PVT. Scale bar: 100 μm.

(D) Changes in mechanical pain threshold of AFR observers injected with CNO 30 min before social interaction with the painful demonstrator. Saline: $n = 9$ mice; CNO: $n = 9$ mice. ***$p < 0.001$, ****$p < 0.0001$, two-way RM ANOVA.

(E) Percentage decrease in mechanical pain threshold.

(F) Percentage of AFR mice showing or not showing contagious pain.

Data are presented as means ± SEM. Further details of the statistical analysis are available in Table S1.

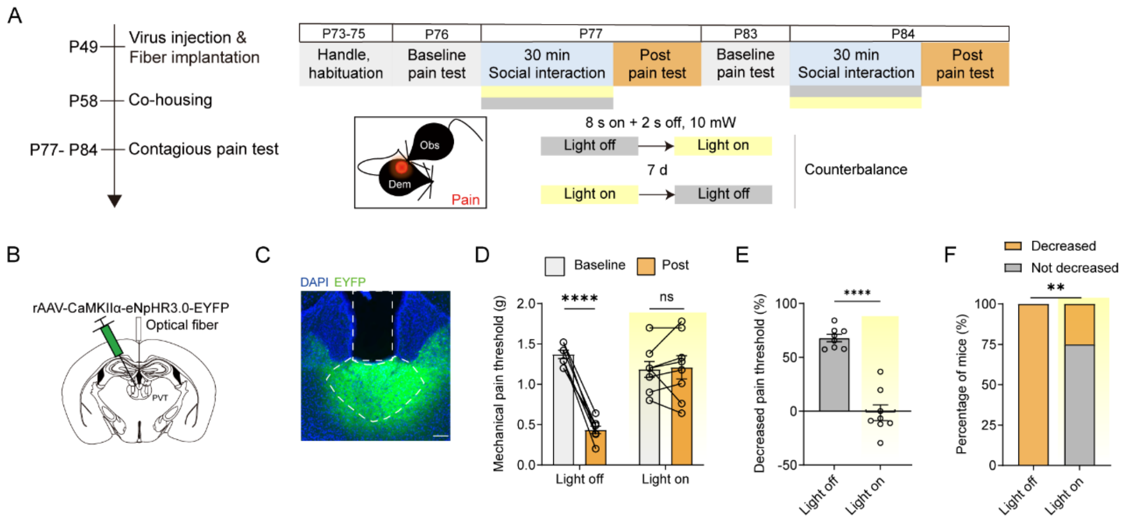

Fig. S9. Optogenetic inhibition of PVT glutamate neurons impairs the contagious pain.

(A) Schematic of the timeline for investigating the role of PVT glutamate neurons in the contagious pain with the optogenetic approach. The detailed light delivery protocol is also shown. For different animals, the time orders of ON (yellow bar) and OFF (grey bar) sessions were counter-balanced with a 7-day interval between tests.

(B) Schematic of injection of AAV-CaMKIIα-eNpHR3.0-EYFP into the PVT and implantation of optical fiber into the PVT.

(C) One representative image showing the histological verification of virus expression and optic fiber implantation. Scale bar: 100 µm.

(D) Changes in mechanical pain threshold of AFR observers for either light off or light on groups. Light off/Light on: $n = 8$ mice. ****$p < 0.0001$, two-way RM ANOVA.

(E) Percentage decrease in mechanical pain threshold. ****$p < 0.0001$, unpaired t test.

(F) Percentage of AFR mice showing or not showing contagious pain. **$p < 0.01$, Fisher's exact test.

Data are presented as means ± SEM. Further details of the statistical analysis are available in Table S1.

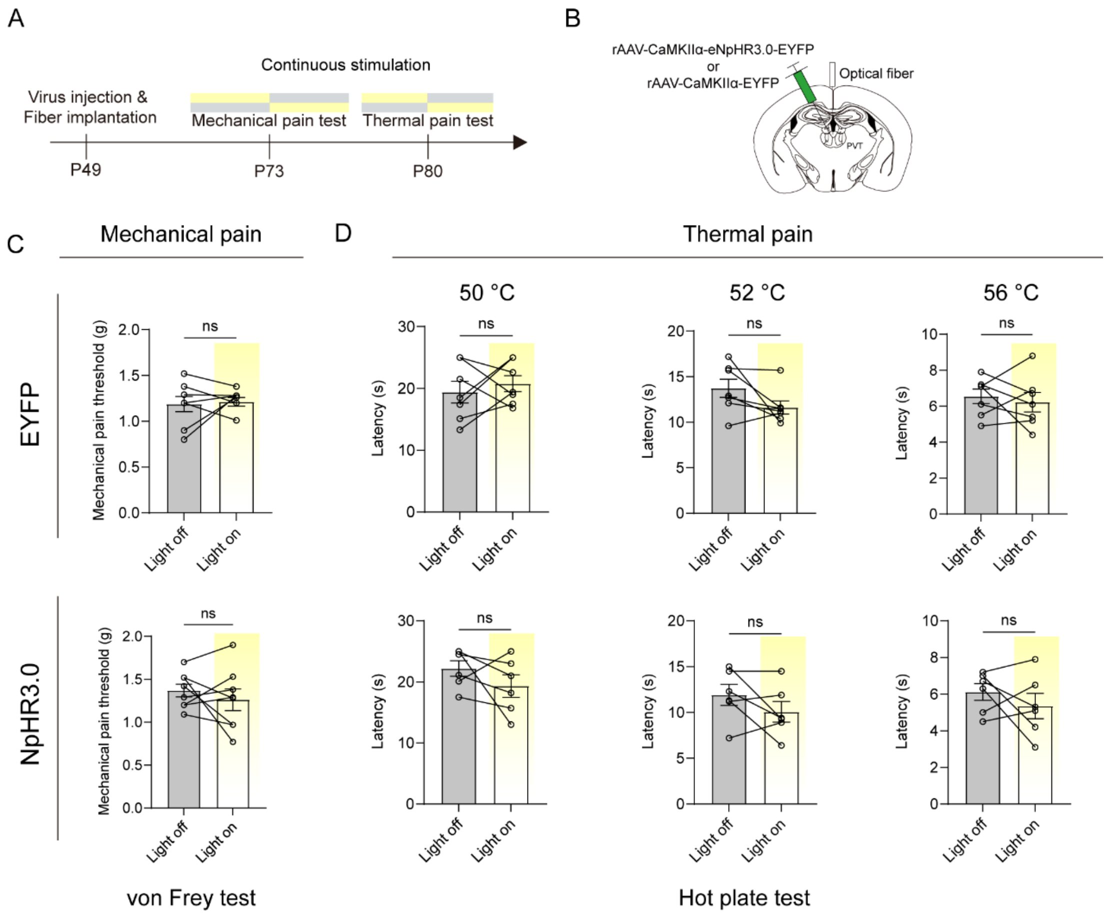

Fig. S10. Optogenetic inhibition of PVT glutamate neurons has no effect on baseline pain sensitivity.

(A) Schematic of the timeline for the behavioral experiment. The yellow light was continuously delivered for light on period during either mechanical or thermal pain test. For different animals, the orders of ON (orange bar) and OFF (grey bar) sessions were counter-balanced.

(B) Schematic of the viral strategy for optogenetic inhibition of PVT glutamate neurons.

(C) No significant differences in the mechanical pain threshold in the von Frey test. EYFP: *n* = 8 mice; NpHR: *n* = 8 mice.

(D) No significant differences in the latencies to flinch or lick the hindpaw in the hot plate test. EYFP: *n* = 6 mice; NpHR: *n* = 7 mice.

ns, no significant difference, paired t test. Data are presented as means ± SEM. Further details of the statistical analysis are available in Table S1.

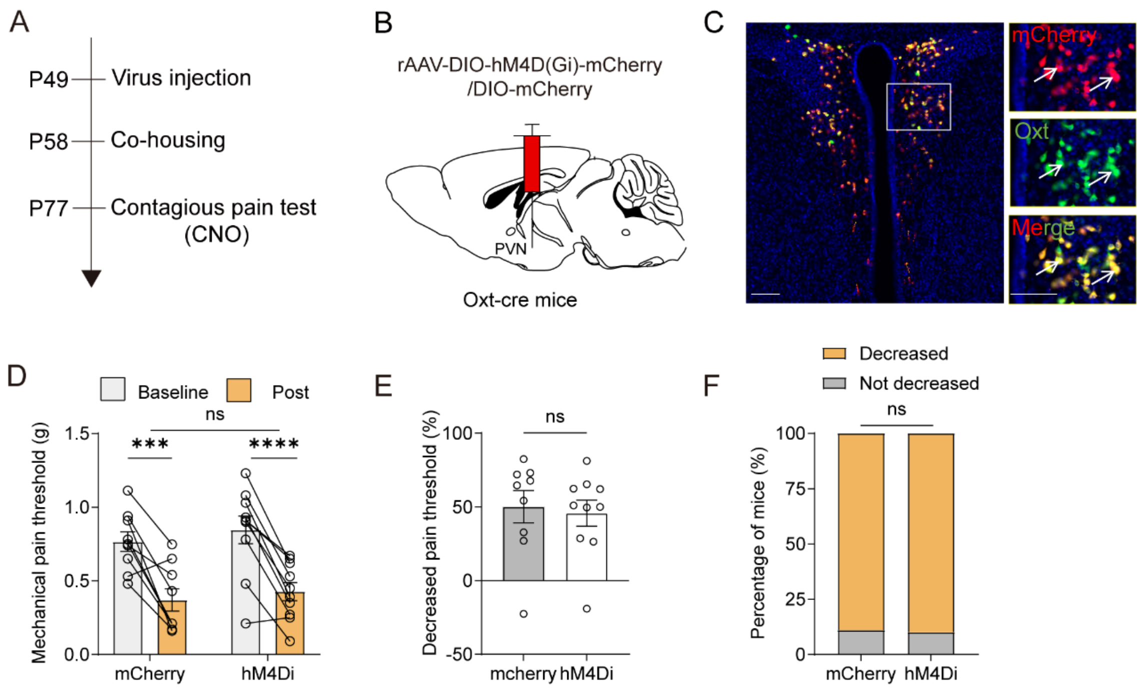

Fig. S11. Chemogenetic inactivation of PVN oxytocin neurons has no effect on the contagious pain.

(A) Schematic of the timeline for investigating the role of PVN oxytocin neurons in pain contagion. PVN: paraventricular nucleus of the hypothalamus.

(B) Schematic of injection of AAV-DIO-hM4D(Gi)-mCherry into the PVN of the Oxt-cre mice.

(C) Histologic verification of viral expression in oxytocin neurons. Right: Enlarged view of the white boxed area in the left picture, showing selective co-labeling of mCherry with oxytocin in the PVN. Scale bar: 100 µm.

(D) Changes in mechanical pain threshold of AFR observers injected with CNO 30 min before social interaction with the painful demonstrator. AAV-DIO-mCherry: $n = 9$ mice; AAV-DIO-hM4D(Gi)-mCherry: $n = 10$ mice. $***p < 0.001$, $****p < 0.0001$, two-way RM ANOVA.

(E) Percentage decrease in mechanical pain threshold.

(F) Percentage of AFR mice showing or not showing the contagious pain.

Data are presented as means ± SEM. Further details of the statistical analysis are available in Table S1.

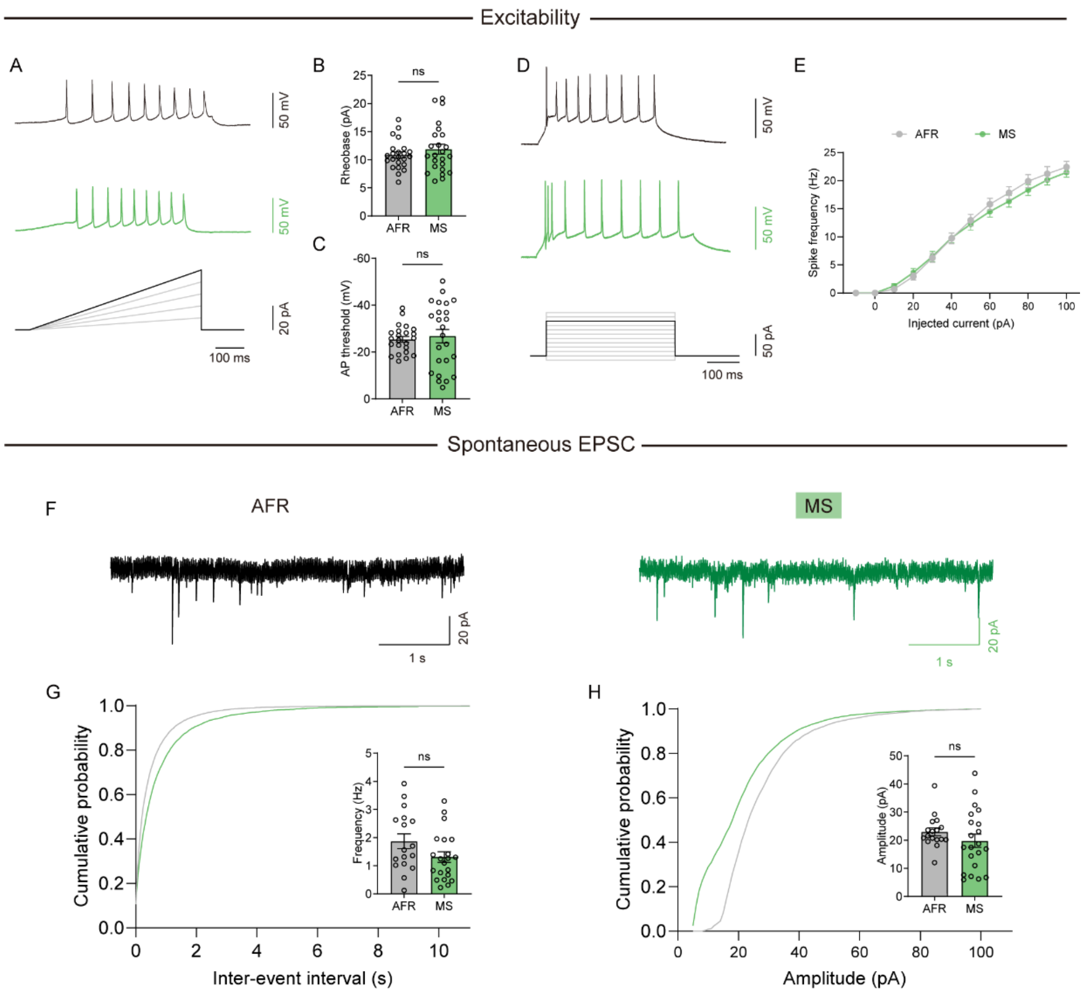

Fig. S12. Early life MS could not alter the excitability of PVT neurons at P14.

(A) Representative traces of action potentials (APs) of PVT neurons recorded under a ramping current injection. Dark gray: traces from AFR group: Green: traces from MS group.

(B and C) AP rheobase (B) and AP threshold (C) obtained from the PVT neurons of AFR and MS mice. *n* = 23 cells from six mice for AFR and *n* = 24 cells from six mice for MS.

(D) Representative traces of APs in response to 80 pA current injections in AFR- and MS-treated PVT neurons.

(E) AP frequency in response to a range of current injections for PVT neurons from AFR- and MS-treated mice. *n* = 22 cells from six mice for AFR and *n* = 25 cells from six mice for MS.

(F) Representative traces of the spontaneous excitatory postsynaptic current (sEPSC) recorded from PVT neurons of AFR and MS mice.

(G) Cumulative probability plots of the inter-event interval and average sEPSC frequency of PVT neurons from AFR- and MS-treated mice.

(H) Cumulative probability plots and average sEPSC amplitude of PVT neurons from AFR- and MS-treated mice. *n* = 17 cells from three mice for AFR and *n* = 21 cells from three mice for MS.

ns, no significant difference, two-way RM ANOVA, Welch's t test or unpaired t test. Data are presented as means ± SEM. Further details of the statistical analysis are available in Table S1.

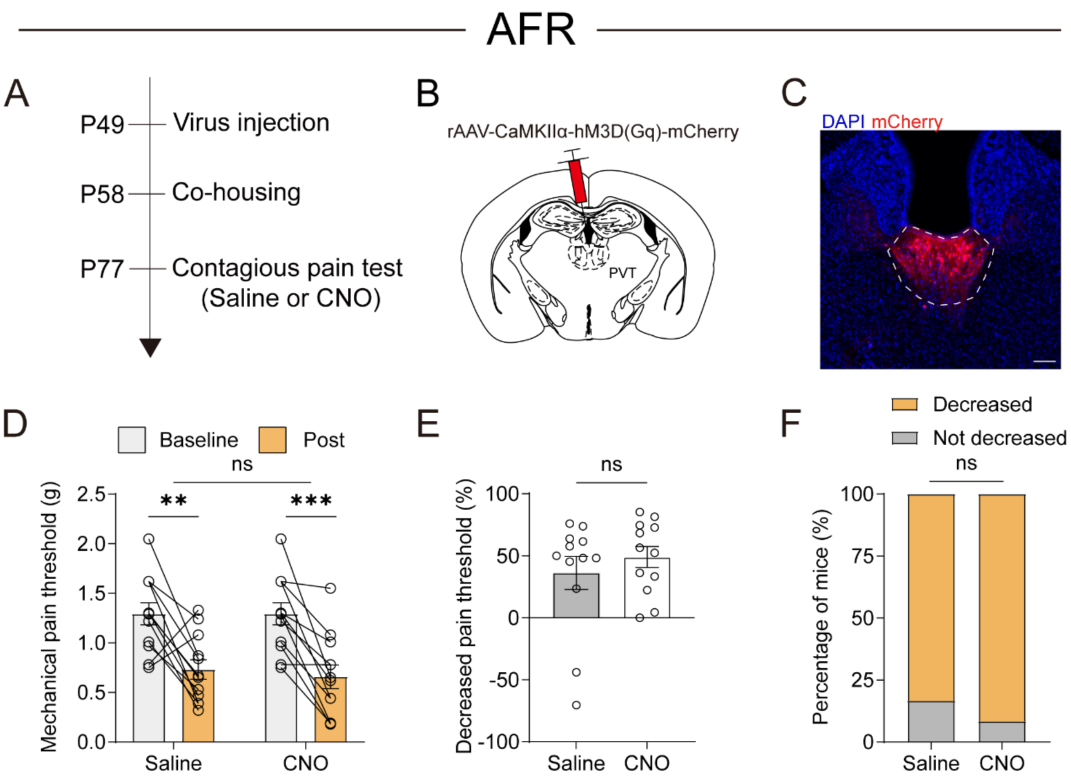

Fig. S13. Chemogenetic activation of PVT glutamate neurons does not further enhance contagious pain in the AFR mice.

(A) Schematic of the timeline for examining the effect of chemogenetic activation of PVT glutamate neurons on contagious pain in the AFR mice.

(B) Schematic of injection of AAV-CaMKIIα-hM3D(Gq)-mCherry into the PVT for chemogenetic activation of PVT glutamate neurons.

(C) Histologic verification of viral expression in the PVT. Scale bar: 100 μm.

(D) Changes in mechanical pain threshold of AFR observers injected with saline or CNO 30 min before social interaction with the painful demonstrator. Saline: n = 12 mice; CNO: n = 12 mice. $^{**}p < 0.01$, $^{***}p < 0.001$, two-way RM ANOVA.

(E) Percentage of decrease in mechanical pain threshold.

(F) Percentage of AFR mice whose mechanical pain threshold decreased or not decreased after social interaction.

Data are presented as means ± SEM. Further details of the statistical analysis are available in Table S1.

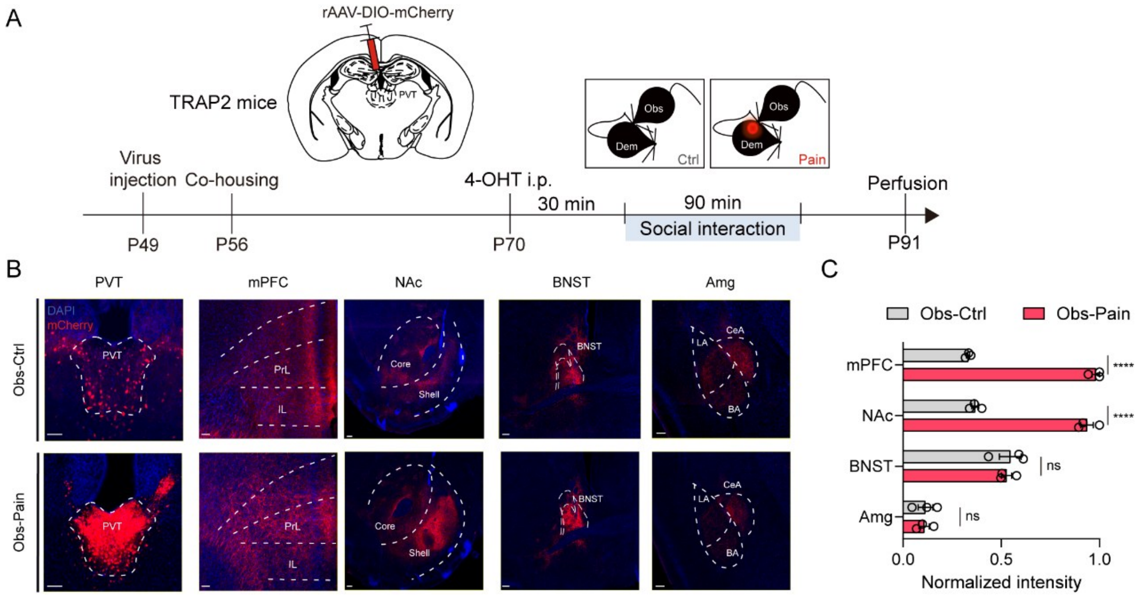

Fig. S14. Whole-brain tracing of projections of PVT neurons activated by observer-demonstrator interactions.

(A) Schematic of the experiment timeline and viral strategy for labeling PVT neurons activated by observer-demonstrator social interaction using the TRAP2 mice.

(B) Represented images showing the PVT neuron projection terminals in selected brain regions for observers interacting with control (top) or painful (bottom) demonstrators. AAV-DIO-mCherry was injected into the PVT (left). mCherry^{+} projection fibers could be evidently seen in Amg (amygdala), BNST (bed nuclei of stria terminalis), mPFC (medial prefrontal cortex), and NAc (nucleus accumbens). Scale bar: 100 μm.

(C) Quantifications of the TRAP2-labeled PVT neuron projection intensity in brain areas mentioned above. n = 3 mice for each group. ****$p < 0.0001$, unpaired t test.

Data are presented as means ± SEM. Further details of the statistical analysis are available in Table S1.

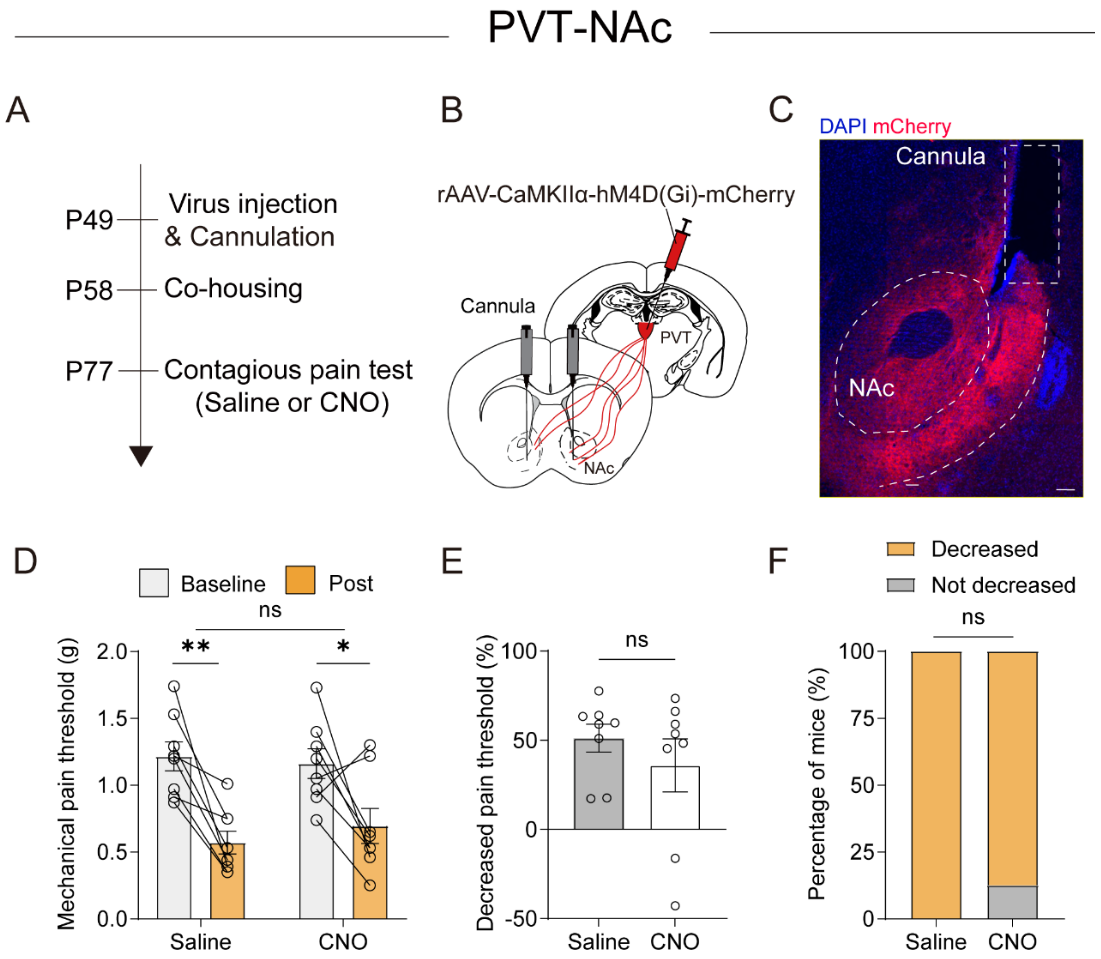

Fig. S15. Inhibition of the PVT→NAc projection does not affect the contagious pain.

(A) Schematic of the timeline for examining the role of the PVT→NAc circuit in contagious pain.

(B) Schematic of injection of AAV-CaMKIIα-hM4D(Gi)-mCherry into the PVT and implantation of cannula into the NAc.

(C) Histologic verification of $mCherry^{+}$ terminal expression and cannula implantation in the NAc. Scale bar: 100 μm.

(D) Changes in mechanical pain threshold of AFR observers injected with saline or CNO 30 min before social interaction with the painful demonstrator. n = 8 mice for each group. *$p < 0.05$, **$p < 0.01$, two-way RM ANOVA.

(E) Percentage of decrease in mechanical pain threshold.

(F) Percentage of AFR mice whose mechanical pain threshold decreased or not decreased after social interaction.

Data are presented as means ± SEM. Further details of the statistical analysis are available in Table S1.

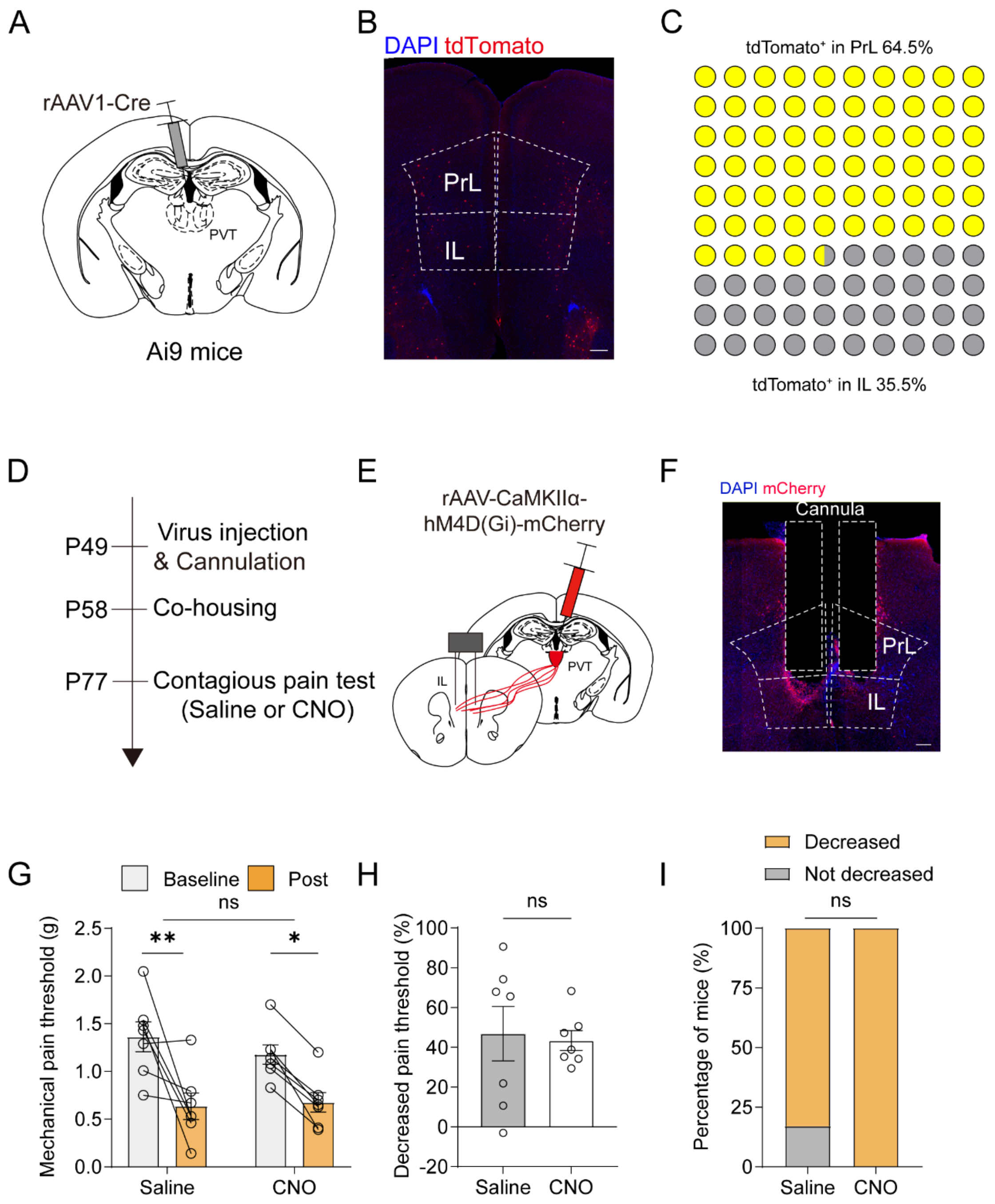

Fig. S16. Anatomical and functional dissection of PVT-innervated mPFC subregions contributing to the contagious pain.

(A) Schematic of the viral strategy for labeling the PVT-innervated brain regions through injecting the AAV1-Cre into the PVT of the Ai9 mice.

(B) One example image showing the distribution of tdTomato$^+$ cells in the PrL and IL subregions of the mPFC. Scale bar: 100 μm.

(C) Quantitative analysis of the percentage of tdTomato$^+$ cells in the PrL (yellow circles) vs IL (gray circles).

(D) Schematic of the timeline for examining the role of the PVT→IL circuit in contagious pain.

(E) Schematic of injection of AAV-CaMKIIα-hM4D(Gi)-mCherry into the PVT and

implantation of cannula into the IL.
(F) Histologic verification of mCherry^{+} terminal expression and cannula implantation in the IL. Scale bar: 100 µm.
(G) Changes in mechanical pain threshold of AFR observers injected with saline or CNO 30 min before social interaction with the painful demonstrator. n = 7 mice for each group. *p < 0.05, **p < 0.01, two-way RM ANOVA.
(H) Percentage of decrease in mechanical pain threshold.
(I) Percentage of AFR mice whose mechanical pain threshold decreased or not decreased after social interaction.
Data are presented as means ± SEM. Further details of the statistical analysis are available in Table S1.

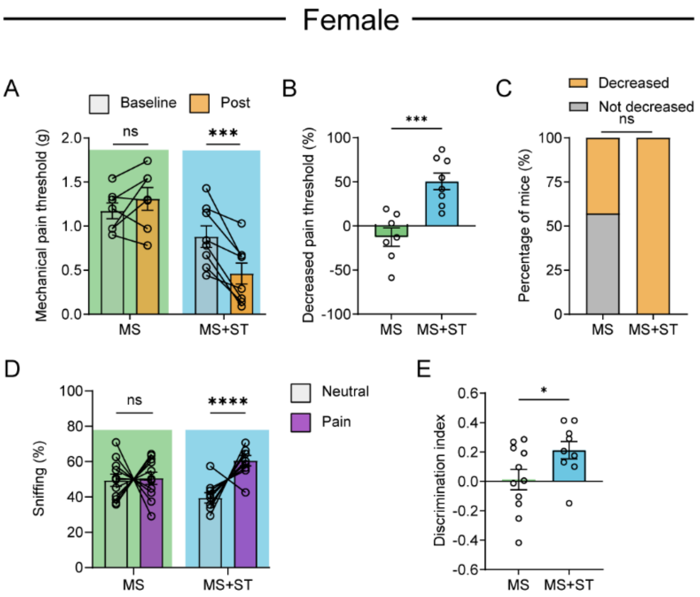

Fig. S17. Social touch-like tactile stimulation rescues MS-induced contagious pain deficits in the female mice.
(A) Changes in mechanical pain threshold of MS-inflicted female observers receiving or not receiving early-life social touch (ST) treatment. The ST protocol and experimental timeline were similar as those shown in Figure 7A. MS: n = 7 mice; MS + ST: n = 8 mice. ***p < 0.001, two-way RM ANOVA.
(B) Percentage of decrease in mechanical pain threshold. MS: n = 7 mice; MS + ST: n = 8 mice. ***p < 0.001, unpaired t test.
(C) Percentage of female observer mice showing or not showing nociceptive hypersensitivity after interacting with the painful demonstrator.
(D) Percentage of sniffing time in the first two minutes of MS and MS + ST female observers. MS: n = 11 mice; MS + ST: n = 9 mice. ****p < 0.0001, generalized linear mixed model.
(E) Discrimination index derived from the data shown in (D). *p < 0.05, unpaired t test.
Data are presented as means ± SEM. Further details of the statistical analysis are available in Table S1.

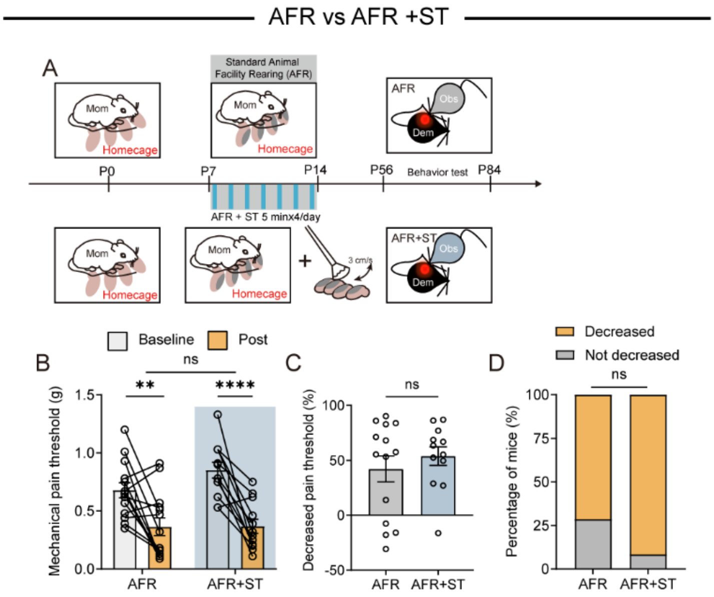

Fig. S18. Social touch-like tactile stimulation could not further enhance contagious pain in the AFR mice.

(A) Schematic of the timeline for examining the effect of early-life social touch (ST)-like tactile stimulation on contagious pain in AFR mice.

(B) Changes in mechanical pain threshold of AFR observers receiving or not receiving early-life ST treatment. AFR: n = 14 mice; AFR+ ST: n = 12 mice. $^{**}p < 0.01$, $^{****}p < 0.0001$, two-way RM ANOVA.

(C) Percentage of decrease in mechanical pain threshold.

(D) Percentage of AFR mice whose mechanical pain threshold decreased or not decreased after social interaction.

Data are presented as means ± SEM. Further details of the statistical analysis are available in Table S1.

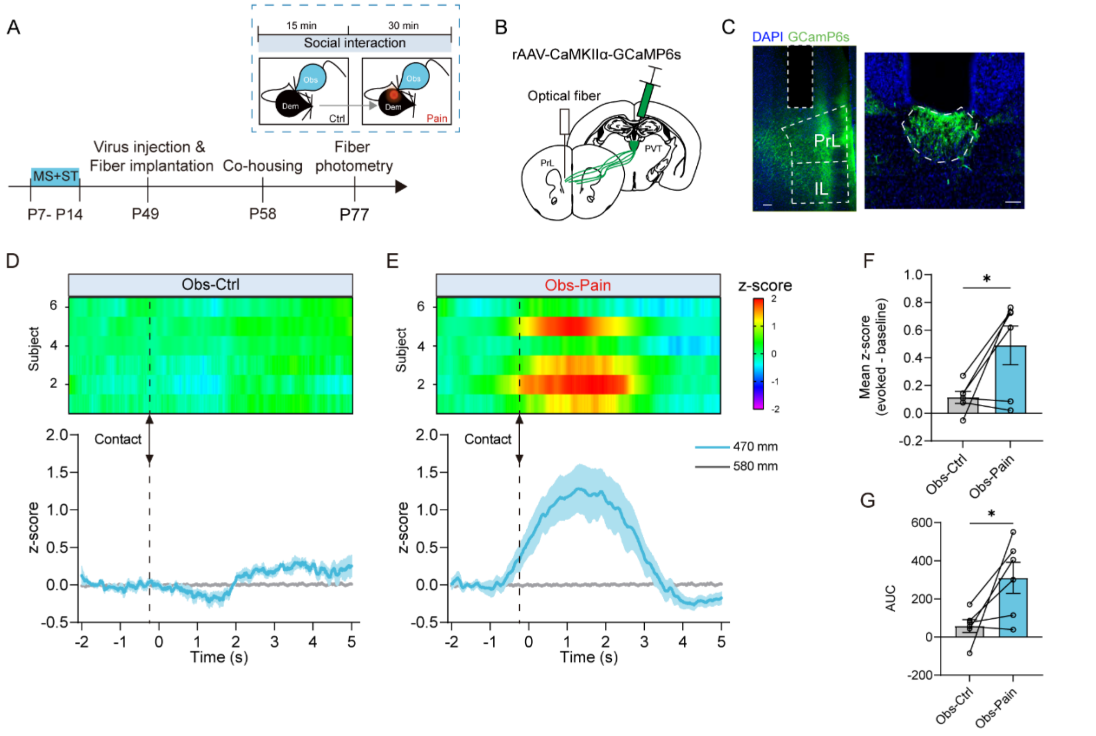

Fig. S19. Social touch-like tactile stimulation rescues the activation of the PVT→ PrL circuit during observer-demonstrator interaction in the MS mice.

(A) Schematic of the experimental design for fiber photometry recording of Ca^{2+} signals in the PVT→PrL circuit in the MS + ST mice.

(B) Schematic of injection of AAV-CaMKIIα-GCaMP6s into the PVT and implantation of optical fiber into the PrL.

(C) One example image showing the histological verification of viral expression and optic fiber implantation in the PrL. Scale bar: 100 µm.

(D and E) (Top) Heatmap of Ca^{2+} signals aligned with the onset of body contact between MS + ST observers and control (D) or painful demonstrators (E). (Bottom) Peri-stimulus time histogram of average Ca^{2+} signals.

(F and G) Quantification of the change in GCaMP6s signal as either mean z-score (F) or averaged area under curve (AUC) (G). $n = 6$ mice. *$p < 0.05$, paired t test.

Data are presented as means ± SEM. Further details of the statistical analysis are available in Table S1.

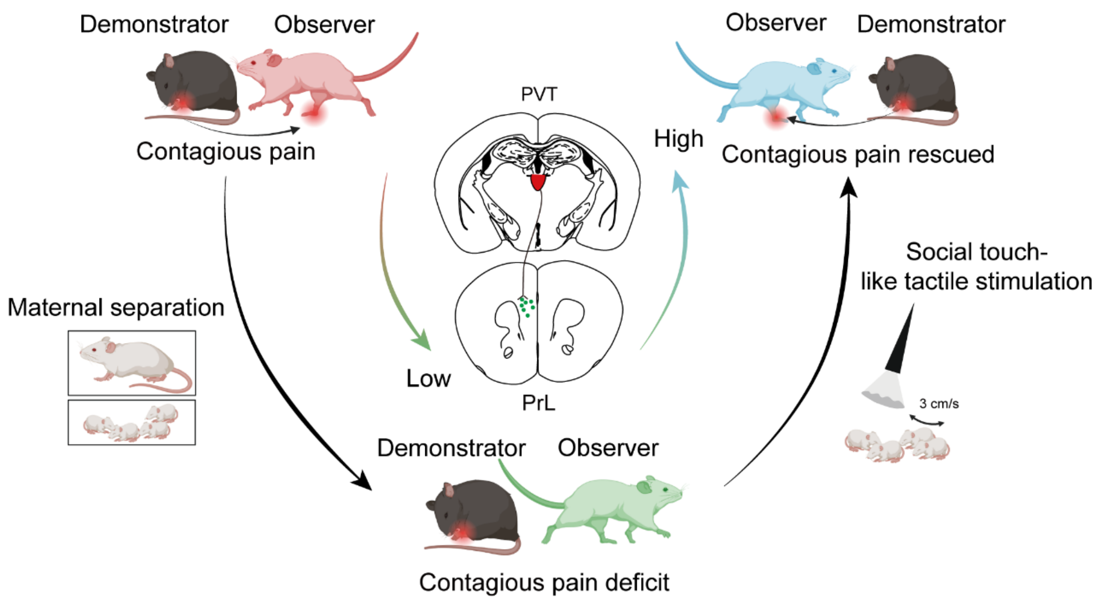

Fig. S20. A schematic model showing the circuit basis of MS-evoked contagious pain deficits and their rescue by affiliative social touch stimulation.
Early life stress, such as maternal separation (MS), results in a marked hypoactivation of the PVT→PrL circuit activity, conferring deficits in contagions pain when observes socially interact with the painful demonstrators. Artificial chemogenetic activation of PVT glutamate neurons or the PVT→PrL projection significantly restores the ability of MS-inflicted observer mice to exhibit the pain contagion. Importantly, repetitive social touch-like tactile stimulation during the early life period prevents the MS-induced contagious pain loss through elevating the PVT→PrL circuit activity.

Supplementary movies

Movie S1: Fiber photometry recording of PVT glutamate neurons in one AFR observer during social interaction with the painful demonstrator. The onset of each body contact event is preceded by an early increase in the Ca^{2+} signal of PVT glutamate neurons (indicated with a yellow pointer), which persists during the entire contact period. AFR: animal facility rearing.

Movie S2: Fiber photometry recording of PVT glutamate neurons in one MS observer during social interaction with the painful demonstrator. No any significant rise in Ca^{2+} signal of PVT glutamate neurons was detected during each bout of observer-demonstrator social interaction. MS: maternal separation.

Movie S3: Fiber photometry recording of PVT → PrL projection terminals in one AFR observer during social interaction with the painful demonstrator. The Ca^{2+} dynamics from the PVT → PrL circuit increased immediately before each bout of body contact and remained elevated throughout the entire contact period.

Movie S4: Fiber photometry recording of PVT → PrL projection terminals in one MS

+ ST observer during social interaction with the painful demonstrator. Social touch-like tactile stimulation results in clear activation of the PVT→ PrL circuit in the MS-treated observer when socially interacting with one painful demonstrator. ST: social touch.

Table S1. The detailed statistical information for all figures of this study.
