# Supplementary material for: Dysfunction of the paraventricular thalamus–prelimbic cortex circuit underlies maternal separation–induced deficits in contagious pain
Source: Sci Adv. 2025 Oct 8;11(41):eady1944. doi: 10.1126/sciadv.ady1944 (PMC12506963; doi:10.1126/sciadv.ady1944)
Supplement: Supplementary file 3 — Table S1 [file sciadv.ady1944_table_s1.zip › ady1944_table_s1.docx]

| **Supplemental Table 1. The detailed statistical information for all figures of this study** |
| --- |

| **Figure** | **Response variable** | **Analysis** | **Sample size** | **Results** | | | |
| --- | --- | --- | --- | --- | --- | --- | --- |
| 1B | Mechanical pain threshold | Two-way RM ANOVA followed by Dunnett's multiple comparisons test within each group, comparing each time point with baseline Factor1: Time (Baseline vs Post, 2 h, 4 h, 8 h, 24 h) Factor2: Dem (Ctrl (Saline) vs Pain (BV)) | Obs-Ctrl (Saline) = 12 Obs-Pain (BV) = 12 | Time x Dem, F(5, 110) = 9.157 P<0.0001 Time: F (3.112, 68.46) =11.24 P<0.0001 Dem: F (1, 22) = 10.70 P=0.0035 Subject: F (22, 110) = 2.785 P=0.0002  Multiple comparison P:  Obs-Ctrl (Saline) Obs-Pain (BV) Baseline vs. Post 0.9999 0.0009 Baseline vs. 2 h 0.0586 0.0002 Baseline vs. 4 h 0.3816 0.0038 Baseline vs. 8 h 0.8824 0.9784 Baseline vs. 24 h 0.4319 0.5992 | | | |
| 1C | Mechanical pain threshold | Two-way RM ANOVA followed by Sidak's multiple comparisons test within each group, comparing each time point with baseline Factor1: SI duration (15 min vs 30 min) Factor2: Time (Baseline vs Post) | SI-15 min = 9 SI-30 min = 12 | SI duration x Time: F (1, 19) = 0.7095, P=0.4101 SI duration: F (1, 19) = 2.011 P=0.1723 Time: F (1, 19) = 11.68 P=0.0029 Subject: F (19, 19) = 2.196 P=0.0474  Multiple comparison P: Baseline vs Post: SI-15 min 0.1986 SI-30 min 0.0083 | | | |
| 1E | Mechanical pain threshold | Two-way RM ANOVA followed by Sidak's multiple comparisons test within each group, comparing post with baseline, and Sidak's multiple comparisons test within baseline, comparing MS with AFR group Factor1: Group (AFR vs MS) Factor2: Time (Baseline vs Post) | AFR = 15 MS =15 | Group x Time: F (1, 28) = 10.54 P=0.0030 Group: F (1, 28) = 1.212 P=0.2803 Time: F (1, 28) = 20.02 P=0.0001 Subject: F (28, 28) = 5.713 P<0.0001  Multiple comparison P: Baseline vs Post: AFR <0.0001 MS 0.6306  AFR vs MS  Baseline 0.9652 | | | |
| 1F | Percentage of decreased mechanical pain threshold | Unpaired t test | AFR = 15 MS =15 | t=3.021, df=28, two tailed P=0.0053 | | | |
| 1G | Percentage of mice with decreased pain threshold | Fisher's exact test | AFR = 15 MS =15 | P=0.1086 | | | |
| 1K | Sniffing time | Two-way RM ANOVA followed by Sidak's multiple comparisons test within each time session, comparing pain with neutral Factor1: Time (0-2 min vs 2-4 min vs 4-6 min) Factor2: Emotion (Neutral vs Pain) | Neutral = Pain = 17 | Time x Emotion: F (2, 64) = 2.190 P=0.1202 Time: F (1.802, 57.68) = 0.4407 P=0.6252 Emotion: F (1, 32) = 30.39 P<0.0001 Subject: F (32, 64) = 1.827 P=0.0204  Multiple comparison P: Neutral vs Pain: 0-2 min <0.0001 2-4 min 0.0007 4-6 min 0.0903 | | | |
| 1L | Sniffing time | Two-way RM ANOVA followed by Sidak's multiple comparisons test within each time session, comparing pain with neutral Factor1: Time (0-2 min vs 2-4 min vs 4-6 min) Factor2: Emotion (Neutral vs Pain) | Neutral = Pain = 13 | Time x Emotion: F (2, 48) = 0.9042 P=0.4117 Time: F (1.967, 47.21) = 0.8851 P=0.4179 Emotion: F (1, 24) = 0.0474 P=0.8295 Subject: F (24, 48) = 1.510 P=0.1113  Multiple comparison P: Neutral vs Pain: 0-2 min 0.8955 2-4 min 0.8180 4-6 min 0.8615 | | | |
| 1M | Percentage of sniffing time in 0-2 min | Generalized linear mixed model  Fixed effects: Group ×Emotion interaction (full factorial design)  Random effects: Subject-specific random intercepts (1\|subject) Group (AFR vs MS), Emotion (Neutral vs Pain) | AFR = 17 MS = 13 | Significant group × emotion interaction Group x Emotion: F (1, 56) = 18.92 P<0.0001, β = -26.50, 95% CI [-38.71, -14.30]  Simple effect: Neutral vs Pain AFR β = 22.56 t(32) = 5.75, <0.0001 MS β = -3.94 t(24) = -0.84, 0.6516 | | | |
| 1N | Discrimination index | Unpaired t test | AFR = 17 MS = 13 | t=2.971, df=28, two tailed P=0.0060 | | | |
| 2F | Mean z-score (evoked - baseline) | Paired t test | Obs-Ctrl = Obs-Pain = 8 | t=5.808, df=7, two tailed P=0.0007 | | | |
| 2G | AUC | Paired t test | Obs-Ctrl = Obs-Pain = 8 | t=5.564, df=7, two tailed P=0.0008 | | | |
| 2L | Membrane potential | Paired t test | Control = CNO = 8 cells | t=10.16, df=7, two tailed P<0.0001 | | | |
| 2M | Mechanical pain threshold | Two-way RM ANOVA followed by Sidak's multiple comparisons test within each treatment, comparing post with baseline Factor1: Treatment (Saline vs CNO) Factor2: Time (Baseline vs Post) | Saline = 8 CNO = 8 | Treatment x Time: F (1, 14) = 9.412 P=0.0083 Treatment: F (1, 14) = 1.807 P=0.2002 Time: F (1, 14) = 20.04 P=0.0005 Subject: F (14, 14) = 2.328 P=0.0629  Multiple comparison P: Baseline vs Post: Saline 0.0002 CNO 0.5594 | | | |
| 2N | Percentage of decreased mechanical pain threshold | Unpaired t test | Saline = 8 CNO = 8 | t=3.989, df=14, two tailed P=0.0013 | | | |
| 2O | Percentage of mice with decreased pain threshold | Fisher’s exact test | Saline = 8 CNO = 8 | P=0.2000 | | | |
| 2P | Sniffing time | Two-way RM ANOVA followed by Sidak's multiple comparisons test within each time session, comparing pain with neutral Factor1: Time (0-2 min vs 2-4 min vs 4-6 min) Factor2: Emotion (Neutral vs Pain) | Neutral = Pain = 11 | Time x Emotion: F (2, 40) = 9.230 P=0.0005 Time: F (1.786, 35.72) = 0.2845 P=0.7294 Emotion: F (1, 20) = 1.362 P=0.2569 Subject: F (20, 40) = 1.180 P=0.3188  Multiple comparison P: Neutral vs Pain: 0-2 min 0.0007 2-4 min 0.9693 4-6 min 0.4053 | | | |
| 2Q | Sniffing time | Two-way RM ANOVA followed by Sidak's multiple comparisons test within each time session, comparing pain with neutral Factor1: Time (0-2 min vs 2-4 min vs 4-6 min) Factor2: Emotion (Neutral vs Pain) | Neutral = Pain = 12 | Time x Emotion: F (2, 44) = 1.735, P=0.1882  Time: F (1.860, 40.92) = 0.03163, P=0.9619  Emotion: F (1, 22) = 1.137, P=0.2979  Subject: F (22, 44) = 0.5930, P=0.9061  Multiple comparison P: Neutral vs Pain: 0-2 min 0.5373 2-4 min 0.2551 4-6 min 0.7359 | | | |
| 2R | Percentage of sniffing time in 0-2 min | Generalized linear mixed model  Fixed effects: Treatment× Emotion interaction (full factorial design)  Random effects: Subject-specific random intercepts (1\|subject)  Treatment (Saline vs CNO), Emotion (Neutral vs Pain) | Saline = 11 CNO = 12 | Significant group × emotion interaction:  Treatment× Emotion: F(1,42) = 16.69, P < 0.0001, β = -34.37, 95% CI [-51.35, -17.39]  Simple effect (Neutral vs Pain):  Saline β = 26.54, t(20) = 4.70, P < 0.0001  CNO β = -7.83, t(22) = -1.27, P = 0.2177 | | | |
| 2S | Discrimination index | Unpaired t test | Saline = 11 CNO = 12 | t=2.760, df=21, two tailed P=0.0117 | | | |
| 3F | Mean z-score (evoked - baseline) | Paired t test | Obs-Ctrl = Obs-Pain = 8 | t=0.1279, df=7, two tailed P=0.9018 | | | |
| 3G | AUC of ΔF/F | Paired t test | Obs-Ctrl = Obs-Pain = 8 | t=0.8434, df=7, two tailed P=0.4269 | | | |
| 3I | Rheobase | Welch's t test | AFR = 22 cells  MS = 25 cells | t=5.904, df=36.24, two tailed P<0.0001 | | | |
| 3J | AP threshold | Unpaired t test | AFR = 22 cells  MS = 25 cells | t=1.377, df=45, two tailed P=0.1753 | | | |
| 3L | Spike frequency | Two-way RM ANOVA  Factor1: Injected currents Factor2: Group (AFR vs MS) | AFR = 22 cells  MS = 25 cells | Inject currents x Group: F (11, 495) = 9.083 P<0.0001 Inject currents: F (11, 495) = 224.3 P<0.0001 Group: F (1, 45) = 24.57 P<0.0001 Subject: F (45, 495) = 11.34 P<0.0001 | | | |
| 3N-right | Frequency | Welch's t test | AFR = 23 cells  MS = 25 cells | t=2.997, df=33.68, two tailed P=0.0051 | | | |
| 3O-right | Amplitude | Unpaired t test | AFR = 23 cells  MS = 25 cells | t=0.8855, df=46, two tailed P=0.3805 | | | |
| 4E | Firing frequency | Paired t test | Control = CNO = 8 cells | t=3.893, df=7, two tailed P=0.0060 | | | |
| 4F | Mechanical pain threshold | Two-way RM ANOVA followed by Sidak's multiple comparisons test within each treatment, comparing post with baseline Factor1: Treatment (Saline vs CNO) Factor2: Time (Baseline vs Post) | Saline = 9 CNO = 9 | Treatment x Time: F (1, 16) = 7.356 P=0.0154 Treatment: F (1, 16) = 3.720 P=0.0717 Time: F (1, 24) = 15.18 P=0.0013 Subject: F (16, 16) = 1.626 P=0.1704  Multiple comparison P: Baseline vs Post: Saline 0.6577 CNO 0.0005 | | | |
| 4G | Percentage of decreased mechanical pain threshold | Unpaired t test | Saline = 9 CNO = 9 | t=2.993, df=16, two tailed P=0.0086 | | | |
| 4H | Percentage of mice with decreased pain threshold | Fisher’s exact test | Saline = 9 CNO = 9 | P=0.2941 | | | |
| 4I | Sniffing time | Two-way RM ANOVA followed by Sidak's multiple comparisons test within each time session, comparing pain with neutral Factor1: Time (0-2 min vs 2-4 min vs 4-6 min) Factor2: Emotion (Neutral vs Pain) | Neutral = Pain = 14 | Time x Group: F (2, 52) = 0.1607, P=0.8520  Time: F (1.768, 45.97) = 2.918, P=0.0702  Group: F (1, 26) = 0.03987, P=0.8433  Subject: F (26, 52) = 2.288, P=0.0056  Multiple comparison P: Neutral vs Pain: 0-2 min 0.9895 2-4 min 0.9697 4-6 min 0.9965 | | | |
| 4J | Sniffing time | Two-way RM ANOVA followed by Sidak's multiple comparisons test within each time session, comparing pain with neutral Factor1: Time (0-2 min vs 2-4 min vs 4-6 min) Factor2: Emotion (Neutral vs Pain) | Neutral = Pain = 14 | Time x Group: F (2, 52) = 2.753, P=0.0731  Time: F (1.968, 51.18) = 4.187, P=0.0212  Group: F (1, 26) = 11.50, P=0.0022  Subject: F (26, 52) = 1.525, P=0.0972   Multiple comparison P: Neutral vs Pain: 0-2 min 0.0018 2-4 min 0.0904 4-6 min 0.9180 | | | |
| 4K | Percentage of sniffing time in 0-2 min | Generalized linear mixed model  Fixed effects: Treatment× Emotion interaction (full factorial design)  Random effects: Subject-specific random intercepts (1\|subject)  Treatment (Saline vs CNO), Emotion (Neutral vs Pain) | Saline = 14 CNO = 14 | Significant group × emotion interaction:  Group × Emotion: F(1,52) = 18.52, P < 0.0001, β = 23.75, 95% CI [12.67, 34.82]  Simple effect (Neutral vs Pain):  Saline β = -1.85, t(26) = -0.45, P = 0.6599  CNO β = 21.90, t(26) = 5.93, P < 0.0001 | | | |
| 4L | Discrimination index | Unpaired t test | Saline = 14 CNO = 14 | t=2.932, df=26, two tailed P=0.0069 | | | |
| 4P | Mechanical pain threshold | Two-way RM ANOVA followed by Sidak's multiple comparisons test within each treatment, comparing post with baseline Factor1: Treatment (Light off vs Light on) Factor2: Time (Baseline vs Post) | Light off = 11 Light on = 11 | Treatment x Time: F (1, 20) = 5.602 P=0.0281 Treatment: F (1, 20) = 1.301 P=0.2676 Time: F (1, 20) = 3.299 P=0.0843 Subject: F (20, 20) = 1.062 P=0.4472  Multiple comparison P: Baseline vs Post: Light off 0.9107 Light on 0.0155 | | | |
| 4Q | Percentage of decreased mechanical pain threshold | Unpaired t test | Light off = 11 Light on = 11 | t=2.448, df=20, two tailed P=0.0237 | | | |
| 4R | Percentage of mice with decreased pain threshold | Fisher’s exact test | Light off = 11 Light on = 11 | P=0.1827 | | | |
| 5F | Mean z-score (evoked - baseline) | Paired t test | Obs-Ctrl = Obs-Pain = 8 | t=8.949, df=7, two tailed P<0.0001 | | | |
| 5G | AUC of ΔF/F | Paired t test | Obs-Ctrl = Obs-Pain = 8 | t=10.20, df=7, two tailed P<0.0001 | | | |
| 5K | Mechanical pain threshold | Two-way RM ANOVA followed by Sidak's multiple comparisons test within each treatment, comparing post with baseline Factor1: Treatment (Saline vs CNO) Factor2: Time (Baseline vs Post) | Saline = 11 CNO = 11 | Treatment x Time: F (1, 20) = 13.65 P=0.0014 Treatment: F (1, 20) = 3.980 P=0.0598 Time: F (1, 20) = 9.620 P=0.0056 Subject: F (20, 20) = 1.846 P=0.0896  Multiple comparison P: Baseline vs Post: Saline 0.0002 CNO 0.8971 | | | |
| 5L | Percentage of decreased mechanical pain threshold | Unpaired t test | Saline = 11 CNO = 11 | t=3.696, df=20, two tailed P=0.0014 | | | |
| 5M | Percentage of mice with decreased pain threshold | Fisher’s exact test | Saline = 11 CNO = 11 | P=0.0124 | | | |
| 5N | Percentage of sniffing time in 0-2 min | Generalized linear mixed model  Fixed effects: Group × Emotion interaction (full factorial design)  Random effects: Subject-specific random intercepts (1\|subject)  Group (Saline vs CNO), Emotion (Neutral vs Pain) | Saline = 10 CNO = 10 | Significant group × emotion interaction:  Group × Emotion: F(1,36) = 18.29, P < 0.0001, β = -23.22, 95% CI [-34.23, -12.21]  Simple effect (Neutral vs Pain):  Saline β = 20.62, t(18) = 6.12, P < 0.0001  CNO β = -2.59, t(18) = -0.61, P = 0.550 | | | |
| 5O | Discrimination index | Unpaired t test | Saline = 10 CNO = 10 | t=2.869, df=18, two tailed P=0.0102 | | | |
| 6F | Mean z-score (evoked - baseline) | Paired t test | Obs-Ctrl = Obs-Pain = 7 | t=0.6201, df=6, two tailed P=0.5580 | | | |
| 6G | AUC of ΔF/F | Paired t test | Obs-Ctrl = Obs-Pain = 7 | t=0.6820, df=6, two tailed P=0.5207 | | | |
| 6K | Mechanical pain threshold | Two-way RM ANOVA followed by Sidak's multiple comparisons test within each treatment, comparing post with baseline Factor1: Treatment (Saline vs CNO) Factor2: Time (Baseline vs Post) | Saline = 9 CNO = 8 | Treatment x Time: F (1, 15) = 19.36 P=0.0005 Treatment: F (1, 15) = 3.837 P=0.0690 Time: F (1, 15) = 12.03 P=0.0034 Subject: F (15, 15) = 2.440 P=0.0472  Multiple comparison P: Baseline vs Post: Saline 0.7573 CNO 0.0001 | | | |
| 6L | Percentage of decreased mechanical pain threshold | Unpaired t test | Saline = 9 CNO = 8 | t=4.201, df=15, two tailed P=0.0008 | | | |
| 6M | Percentage of mice with decreased pain threshold | Fisher’s exact test | Saline = 9 CNO = 8 | P=0.0090 | | | |
| 6N | Percentage of sniffing time in 0-2 min | Generalized linear mixed model  Fixed effects: Group × Emotion interaction (full factorial design)  Random effects: Subject-specific random intercepts (1\|subject)  Group (Saline vs CNO), Emotion (Neutral vs Pain) | Saline = 8 CNO = 8 | Significant group × emotion interaction:  Group × Emotion: F(1,28) = 10.56, P = 0.003, β = 14.86, 95% CI [5.49, 24.23]  Simple effect (Neutral vs Pain):  Saline β = 1.81, t(14) = 0.49, P = 0.633  CNO β = 16.67, t(14) = 6.26, P < 0.0001 | | | |
| 6O | Discrimination index | Unpaired t test | Saline = 8 CNO = 8 | t=2.149, df=14, two tailed P=0.0496 | | | |
| 7B | Mechanical pain threshold | Two-way RM ANOVA followed by Sidak's multiple comparisons test within each group, comparing post with baseline Factor1: Group (MS vs MS+ST) Factor2: Time (Baseline vs Post) | MS = 8 MS+ST = 9 | Group x Time: F (1, 15) = 3.774 P=0.0711 Group: F (1, 15) = 0.02313 P=0.8812 Time: F (1, 15) = 15.64 P=0.0013 Subject: F (15, 15) = 0.7992 P=0.6651  Multiple comparison P: Baseline vs Post: MS 0.3390 MS+ST 0.0013 | | | |
| 7C | Percentage of decreased mechanical pain threshold | Unpaired t test | MS = 8 MS+ST = 9 | t=2.397, df=15, two tailed P=0.0300 | | | |
| 7D | Percentage of mice with decreased pain threshold | Fisher’s exact test | MS = 8 MS+ST = 9 | P=0.0824 | | | |
| 7E | Percentage of sniffing time in 0-2 min | Generalized linear mixed model  Fixed effects: Group × Emotion interaction (full factorial design)  Random effects: Subject-specific random intercepts (1\|subject)  Group (MS vs MS+ST), Emotion (Neutral vs Pain) | MS = 11 MS+ST = 9 | Significant group × emotion interaction:  Group × Emotion: F(1,36) = 19.42, P < 0.0001, β = 28.98, 95% CI [15.64, 42.32]  Simple effect (Neutral vs Pain):  MS β = -5.96, t(20) = -1.53, P = 0.142  MS+ST β = 23.02, t(16) = 4.20, P = 0.0007 | | | |
| 7F | Discrimination index | Unpaired t test | MS = 11 MS+ST = 9 | t=2.956, df=18, two tailed P=0.0085 | | | |
| 7J | Rheobase | Welch's t test | MS = 20 cells  MS+ST = 10 cells | t=5.460, df=22.84, two tailed P<0.0001 | | | |
| 7K | AP threshold | Unpaired t test | MS = 20 cells  MS+ST = 10 cells | t=1.195, df=28, two tailed P=0.2423 | | | |
| 7M | Spike frequency | Two-way RM ANOVA  Factor1: Injected currents Factor2: Group (MS vs MS+ST) | MS = 15 cells  MS+ST = 11 cells | Inject currents x Group: F (11, 264) = 2.524 P=0.0049 Inject currents: F (11, 264) = 169.7 P<0.0001 Group: F (1, 24) = 5.176 P=0.0321 Subject: F (24, 264) = 22.50 P<0.0001 | | | |
| 7Q | Mechanical pain threshold | Two-way RM ANOVA followed by Sidak's multiple comparisons test within each treatment, comparing post with baseline Factor1: Treatment (Saline vs CNO) Factor2: Time (Baseline vs Post) | Saline = 8 CNO = 8 | Treatment x Time: F (1, 14) = 8.950 P=0.0097 Treatment: F (1, 14) = 0.002226 P=0.9630 Time: F (1, 14) = 14.72 P=0.0018 Subject: F (14, 14) = 2.969 P=0.0253  Multiple comparison P: Baseline vs Post: Saline 0.0005 CNO 0.8060 | | | |
| 7R | Percentage of decreased mechanical pain threshold | Unpaired t test | Saline = 8 CNO = 8 | t=2.633, df=14, two tailed P=0.0197 | | | |
| 7S | Percentage of mice with decreased pain threshold | Fisher’s exact test | Saline = 8 CNO = 8 | P=0.2000 | | | |
| S1B | Latency | Two-way RM ANOVA followed by Sidak's multiple comparisons test within each group, comparing post with baseline Factor1: Group (AFR vs MS) Factor2: Degree (50℃ vs 52℃ vs 56℃) | AFR = 15  MS = 14 | Group x Degree: F (2, 54) = 7.562 P=0.0013 Group: F (1, 27) = 0.2542 P=0.6182 Degress: F (2, 54) = 126.5 P<0.0001 Subject: F (27, 54) = 2.275 P=0.0051  Multiple comparison P: AFR vs MS: 50℃ 0.0739 52℃ 0.1669  56℃ 0.3769 | | | |
| S1C | Duration of nociceptive behaviors | Unpaired t test | AFR = 13 MS =11 | t=0.1659, df=22, two tailed P=0.8697 | | | |
| S2B | Mechanical pain threshold | Two-way RM ANOVA followed by Sidak's multiple comparisons test within each group, comparing post with baseline Factor1: Group (AFR vs MS (2-7)) Factor2: Time (Baseline vs Post) | AFR = 10 MS (2-7) =10 | Group x Time: F (1, 18) = 2.670 P=0.1196 Group: F (1, 18) = 0.01420 P=0.9065 Time: F (1, 18) = 10.11 P=0.0052 Subject: F (18, 18) = 09648 P=0.5298  Multiple comparison P: Baseline vs Post: AFR 0.0063 MS (2-7) 0.4945 | | | |
| S2C | Percentage of decreased mechanical pain threshold | Unpaired t test | AFR = 10 MS (2-7) =10 | t=2.114, df=18, two tailed P=0.0487 | | | |
| S2D | Percentage of mice with decreased pain threshold | Fisher’s exact test | AFR = 10 MS (2-7) =10 | P=0.1409 | | | |
| S2F | Mechanical pain threshold | Two-way RM ANOVA followed by Sidak's multiple comparisons test within each group, comparing post with baseline Factor1: Group (AFR vs MS (2-7)) Factor2: Time (Baseline vs Post) | AFR = 8 MS (15-21) =8 | Group x Time: F (1, 14) = 0.6847 P=0.4210 Group: F (1, 14) = 0.1101 P=0.7449 Time: F (1, 14) = 30.92 P<0.0001 Subject: F (14, 14) = 2.359 P=0.0600  Multiple comparison P: Baseline vs Post: AFR 0.00096  MS (15-21) 0.0096 | | | |
| S2G | Percentage of decreased mechanical pain threshold | Unpaired t test | AFR = 8 MS (15-21)=8 | t=0.9520, df=14, two tailed P=0.3572 | | | |
| S2H | Percentage of mice with decreased pain threshold | Fisher’s exact test | AFR = 8 MS (15-21)=8 | P=0.4667 | | | |
| S2J | Mechanical pain threshold | Two-way RM ANOVA followed by Sidak's multiple comparisons test within each group, comparing post with baseline Factor1: Group (AFR vs MS) Factor2: Time (Baseline vs Post) | AFR = 11 MS =12 | Group x Time: F (1, 21) = 7.992 P=0.0101 Group: F (1, 21) = 2.148 P=0.1575 Time: F (1, 21) = 46.24 P<0.0001 Subject: F (21, 21) = 1.688 P=0.1192  Multiple comparison P: Baseline vs Post: AFR <0.0001 MS 0.0182  AFR vs MS  Post 0.0122 | | | |
| S2K | Percentage of decreased mechanical pain threshold | Unpaired t test | AFR = 11 MS =12 | t=3.186, df=21, two tailed P=0.0044 | | | |
| S2L | Percentage of mice with decreased pain threshold | Fisher’s exact test | AFR = 11 MS =12 | P=0.4783 | | | |
| S3A | Mechanical pain threshold | Two-way RM ANOVA followed by Sidak's multiple comparisons test within each group, comparing post with baseline Factor1: Group (AFR vs MS) Factor2: Time (Baseline vs Post) | AFR = 9 MS =10 | Group x Time: F (1, 17) = 4.557 P=0.0476 Group: F (1, 17) = 2.300 P=0.1477 Time: F (1, 17) = 18.64 P=0.0005 Subject: F (17, 17) = 2.505 P=0.0333  Multiple comparison P: Baseline vs Post: AFR 0.0007 MS 0.2453 | | | |
| S3B | Percentage of decreased mechanical pain threshold | Unpaired t test | AFR = 9 MS =10 | t=3.219, df=17, two tailed P=0.0050 | | | |
| S3C | Percentage of mice with decreased pain threshold | Fisher's exact test | AFR = 9 MS =10 | P=0.0573 | | | |
| S3D | Percentage of sniffing time in 0-2 min | Generalized linear mixed model  Fixed effects: Group × Emotion interaction (full factorial design)  Random effects: Subject-specific random intercepts (1\|subject)  Group (AFR vs MS), Emotion (Neutral vs Pain) | AFR = 10 MS = 11 | Significant group × emotion interaction:  Group × Emotion: F(1,38) = 16.72, P < 0.001, β = -29.89, 95% CI [-44.69, -15.09]  Simple effect (Neutral vs Pain):  AFR β = 32.41, t(18) = 7.31, P < 0.0001  MS β = 2.51, t(20) = 0.44, P = 0.663 | | | |
| S3E | Discrimination index | Unpaired t test | AFR = 10 MS = 11 | t=2.750, df=19, two tailed P=0.0127 | | | |
| S4C | Percentage of time in open arm | Unpaired t test | AFR = 12 MS = 14 | t=0.9947, df=24, two tailed P=0.3298 | | | |
| S4D | Percentage of time in closed arm | Unpaired t test | AFR = 12 MS = 14 | t=0.6960, df=24, two tailed P=0.4931 | | | |
| S4E | Entries of open arm | Unpaired t test | AFR = 12 MS = 14 | t=1.176, df=24, two tailed P=0.2512 | | | |
| S4G | Percentage of time in center | Unpaired t test | AFR = 10 MS = 10 | t=0.08192, df=18, two tailed P=0.9356 | | | |
| S4H | Percentage of time in corner | Unpaired t test | AFR = 10 MS = 10 | t=1.897, df=18, two tailed P=0.0739 | | | |
| S4I | Total distance | Unpaired t test | AFR = 10 MS = 10 | t=1.661, df=18, two tailed P=0.1139 | | | |
| S4K | Sniffing time | Two-way RM ANOVA followed by Sidak's multiple comparisons test within each group, comparing stranger1 with empty wire cup Factor1: Group (AFR vs MS) Factor2: Stimulus (S1 vs E) | AFR = 11 MS = 10 | Group x Stimulus: F (1, 19) = 0.003783 P=0.9516 Group: F (1, 19) = 0.5024 P=0.4871 Stimulus: F (1, 19) = 34.64 P<0.0001 Subject: F (19, 19) = 1.430 P=0.2215  Multiple comparison P: Baseline vs Post: AFR 0.0008 MS 0.0015 | | | |
| S4L | Sociability index | Welch's t test | AFR = 11 MS = 10 | t=0.1446, df=12.57, two tailed P=0.8873 | | | |
| S4N | Sniffing time | Two-way RM ANOVA followed by Sidak's multiple comparisons test within each group, comparing stranger1 with stranger2 Factor1: Group (AFR vs MS) Factor2: Stimulus (S1 vs S2) | AFR = 11 MS = 10 | Group x Stimulus: F (1, 19) = 2.315e-005 P=0.9962 Group: F (1, 19) = 0.02306 P=0.8809 Stimulus: F (1, 19) = 60.02 P<0.0001 Subject: F (19, 19) = 1.335 P=0.2675  Multiple comparison P: Baseline vs Post: AFR <0.0001 MS <0.0001 | | | |
| S4O | Social novelty index | Unpaired t test | AFR = 11 MS = 10 | t=0.5152, df=19, two tailed P=0.6123 | | | |
| S5D | Number of tdTomato^+^ cell per mm^2^ | Two-way ANOVA followed by Sidak's multiple comparisons test within each group, comparing pain with neutral Factor1: Group (AFR vs MS) Factor2: Emotion (Obs-Ctrl vs Obs-Pain) | AFR: Obs-Ctrl = 3  Obs-Pain = 3 for PAG  Obs-Ctrl = 4  Obs-Pain = 4 for other brain regions MS: Obs-Ctrl = 3  Obs-Pain = 3 | Multiple comparison  Obs-Ctrl vs Obs-Pain | | P value | |
|  |  |  |  | OFC | AFR | 0.0178 | |
|  |  |  |  |  | MS | 0.0497 | |
|  |  |  |  | mPFC | AFR | <0.0001 | |
|  |  |  |  |  | MS | <0.0001 | |
|  |  |  |  | ACC | AFR | 0.0002 | |
|  |  |  |  |  | MS | 0.0008 | |
|  |  |  |  | IC | AFR | <0.0001 | |
|  |  |  |  |  | MS | 0.0001 | |
|  |  |  |  | NAc | AFR | 0.0111 | |
|  |  |  |  |  | MS | 0.0467 | |
|  |  |  |  | MPOA | AFR | <0.0001 | |
|  |  |  |  |  | MS | 0.0033 | |
|  |  |  |  | PVN | AFR | 0.0002 | |
|  |  |  |  |  | MS | 0.5144 | |
|  |  |  |  | PVT | AFR | <0.0001 | |
|  |  |  |  |  | MS | 0.4414 | |
|  |  |  |  | Amg | AFR | 0.3396 | |
|  |  |  |  |  | MS | 0.1287 | |
|  |  |  |  | PAG | AFR | 0.9886 | |
|  |  |  |  |  | MS | 0.2432 | |
| S5D | Number of tdTomato^+^ cell per mm^2^ | Two-way ANOVA followed by Sidak's multiple comparisons test within each group, comparing pain with neutral Factor1: Group (AFR vs MS) Factor2: Emotion (Neutral vs Pain) | AFR: Obs-Ctrl = 3  Obs-Pain = 3 for PAG  Obs-Ctrl = 4  Obs-Pain = 4 for other brain regions MS: Obs-Ctrl = 3  Obs-Pain = 3 | Multiple comparison  AFR vs MS | | P value | |
|  |  |  |  | OFC | Obs-Ctrl | 0.0647 | |
|  |  |  |  |  | Obs-Pain | 0.0462 | |
|  |  |  |  | mPFC | Obs-Ctrl | <0.0001 | |
|  |  |  |  |  | Obs-Pain | <0.0001 | |
|  |  |  |  | ACC | Obs-Ctrl | 0.0013 | |
|  |  |  |  |  | Obs-Pain | 0.0009 | |
|  |  |  |  | IC | Obs-Ctrl | 0.0016 | |
|  |  |  |  |  | Obs-Pain | 0.0021 | |
|  |  |  |  | NAc | Obs-Ctrl | 0.0004 | |
|  |  |  |  |  | Obs-Pain | 0.0002 | |
|  |  |  |  | MPOA | Obs-Ctrl | 0.0005 | |
|  |  |  |  |  | Obs-Pain | <0.0001 | |
|  |  |  |  | PVN | Obs-Ctrl | 0.0656 | |
|  |  |  |  |  | Obs-Pain | 0.0950 | |
|  |  |  |  | PVT | Obs-Ctrl | 0.9892 | |
|  |  |  |  |  | Obs-Pain | <0.0001 | |
|  |  |  |  | Amg | Obs-Ctrl | 0.1660 | |
|  |  |  |  |  | Obs-Pain | 0.5603 | |
|  |  |  |  | PAG | Obs-Ctrl | 0.2271 | |
|  |  |  |  |  | Obs-Pain | 0.9793 | |
| S6D | Velocity | Unpaired t test | Saline = 11 CNO = 11 | t=0.9821, df=20, two tailed P=0.3378 | | | |
| S6E | Total distance | Unpaired t test | Saline = 11 CNO = 11 | t=0.9818, df=20, two tailed P=0.3379 | | | |
| S6F | Percentage of time in center | Unpaired t test | Saline = 11 CNO = 11 | t=0.4545, df=20, two tailed P=0.6544 | | | |
| S6G | Percentage of time in corner | Unpaired t test | Saline = 11 CNO = 11 | t=1.046, df=20, two tailed P=0.3079 | | | |
| S7D | Mechanical pain threshold | Two-way RM ANOVA followed by Sidak's multiple comparisons test within each treatment, comparing post with baseline Factor1: Treatment (Saline vs CNO) Factor2: Time (Baseline vs Post) | Saline = 12 CNO = 12 | Treatment x Time: F (1, 22) = 3.721 P=0.0667 Treatment: F (1, 22) = 2.305 P=0.1432 Time: F (1, 22) = 21.20 P=0.0001 Subject: F (22, 22) = 2.317 P=0.0274  Multiple comparison P: Baseline vs Post: Saline 0.0003 CNO 0.1383 | | | |
| S7E | Percentage of decreased mechanical pain threshold | Unpaired t test | Saline = 12 CNO = 12 | t=2.546, df=22, two tailed P=0.0184 | | | |
| S7F | Percentage of mice with decreased pain threshold | Fisher’s exact test | Saline = 12 CNO = 12 | P=0.3168 | | | |
| S8D | Mechanical pain threshold | Two-way RM ANOVA followed by Sidak's multiple comparisons test within each treatment, comparing post with baseline Factor1: Treatment (Saline vs CNO) Factor2: Time (Baseline vs Post) | Saline = 9 CNO = 9 | Treatment x Time: F (1, 16) = 0.6187 P=0.4430 Treatment: F (1, 16) = 0.1287 P=0.7244 Time: F (1, 16) = 58.17 P<0.0001 Subject: F (16, 16) = 1.569 P=0.1884  Multiple comparison P: Baseline vs Post: Saline <0.0001 CNO 0.0004 | | | |
| S8E | Percentage of decreased mechanical pain threshold | Unpaired t test | Saline = 9 CNO = 9 | t=0.8495, df=16, two tailed P=0.4081 | | | |
| S8F | Percentage of mice with decreased pain threshold | Fisher’s exact test | Saline = 9 CNO = 9 | P>0.9999 | | | |
| S9D | Mechanical pain threshold | Two-way RM ANOVA followed by Sidak's multiple comparisons test within each treatment, comparing post with baseline Factor1: Treatment (Light off vs Light on) Factor2: Time (Baseline vs Post) | Light off = 8 Light on = 8 | Treatment x Time: F (1, 14) = 81.41 P<0.0001 Treatment: F (1, 14) = 5.937 P=0.0288 Time: F (1, 14) = 72.76 P<0.0007 Subject: F (14, 14) = 5.144 P=0.0021  Multiple comparison P: Baseline vs Post: Light off <0.0001 Light on 0.9285 | | | |
| S9E | Percentage of decreased mechanical pain threshold | Unpaired t test | Light off = 8 Light on = 8 | t=8.613, df=14, two tailed P<0.0001 | | | |
| S9F | Percentage of mice with decreased pain threshold | Fisher’s exact test | Light off = 8 Light on = 8 | P=0.0070 | | | |
| S10C up | Mechanical pain threshold | Paired t test | Light off = Light on = 8 | t=0.2820, df=7, two tailed P=0.7861 | | | |
| S10C down | Mechanical pain threshold | Paired t test | Light off = Light on = 8 | t=0.8324, df=7, two tailed P=0.4325 | | | |
| S10D up left | Latency | Paired t test | Light off = Light on = 6 | t=1.298, df=5, two tailed P=0.2509 | | | |
| S10D up middle | Latency | Paired t test | Light off = Light on = 6 | t=1.466, df=5, two tailed P=0.2025 | | | |
| S10D up right | Latency | Paired t test | Light off = Light on = 6 | t=0.9482, df=5, two tailed P=0.3866 | | | |
| S10D down left | Latency | Paired t test | Light off = Light on = 7 | t=0.6316, df=6, two tailed P=0.5510 | | | |
| S10D down middle | Latency | Paired t test | Light off = Light on = 7 | t=0.9482, df=5, two tailed P=0.0989 | | | |
| S10D down right | Latency | Paired t test | Light off = Light on = 7 | t=0.5570, df=5, two tailed P=0.5977 | | | |
| S11D | Mechanical pain threshold | Two-way RM ANOVA followed by Sidak's multiple comparisons test within each treatment, comparing post with baseline Factor1: Treatment (mCherry vs hM4Di) Factor2: Time (Baseline vs Post) | mCherry = 9 hM4Di = 10 | Treatment x Time: F (1, 17) = 0.04658 P=0.8317 Treatment: F (1, 17) = 0.5366 P=0.4738 Time: F (1, 17) = 58.73 P<0.0001 Subject: F (17, 17) = 3.105 P=0.0124  Multiple comparison P: Baseline vs Post: Saline 0.0002 CNO <0.0001 | | | |
| S11E | Percentage of decreased mechanical pain threshold | Unpaired t test | mCherry = 9 hM4Di = 10 | t=0.3119, df=17, two tailed P=0.7589 | | | |
| S11F | Percentage of mice with decreased pain threshold | Fisher’s exact test | mCherry = 9 hM4Di = 10 | P>0.9999 | | | |
| S12B | Rheobase | Welch's t test | AFR = 23 cells  MS = 24 cells | t=0.9790, df=38.08, two tailed P=0.3338 | | | |
| S12C | AP threshold | Welch's t test | AFR = 23 cells  MS = 24 cells | t=0.4378, df=31.23, two tailed P=0.6646 | | | |
| S12E | Spike frequency | Two-way RM ANOVA  Factor1: Injected currents Factor2: Group (AFR vs MS) | AFR = 22 cells  MS = 25 cells | Inject currents x Group: F (11, 495) = 1.081 P=0.3749 Inject currents: F (11, 495) = 448.2 P<0.0001 Group: F (1, 45) = 0.2531 P=0.6173 Subject: F (45, 495) = 16.87 P<0.0001 | | | |
| S12G-right | Frequency | Unpaired t test | AFR = 17 cells  MS = 21 cells | t=1.771, df=36, two tailed P=0.0850 | | | |
| S12H-right | Amplitude | Welch's t test | AFR = 17 cells  MS = 21 cells | t=1.167, df=31.61, two tailed P=0.2519 | | | |
| S13D | Mechanical pain threshold | Two-way RM ANOVA followed by Sidak's multiple comparisons test within each treatment, comparing post with baseline Factor1: Treatment (Saline vs CNO) Factor2: Time (Baseline vs Post) | Saline = 12 CNO = 12 | Treatment x Time: F (1, 22) = 0.1340 P=0.7179 Treatment: F (1, 22) = 0.08952 P=0.7676 Time: F (1, 22) = 36.55 P<0.0001 Subject: F (22, 22) = 1.496 P=0.1758  Multiple comparison P: Baseline vs Post: Saline 0.0012 CNO 0.0003 | | | |
| S13E | Percentage of decreased mechanical pain threshold | Unpaired t test | Saline = 12 CNO = 12 | t=0.8139, df=22, two tailed P=0.4244 | | | |
| S13F | Percentage of mice with decreased pain threshold | Fisher’s exact test | Saline = 12 CNO = 12 | P>0.9999 | | | |
| S14C | Normalized projection intensity | Unpaired t test for each brain region | Obs-Ctrl = 3  Obs-Pain = 3 |  | t | df | P value |
|  |  |  |  | mPFC | 14.86 | 16 | <0.0001 |
|  |  |  |  | NAc | 13.04 | 16 | <0.0001 |
|  |  |  |  | BNST | 0.4348 | 16 | 0.6695 |
|  |  |  |  | Amg | 0.1373 | 16 | 0.8925 |
| S15D | Mechanical pain threshold | Two-way RM ANOVA followed by Sidak's multiple comparisons test within each treatment, comparing post with baseline Factor1: Treatment (Saline vs CNO) Factor2: Time (Baseline vs Post) | Saline = 8 CNO = 8 | Treatment x Time: F (1, 14) = 0.6071 P=0.4489 Treatment: F (1, 14) = 0.1076 P=0.7478 Time: F (1, 14) = 23.46 P=0.0003 Subject: F (14, 14) = 0.8348 P=0.6299  Multiple comparison P: Baseline vs Post: Saline 0.0028 CNO 0.0244 | | | |
| S15E | Percentage of decreased mechanical pain threshold | Unpaired t test | Saline = 8 CNO = 8 | t=0.9133, df=14, two tailed P=0.3766 | | | |
| S15F | Percentage of mice with decreased pain threshold | Fisher’s exact test | Saline = 8 CNO = 8 | P>0.9999 | | | |
| S16G | Mechanical pain threshold | Two-way RM ANOVA followed by Sidak's multiple comparisons test within each treatment, comparing post with baseline Factor1: Treatment (Saline vs CNO) Factor2: Time (Baseline vs Post) | Saline = 7 CNO = 7 | Treatment x Time: F (1, 12) = 0.8423 P=0.3768 Treatment: F (1, 12) = 0.3214 P=0.5812 Time: F (1, 12) = 24.70 P=0.0003 Subject: F (12, 12) = 1.100 P=0.4359  Multiple comparison P: Baseline vs Post: Saline 0.0026 CNO 0.0282 | | | |
| S16H | Percentage of decreased mechanical pain threshold | Unpaired t test | Saline = 7 CNO = 7 | t=0.2329, df=12, two tailed P=0.8198 | | | |
| S16I | Percentage of mice with decreased pain threshold | Fisher’s exact test | Saline = 7 CNO = 7 | P>0.9999 | | | |
| S17A | Mechanical pain threshold | Two-way RM ANOVA followed by Sidak's multiple comparisons test within each group, comparing post with baseline Factor1: Group (MS vs MS+ST) Factor2: Time (Baseline vs Post) | MS = 7 MS+ST = 8 | Group x Time: F (1, 13) = 17.44 P=0.0011 Group: F (1, 13) = 14.10 P=0.0024 Time: F (1, 13) = 4.525 P=0.0531 Subject: F (13, 13) = 5.242 P=0.0027  Multiple comparison P: Baseline vs Post: MS 0.3343 MS+ST 0.0010 | | | |
| S17B | Percentage of decreased mechanical pain threshold | Unpaired t test | MS = 7 MS+ST = 8 | t=4.508, df=13, two tailed P=0.0006 | | | |
| S17C | Percentage of mice with decreased pain threshold | Fisher’s exact test | MS = 7 MS+ST = 8 | P=0.7690 | | | |
| S17D | Percentage of sniffing time in 0-2 min | Generalized linear mixed model  Fixed effects: Group × Emotion interaction (full factorial design)  Random effects: Subject-specific random intercepts (1\|subject)  Group (MS vs MS+ST), Emotion (Neutral vs Pain) | MS = 11 MS+ST = 9 | Significant group × emotion interaction:  Group × Emotion: F(1,36) = 10.18, P = 0.003, β = 19.94, 95% CI [7.27, 32.61]  Simple effect (Neutral vs Pain):  MS β = 1.21, t(20) = 0.26, P = 0.797  MS+ST β = 21.15, t(16) = 5.38, P < 0.0001 | | | |
| S17E | Discrimination index | Unpaired t test | MS = 11 MS+ST = 9 | t=2.141, df=18, two tailed P=0.0463 | | | |
| S18B | Mechanical pain threshold | Two-way RM ANOVA followed by Sidak's multiple comparisons test within each group, comparing post with baseline Factor1: Group (AFR vs AFR+ST) Factor2: Time (Baseline vs Post) | AFR = 14 AFR+ST = 12 | Group x Time: F (1, 24) = 1.644 P=0.2120 Group: F (1, 24) = 1.544 P=0.2260 Time: F (1, 24) = 36.98 P<0.0001 Subject: F (24, 24) = 1.170 P=0.3521  Multiple comparison P: Baseline vs Post: AFR 0.0034 AFR+ST <0.0001 | | | |
| S18C | Percentage of decreased mechanical pain threshold | Unpaired t test | AFR = 14 AFR+ST = 12 | t=0.7808, df=24, two tailed P=0.4426 | | | |
| S18D | Percentage of mice with decreased pain threshold | Fisher’s exact test | AFR = 14 AFR+ST = 12 | P=0.3304 | | | |
| S19F | Mean z-score (evoked - baseline) | Paired t test | Obs-Ctrl = Obs-Pain = 6 | t=2.658, df=5, two tailed P=0.0450 | | | |
| S19G | AUC of ΔF/F | Paired t test | Obs-Ctrl = Obs-Pain = 6 | t=2.616, df=5, two tailed P=0.0473 | | | |
